# The FLUXNET2015 dataset and the ONEFlux processing pipeline for eddy covariance data

**DOI:** 10.1038/s41597-020-0534-3

**Published:** 2020-07-09

**Authors:** Gilberto Pastorello, Carlo Trotta, Eleonora Canfora, Housen Chu, Danielle Christianson, You-Wei Cheah, Cristina Poindexter, Jiquan Chen, Abdelrahman Elbashandy, Marty Humphrey, Peter Isaac, Diego Polidori, Alessio Ribeca, Catharine van Ingen, Leiming Zhang, Brian Amiro, Christof Ammann, M. Altaf Arain, Jonas Ardö, Timothy Arkebauer, Stefan K. Arndt, Nicola Arriga, Marc Aubinet, Mika Aurela, Dennis Baldocchi, Alan Barr, Eric Beamesderfer, Luca Belelli Marchesini, Onil Bergeron, Jason Beringer, Christian Bernhofer, Daniel Berveiller, Dave Billesbach, Thomas Andrew Black, Peter D. Blanken, Gil Bohrer, Julia Boike, Paul V. Bolstad, Damien Bonal, Jean-Marc Bonnefond, David R. Bowling, Rosvel Bracho, Jason Brodeur, Christian Brümmer, Nina Buchmann, Benoit Burban, Sean P. Burns, Pauline Buysse, Peter Cale, Mauro Cavagna, Pierre Cellier, Shiping Chen, Isaac Chini, Torben R. Christensen, James Cleverly, Alessio Collalti, Claudia Consalvo, Bruce D. Cook, David Cook, Carole Coursolle, Edoardo Cremonese, Peter S. Curtis, Ettore D’Andrea, Humberto da Rocha, Xiaoqin Dai, Kenneth J. Davis, Bruno De Cinti, Agnes de Grandcourt, Anne De Ligne, Raimundo C. De Oliveira, Nicolas Delpierre, Ankur R. Desai, Carlos Marcelo Di Bella, Paul di Tommasi, Han Dolman, Francisco Domingo, Gang Dong, Sabina Dore, Pierpaolo Duce, Eric Dufrêne, Allison Dunn, Jiří Dušek, Derek Eamus, Uwe Eichelmann, Hatim Abdalla M. ElKhidir, Werner Eugster, Cacilia M. Ewenz, Brent Ewers, Daniela Famulari, Silvano Fares, Iris Feigenwinter, Andrew Feitz, Rasmus Fensholt, Gianluca Filippa, Marc Fischer, John Frank, Marta Galvagno, Mana Gharun, Damiano Gianelle, Bert Gielen, Beniamino Gioli, Anatoly Gitelson, Ignacio Goded, Mathias Goeckede, Allen H. Goldstein, Christopher M. Gough, Michael L. Goulden, Alexander Graf, Anne Griebel, Carsten Gruening, Thomas Grünwald, Albin Hammerle, Shijie Han, Xingguo Han, Birger Ulf Hansen, Chad Hanson, Juha Hatakka, Yongtao He, Markus Hehn, Bernard Heinesch, Nina Hinko-Najera, Lukas Hörtnagl, Lindsay Hutley, Andreas Ibrom, Hiroki Ikawa, Marcin Jackowicz-Korczynski, Dalibor Janouš, Wilma Jans, Rachhpal Jassal, Shicheng Jiang, Tomomichi Kato, Myroslava Khomik, Janina Klatt, Alexander Knohl, Sara Knox, Hideki Kobayashi, Georgia Koerber, Olaf Kolle, Yoshiko Kosugi, Ayumi Kotani, Andrew Kowalski, Bart Kruijt, Julia Kurbatova, Werner L. Kutsch, Hyojung Kwon, Samuli Launiainen, Tuomas Laurila, Bev Law, Ray Leuning, Yingnian Li, Michael Liddell, Jean-Marc Limousin, Marryanna Lion, Adam J. Liska, Annalea Lohila, Ana López-Ballesteros, Efrén López-Blanco, Benjamin Loubet, Denis Loustau, Antje Lucas-Moffat, Johannes Lüers, Siyan Ma, Craig Macfarlane, Vincenzo Magliulo, Regine Maier, Ivan Mammarella, Giovanni Manca, Barbara Marcolla, Hank A. Margolis, Serena Marras, William Massman, Mikhail Mastepanov, Roser Matamala, Jaclyn Hatala Matthes, Francesco Mazzenga, Harry McCaughey, Ian McHugh, Andrew M. S. McMillan, Lutz Merbold, Wayne Meyer, Tilden Meyers, Scott D. Miller, Stefano Minerbi, Uta Moderow, Russell K. Monson, Leonardo Montagnani, Caitlin E. Moore, Eddy Moors, Virginie Moreaux, Christine Moureaux, J. William Munger, Taro Nakai, Johan Neirynck, Zoran Nesic, Giacomo Nicolini, Asko Noormets, Matthew Northwood, Marcelo Nosetto, Yann Nouvellon, Kimberly Novick, Walter Oechel, Jørgen Eivind Olesen, Jean-Marc Ourcival, Shirley A. Papuga, Frans-Jan Parmentier, Eugenie Paul-Limoges, Marian Pavelka, Matthias Peichl, Elise Pendall, Richard P. Phillips, Kim Pilegaard, Norbert Pirk, Gabriela Posse, Thomas Powell, Heiko Prasse, Suzanne M. Prober, Serge Rambal, Üllar Rannik, Naama Raz-Yaseef, David Reed, Victor Resco de Dios, Natalia Restrepo-Coupe, Borja R. Reverter, Marilyn Roland, Simone Sabbatini, Torsten Sachs, Scott R. Saleska, Enrique P. Sánchez-Cañete, Zulia M. Sanchez-Mejia, Hans Peter Schmid, Marius Schmidt, Karl Schneider, Frederik Schrader, Ivan Schroder, Russell L. Scott, Pavel Sedlák, Penélope Serrano-Ortíz, Changliang Shao, Peili Shi, Ivan Shironya, Lukas Siebicke, Ladislav Šigut, Richard Silberstein, Costantino Sirca, Donatella Spano, Rainer Steinbrecher, Robert M. Stevens, Cove Sturtevant, Andy Suyker, Torbern Tagesson, Satoru Takanashi, Yanhong Tang, Nigel Tapper, Jonathan Thom, Frank Tiedemann, Michele Tomassucci, Juha-Pekka Tuovinen, Shawn Urbanski, Riccardo Valentini, Michiel van der Molen, Eva van Gorsel, Ko van Huissteden, Andrej Varlagin, Joseph Verfaillie, Timo Vesala, Caroline Vincke, Domenico Vitale, Natalia Vygodskaya, Jeffrey P. Walker, Elizabeth Walter-Shea, Huimin Wang, Robin Weber, Sebastian Westermann, Christian Wille, Steven Wofsy, Georg Wohlfahrt, Sebastian Wolf, William Woodgate, Yuelin Li, Roberto Zampedri, Junhui Zhang, Guoyi Zhou, Donatella Zona, Deb Agarwal, Sebastien Biraud, Margaret Torn, Dario Papale

**Affiliations:** 1 Computational Research Division, Lawrence Berkeley National Laboratory, Berkeley, CA 94720 USA; 2 DIBAF, University of Tuscia, Viterbo, 01100 Italy; 3 Euro-Mediterranean Centre on Climate Change Foundation (CMCC), Lecce, 73100 Italy; 4 Climate & Ecosystem Sciences Division, Lawrence Berkeley National Laboratory, Berkeley, CA 94720 USA; 5 Department of Civil Engineering, California State University, Sacramento, CA 95819 USA; 6 Department of Geography, Environment, and Spatial Sciences, Michigan State University, East Lansing, MI 48823 USA; 7 Department of Computer Science, University of Virginia, Charlottesville, VA 22904 USA; 8 TERN Ecosystrem Processes, Menzies Creek, VIC3159 Australia; 9 Key Laboratory of Ecosystem Network Observation and Modeling, Institute of Geographic Sciences and Natural Resources Research, Chinese Academy of Sciences, Beijing, 100101 China; 10 Department of Soil Science, University of Manitoba, Winnipeg, MB, R3T2N2 Canada; 11 Department of Agroecology and Environment, Agroscope Research Institute, Zürich, 8046 Switzerland; 12 School of Geography and Earth Sciences, McMaster University, L8S4K1, Hamilton, ON, Canada; 13 Department of Physical Geography and Ecosystem Science, Lund University, Lund, 22362 Sweden; 14 Department of Agronomy and Horticulture, University of Nebraska-Lincoln, Lincoln, NE 68583 USA; 15 School of Ecosystem and Forest Sciences, The University of Melbourne, Richmond, VIC3121 Australia; 16 Department of Biology, Research Group PLECO, University of Antwerp, Antwerp, 2610 Belgium; 17 Joint Research Centre, European Commission, Ispra, 21027 Italy; 18 TERRA Teaching and Research Center, University of Liege, Gembloux, B-5030 Belgium; 19 Finnish Meteorological Institute, Helsinki, 00560 Finland; 20 ESPM, University of California Berkeley, Berkeley, CA 94720 USA; 21 Global Institute for Water Security, University of Saskatchewan, Saskatoon, SK, S7N3H5 Canada; 22 Climate Research Division, Environment and Climate Change Canada, Saskatoon, SK, S7N3H5 Canada; 23 Department of Sustainable Agro-ecosystems and Bioresources, Research and Innovation Centre, Fondazione Edmund Mach, San Michele All’adige, 38010 Italy; 24 Department of Landscape Design and Sustainable Ecosystems, Agrarian‐Technological Institute, RUDN University, Moscow, 117198 Russia; 25 Direction du marché du carbone, Ministère du Développement durable de l’Environnement et de la Lutte contre les changements climatiques, Québec, QC, G1R5V7 Canada; 26 School of Agriculture and Environment, University of Western Australia, Crawley, 6009 Australia; 27 Institute of Hydrology and Meteorology, Technische Universität Dresden, Tharandt, 01737 Germany; 28 Université Paris-Saclay, CNRS, AgroParisTech, Ecologie Systématique et Evolution, Orsay, 91405 France; 29 Biological Systems Engineering, University of Nebraska-Lincoln, Lincoln, NE 68583 USA; 30 Faculty of Land and Food Systems, University of British Columbia, Vancouver, BC, V6T1Z4 Canada; 31 Department of Geography, University of Colorado, Boulder, CO 80309 USA; 32 Department of Civil, Environmental & Geodetic Engineering, Ohio State University, Columbus, OH 43210 USA; 33 Alfred Wegener Institute Helmholtz Centre for Polar and Marine Research, Potsdam, 14482 Germany; 34 Geography Department, Humboldt-Universität zu Berlin, Berlin, Germany; 35 Forest Resources, University of Minnesota, St Paul, MN 55108 USA; 36 Université de Lorraine, AgroParisTech, INRAE, UMR Silva, Nancy, 54000 France; 37 ISPA, Bordeaux Sciences Agro, INRAE, Villenave d’Ornon, 33140 France; 38 School of Biological Sciences, University of Utah, Salt Lake City, UT 84112 USA; 39 School of Forest Resources and Conservation, University of Florida, Gainesville, FL 32611 USA; 40 McMaster University Library, McMaster University, Hamilton, ON, L8S4L6 Canada; 41 Thünen Institute of Climate-Smart Agriculture, Federal Research Institute of Rural Areas, Forestry and Fisheries, Braunschweig, 38116 Germany; 42 Department of Environmental Systems Science, ETH Zurich, Zurich, 8092 Switzerland; 43 INRAE UMR ECOFOG, Kourou, 97387 French Guiana; 44 Mesoscale and Microscale Meteorology Laboratory, National Center for Atmospheric Research, Boulder, CO 80301 USA; 45 Université Paris-Saclay, INRAE, AgroParisTech, UMR ECOSYS, Thiverval-Grignon, 78850 France; 46 Australian Landscape Trust, Renmark, SA5341 Australia; 47 State Key Laboratory of Vegetation and Environmental Change, Institute of Botany, Chinese Academy of Sciences, Beijing, 100093 China; 48 Department of Bioscience, Arctic Research Center, Aarhus University, Roskilde, 4000 Denmark; 49 School of Life Sciences, University of Technology Sydney, Sydney, 2007 Australia; 50 Terrestrial Ecosystem Research Network TERN, University of Technology, Sydney, 2007 Australia; 51 Institute for Agricultural and Forestry Systems in the Mediterranean, National Research Council of Italy, Ercolano, 80056 Italy; 52 Research Institute on Terrestrial Ecosystems, National Research Council of Italy, Porano, 05010 Italy; 53 Biospheric Sciences Laboratory, NASA Goddard Space Flight Center, Greenbelt, MD 20771 USA; 54 Environmental Science Division, Argonne National Laboratory, Lemont, IL 60439 USA; 55 Canadian Forest Service, Natural Resources Canada, Québec, QC, G1V4C7 Canada; 56 Centre d’étude de la forêt, Faculté de foresterie, de géographie et de géomatique, Université Laval, Québec, QC, G1V0A6 Canada; 57 Climate Change Unit, Environmental Protection Agency of Aosta Valley, Saint Christophe, 11020 Italy; 58 Department of Evolution, Ecology, and Organismal Biology, Ohio State University, Columbus, OH 43210 USA; 59 Instituto de Astronomia, Geofísica e Ciências Atmosféricas, Universidade de São Paulo, São Paulo, SP, 01000-000 Brazil; 60 Department of Meteorology and Atmospheric Science, The Pennsylvania State University, University Park, PA 16802 USA; 61 Institute of Research on Terrestrial Ecosystems, National Research Council of Italy, Montelibretti, 00010 Italy; 62 UMR Eco&Sols, CIRAD, Montpellier, 34060 France; 63 Pedology, Embrapa Amazonia Oriental, Belém, PA, 68020640 Brazil; 64 Atmospheric and Oceanic Sciences, University of Wisconsin-Madison, Madison, WI 53706 USA; 65 Departamento de Métodos Cuantitativos y Sistemas de Información, Facultad de Agronomía, UBA, Buenos Aires, 1417 Argentina; 66 Department of Earth Sciences, Vrije Universiteit Amsterdam, Amsterdam, 1081 HV The Netherlands; 67 Desertification and Geoecology Department, Experimental Station of Arid Zones, CSIC, Almería, 04120 Spain; 68 School of Life Science, Shanxi University, Taiyuan, 030006 China; 69 HydroFocus, Davis, CA 95618 USA; 70 Institute of BioEconomy, National Research Council of Italy, Sassari, 07100 Italy; 71 Department of Earth, Environment, and Physics, Worcester State University, Worcester, MA 01602 USA; 72 Department of Matter and Energy Fluxes, Global Change Research Institute of the Czech Academy of Sciences, Brno, 60300 Czech Republic; 73 ElObeid Research Station, Agricultural Research Corporation, ElObeid, 51111 Sudan; 74 Airborne Research Australia, TERN Ecosystem Processes Central Node, Parafield, 5106 Australia; 75 Department of Botany, Program in Ecology, University of Wyoming, 1000 E. Univ. Ave, Laramie, WY 82071 USA; 76 Institute of BioEconomy, National Research Council of Italy, Rome, 00100 Italy; 77 Research Centre for Forestry and Wood, Council for Agricultural Research and Economics, Rome, 00166 Italy; 78 Geoscience Australia, Canberra, 2601 Australia; 79 Department of Geosciences and Natural Resource Management, University of Copenhagen, Copenhagen, 1350 Denmark; 80 Energy Analysis & Environmental Impacts Division, Lawrence Berkeley National Laboratory, Berkeley, CA 94720 USA; 81 USDA Forest Service, Rocky Mountain Research Station, Fort Collins, CO 80526 USA; 82 Institute of BioEconomy, National Research Council of Italy, Firenze, 50145 Italy; 83 School of Natural Resources, University of Nebraska-Lincoln, Lincoln, NE 68583 USA; 84 Max Planck Institute for Biogeochemistry, Jena, 03641 Germany; 85 Department of Biology, Virginia Commonwealth University, Richmond, VA 23284 USA; 86 Department of Earth System Science, University of California, Irvine, CA 92697 USA; 87 Agrosphere (IBG3), Forschungszentrum Jülich, Jülich, 52428 Germany; 88 Department of Ecology, University of Innsbruck, Innsbruck, 6020 Austria; 89 International Joint Research Laboratory for Global Change Ecology, School of Life Sciences, Henan University, Kaifeng, 450000 China; 90 Institute of Applied Ecology, Chinese Academy of Sciences, Shenyang, 110016 China; 91 Department of Forest Ecosystems and Society, Oregon State University, Corvallis, OR 97333 USA; 92 College of Resources and Environment, University of Chinese Academy of Sciences, Beijing, 100190 China; 93 School of Ecosystem and Forest Sciences, The University of Melbourne, Creswick, VIC3363 Australia; 94 Research Institute for the Environment and Livelihoods, Charles Darwin University, Darwin, 0909 Australia; 95 Department of Environmental Engineering, Technical University of Denmark (DTU), Kongens Lyngby, 2800 Denmark; 96 Institute for Agro-Environmental Sciences, National Agriculture and Food Research Organization, Tsukuba, 305-8604 Japan; 97 Wageningen Environmental Research, Wageningen University and Research, Wageningen, 6708PB The Netherlands; 98 Key Laboratory of Vegetation Ecology, Ministry of Education, Northeast Normal University, Changchun, 130024 China; 99 Research Faculty of Agriculture, Hokkaido University, Sapporo, 060-8589 Japan; 100 GI-Core, Hokkaido University, Sapporo, 060-0808 Japan; 101 Geography and Environmental Management, Waterloo, ON, N2L3G1 Canada; 102 Institute of Meteorology and Climate Research, Karlsruhe Institute of Technology, Garmisch-Partenkirchen, 82467 Germany; 103 Bioclimatology, University of Goettingen, Goettingen, 37077 Germany; 104 Centre of Biodiversity and Sustainable Land Use (CBL), University of Goettingen, Goettingen, 37077 Germany; 105 Department of Geography, The University of British Columbia, Vancouver, BC, V6T1Z2 Canada; 106 Research Institute for Global Change, Institute of Arctic Climate and Environment Research, Japan Agency for Marine-Earth Science and Technology, Yokoama, 236-0001 Japan; 107 Biological Sciences, University of Adelaide, Adelaide, SA5064 Australia; 108 Graduate School of Agriculture, Kyoto University, Kyoto, 606-8502 Japan; 109 Graduate School of Bioagricultural Sciences, Nagoya University, Nagoya, 4648601 Japan; 110 Department of Applied Physics, University of Granada, Granada, 18071 Spain; 111 Water systems and Global Change group, Wageningen University, Wageningen, 6500 The Netherlands; 112 A.N. Severtsov Institute of Ecology and Evolution, Russian Academy of Sciences, Moscow, 119071 Russia; 113 Head Office, Integrated Carbon Observation System (ICOS ERIC), Helsinki, 00560 Finland; 114 Natural Resources Institute Finland, Helsinki, 00790 Finland; 115 Key Laboratory of Adaptation and Evolution of Plateau Biota, Northwest Institute of Plateau Biology, Chinese Academy of Sciences, Xining, 810008 China; 116 Centre for Tropical Environmental Sustainability Studies, James Cook University, Cairns, 4878 Australia; 117 CEFE, CNRS, Univ Montpellier, Montpellier, 34293 France; 118 Forestry and Environment Division, Forest Research Institute Malaysia (FRIM), Kepong, 52109 Malaysia; 119 Institute for Atmosphere and Earth System Research/Physics, University of Helsinki, Helsinki, 00560 Finland; 120 Department of Botany, School of Natural Sciences, Trinity College Dublin, Dublin, D02PN40 Ireland; 121 German Meteorological Service (DWD), Centre for Agrometeorological Research, Braunschweig, 38116 Germany; 122 Micrometeorology, University of Bayreuth, Bayreuth, 95440 Germany; 123 Bayreuth Center of Ecology and Environmental Research, 95448 Bayreuth, Germany; 124 CSIRO Land and Water, Floreat, 6014 Australia; 125 Department of Agriculture, University of Sassari, Sassari, 07100 Italy; 126 Oulanka research station, University of Oulu, Kuusamo, 93900 Finland; 127 Dept. Biological Sciences, Wellesley College, Wellesley, MA 02481 USA; 128 Research Institute on Terrestrial Ecosystems, National Research Council of Italy, Monterotondo Scalo, 00015 Italy; 129 Department of Geography and Planning, Queen’s University, Kingston, ON, K7L3N6 Canada; 130 Environmental Analytics NZ, Ltd. Raumati South, Paraparaumu, 5032 New Zealand; 131 Mazingira Centre, International Livestock Research Institute (ILRI), Nairobi, 00100 Kenya; 132 NOAA/OAR/Air Resources Laboratory, 325 Broadway, Boulder, CO 80303 USA; 133 Atmospheric Sciences Research Center, State University of New York at Albany, Albany, NY, 12203 USA; 134 Forest Department of South Tyrol, Bolzano, 39100 Italy; 135 Department of Ecology and Evolutionary Biology, University of Arizona, Tucson, AZ 85721 USA; 136 Faculty of Science and Technology, Free University of Bolzano, Bolzano, 39100 Italy; 137 Department of Plant Biology, University of Illinois at Urbana-Champaign, Urbana, IL 61801 USA; 138 IHE Delft, Delft, 2611 The Netherlands; 139 Faculty of Science, VU Amsterdam, Amsterdam, 1081 The Netherlands; 140 University Grenoble Alpes, IRD, CNRS, IGE, Grenoble, 38000 France; 141 School of Engineering and Applied Sciences, Harvard University, Cambridge, MA 02138 USA; 142 Department of Earth and Planetary Sciences, Harvard University, Cambridge, MA 02138 USA; 143 School of Forestry and Resource Conservation, National Taiwan University, Taipei, 0617 Taiwan; 144 International Arctic Research Center, University of Alaska Fairbanks, Fairbanks, AK 99775 USA; 145 Environment and Climate, Research Institute for Nature and Forest, Geraardsbergen, 9500 Belgium; 146 Department of Ecosystem Science and Management, Texas A&M University, College Station, TX 77843 USA; 147 Research Institute for the Environment and Livelihoods, Charles Darwin University, Casuarina, 0810 Australia; 148 Grupo de Estudios Ambientales, Instituto de Matemática Aplicada San Luis (UNSL & CONICET), San Luis, D5700HHW Argentina; 149 Facultad de Ciencias Agropecuarias (UNER), Oro Verde, 3100 Argentina; 150 Eco&Sols, Univ Montpellier-CIRAD-INRA-IRD-Montpellier SupAgro, Montpellier, 34060 France; 151 O’Neill School of Public and Environmental Affairs, Indiana University Bloomington, Bloomington, IN 47405 USA; 152 Global Change Research Group, Dept. Biology, San Diego State University, San Diego, CA 92182 USA; 153 Department of Geography, College of Life and Environmental Sciences, University of Exeter, Exeter, EX44RJ United Kingdom; 154 Department of Agroecology, Aarhus University, Tjele, 8830 Denmark; 155 iCLIMATE, Aarhus University, Tjele, 8830 Denmark; 156 Department of Geology, Wayne State University, Detroit, MI 48202 USA; 157 Department of Geosciences, University of Oslo, Oslo, 0315 Norway; 158 Department of Geography, University of Zurich, Zurich, 8057 Switzerland; 159 Department of Forest Ecology and Management, Swedish University of Agricultural Sciences, Umeå, 90183 Sweden; 160 Hawkesbury Institute for the Environment, Western Sydney University, Penrith, 2751 Australia; 161 Department of Biology, Indiana University Bloomington, Bloomington, IN 47401 USA; 162 Instituto de Clima y Agua, Instituto Nacional de Tecnologia Agropecuaria (INTA), Buenos Aires, 1686 Argentina; 163 CSIRO Land and Water, Wembley, 6913 Australia; 164 Center for Global Change & Earth Observations, Michigan State University, East Lansing, MI 48823 USA; 165 School of Life Science and Engineering, Southwest University of Science and Technology, Mianyang, 621010 China; 166 Departamento de Química e Física, Universidade Federal da Paraiba, Areia, PB, 58397-000 Brazil; 167 Remote Sensing and Geoinformatics, GFZ German Research Centre for Geosciences, Potsdam, 14473 Germany; 168 Andalusian Institute for Earth System Research (CEAMA-IISTA), Granada, 18006 Spain; 169 Ciencias del Agua y Medioambiente, Instituto Tecnológico de Sonora, Ciudad Obregón, 85000 Mexico; 170 Geographical Institute, University of Cologne, Cologne, 50923 Germany; 171 Department of Industry, Innovation and Science, Geoscience Australia, Canberra, 2609 Australia; 172 Southwest Watershed Research Center, USDA-ARS, Tucson, AZ 85719 USA; 173 Institute of Atmospheric Physics of the Czech Academy of Sciences, Prague, 14100 Czech Republic; 174 Department of Ecology, University of Granada, Granada, 18071 Spain; 175 National Hulunber Grassland Ecosystem Observation and Research Station & Institute of Agricultural Resources and Regional Planning, Chinese Academy of Agricultural Sciences, Beijing, 100081 China; 176 School of Science, Edith Cowan University, Joondalup, 6027 Australia; 177 Sentek Pty Ltd, Stepney, SA5069 Australia; 178 National Ecological Observatory Network Program, Boulder, CO 80301 USA; 179 Kansai Research Center, Forestry and Forest Products Research Institute, Kyoto, 612-0855 Japan; 180 College of Urban and Environmental Sciences, Peking University, Beijing, 100871 China; 181 School of Earth, Atmosphere and Environment, Monash University, Clayton, 3800 Australia; 182 Space Science and Engineering Center, University of Wisconsin-Madison, Madison, WI 53706 USA; 183 Terrasystem srl, Viterbo, 01100 Italy; 184 USDA Forest Service, Rocky Mountain Research Station, Missoula, MT 59808 USA; 185 Meteorology and Air Quality group, Wageningen University, 6500 Wageningen, The Netherlands; 186 Fenner School of Environment and Society, Australian National University Canberra, Canberra, ACT 2600 Australia; 187 Department of Civil Engineering, Monash University, Clayton, 3800 Australia; 188 CSIRO Land and Water, Canberra, 2601 Australia; 189 South China Botanical Garden, Chinese Academy of Sciences, Guangzhou, 510650 China; 190 College of Applied Meteorology, Nanjing University of Information Science & Technology, Nanjing, 210044 China; 191 Department of Animal and Plant Sciences, University of Sheffield, Sheffield, S102TN United Kingdom

**Keywords:** Environmental sciences, Climate sciences, Carbon cycle

## Abstract

The FLUXNET2015 dataset provides ecosystem-scale data on CO_2_, water, and energy exchange between the biosphere and the atmosphere, and other meteorological and biological measurements, from 212 sites around the globe (over 1500 site-years, up to and including year 2014). These sites, independently managed and operated, voluntarily contributed their data to create global datasets. Data were quality controlled and processed using uniform methods, to improve consistency and intercomparability across sites. The dataset is already being used in a number of applications, including ecophysiology studies, remote sensing studies, and development of ecosystem and Earth system models. FLUXNET2015 includes derived-data products, such as gap-filled time series, ecosystem respiration and photosynthetic uptake estimates, estimation of uncertainties, and metadata about the measurements, presented for the first time in this paper. In addition, 206 of these sites are for the first time distributed under a Creative Commons (CC-BY 4.0) license. This paper details this enhanced dataset and the processing methods, now made available as open-source codes, making the dataset more accessible, transparent, and reproducible.

## Abstract (summary)

**Table:** 

Measurement(s)	net ecosystem exchange • carbon dioxide • water • energy
Technology Type(s)	eddy covariance • measurement device
Sample Characteristic - Environment	terrestrial biome • atmosphere
Sample Characteristic - Location	Earth (planet)

Machine-accessible metadata file describing the reported data: 10.6084/m9.figshare.12295910

## Background & Summary

For over three decades, the eddy covariance technique^1^ has been used to measure land-atmosphere exchanges of greenhouse gases and energy at sites around the world to study and determine the function and trajectories of both ecosystems and the climate system. The technique allows nondestructive measurement of these fluxes at a high temporal resolution and ecosystem level, making it a unique tool. Based on high frequency (10–20 Hz) measurements of vertical wind velocity and a scalar (CO_2_, H_2_O, temperature, etc.), it provides estimates of the net exchange of the scalar over a source footprint area that extends up to hundreds of meters around the point of measurement. Soon after the first consistent measurement sites were operational, regional networks of sites were formed in Europe^2^ and the US^3,4^, followed by similar initiatives in other continents^5–7^. Networks enabled the use of eddy covariance data beyond a single site or ecosystem for cross-site comparisons and regional-to-global studies^8–15^. These regional networks have evolved into long-term research infrastructures or monitoring activities, such as ICOS, AmeriFlux, NEON, AsiaFlux, ChinaFLUX, and TERN-OzFlux.

FLUXNET was created as a global network of networks^16–18^, a joint effort among regional networks to harmonize and increase standardization of the data being collected. It made possible the creation of global eddy covariance datasets. The first gap-filled, global FLUXNET dataset, which included derived, partitioned fluxes like photosynthesis and respiration, was the Marconi dataset^19^ in 2000, with 97 site-years of data, followed by the 2007 LaThuile dataset^20^ with 965 site-years of data, and finally in 2015 the FLUXNET2015 dataset^18,21^ (hereafter FLUXNET2015) with 1532 site-years of data. Two main factors limited the numbers of sites and years included in each dataset: data policy and data quality. Willingness to share data under the selected data policy is a major reason why FLUXNET2015 likely only includes between 10–20% of existing sites globally–the total number of existing sites is still unknown. Then there is the evolution of processing pipelines and quality controls, leading to new issues being identified in the data that, if not solved in time, led to leaving out that data. The LaThuile dataset had a more restrictive policy and, in a few cases, previously undiscovered data issues, leading to fewer sites being included in FLUXNET2015.

**Fig. 1:**
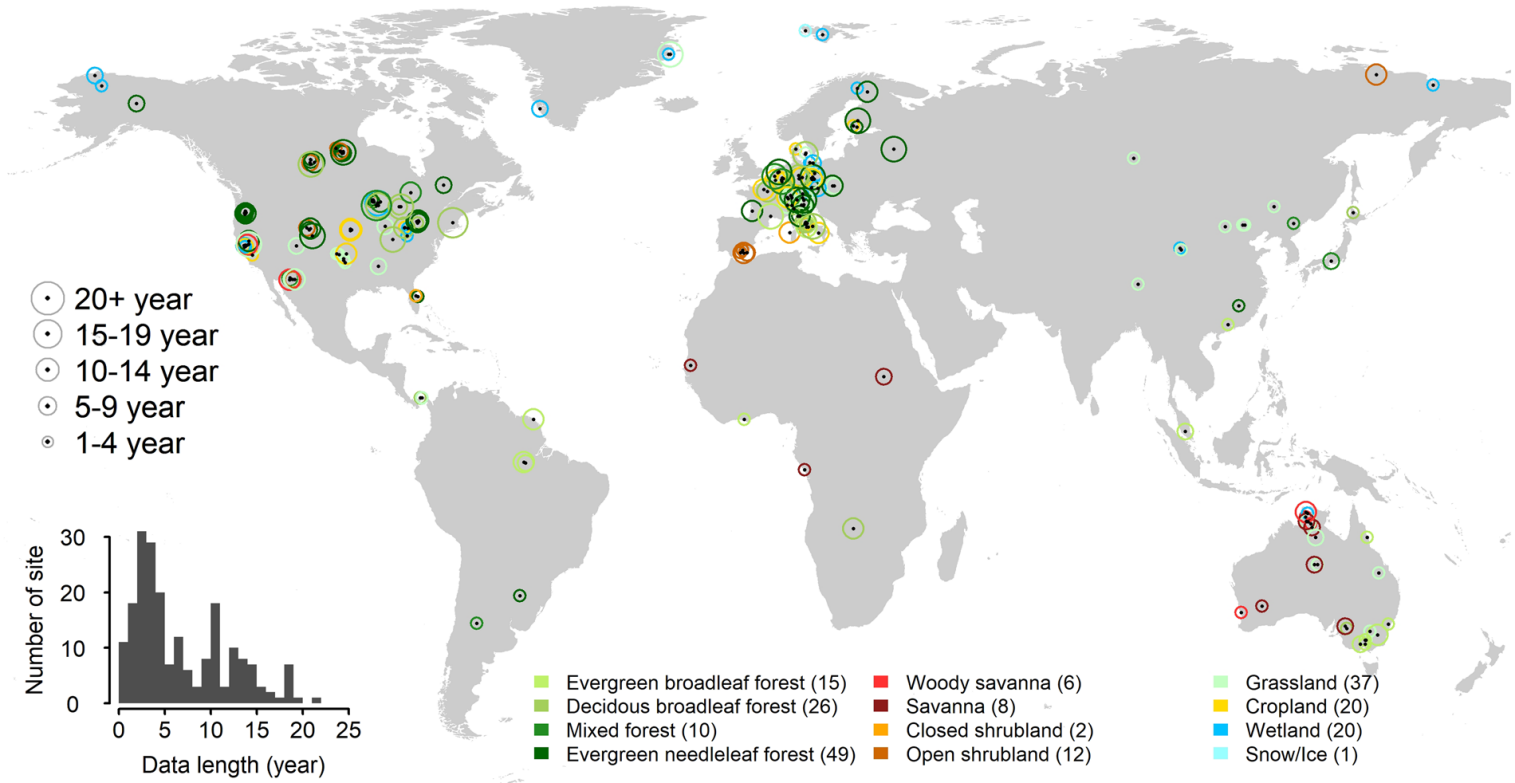
Map of 206 tower sites included in this paper from the 212 sites in the February 2020 release of the FLUXNET2015 dataset. The size of the circle indicates the length of the data record. The color of the circles represents the ecosystem type based on the International Geosphere–Biosphere Programme (IGBP) definition. When overlapping, locations are offset slightly to improve readability. Numbers in parentheses indicate the number of sites in each IGBP group. The inset shows the distribution of data record lengths. See also Supplementary Fig. SM4 for continental scale maps of Australia, Europe, and North America.

FLUXNET2015 includes for the first time sites with records over two decades long (Fig. 1). The dataset was created through collaborations among many regional networks, with data preparation efforts happening at site, regional network, and global network levels. The global coordination of data preparation activities and data processing was done by a team from the AmeriFlux Management Project (AMP), the European Ecosystem Fluxes Database, and the ICOS Ecosystem Thematic Centre (ICOS-ETC). This team was responsible for the coding efforts, quality checks, and execution of the data processing pipeline. These combined efforts led to a dataset that is standardized with respect to the (1) data products themselves, (2) data distribution formatting, and (3) data quality across sites. The wide application of these datasets in global synthesis and modeling activities highlights their value. At the same time, however, heterogeneity in the data–caused mainly by differences in data collection, flux calculations, and data curation before submission–highlights the need for estimates of data uncertainty and uniform evaluation of data quality.

The data processing pipeline uses well-established and published methods, with new code implemented for this release as well as code adapted from implementations by the community. The main products in this pipeline are: (1) thorough data quality control checks; (2) calculation of a range of friction velocity thresholds to filter low turbulence periods, allowing an estimate of the uncertainty from this filtering along with the random uncertainty; (3) gap-filling of meteorological and flux measurements, including the use of a downscaled reanalysis data product to fill long gaps in meteorological variables; (4) partitioning of CO_2_ fluxes into respiration and photosynthesis (gross primary productivity) components using three distinct methods; and (5) calculation of a correction factor for energy fluxes estimating the deviation from energy balance closure for the site. Two features of this pipeline are the ranges of friction velocity thresholds, and the multiple methods for partitioning CO_2_ fluxes. Both features support a more thorough evaluation of the uncertainty introduced by the processing steps themselves. Our implementation of this pipeline is available as an open-source code package called ONEFlux (Open Network-Enabled Flux processing pipeline)^22^. The goal of this paper is to describe FLUXNET2015 and additional products, present the details about this processing pipeline, and document the methods used to generate the dataset. Doing so will provide the community of FLUXNET end-users with the technical and practical knowledge necessary to harness the full potential of the FLUXNET data, including data from the FLUXNET2015 release, and data submitted to the network since.

## Data Processing Methods

**Fig. 2:**
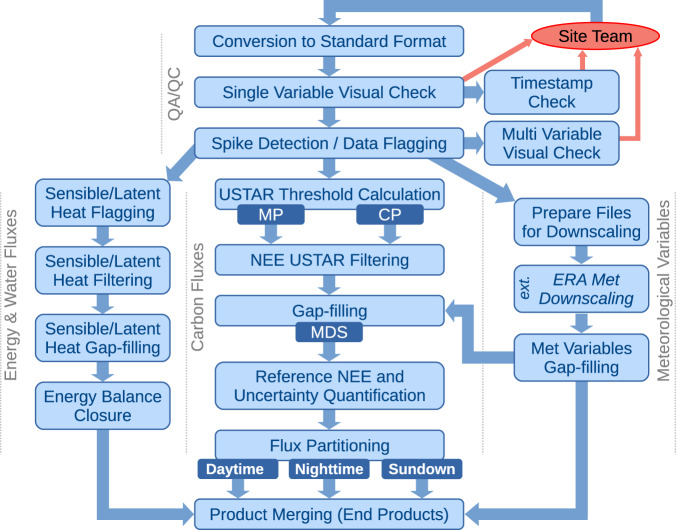
The logic of the data processing steps for FLUXNET2015 (details about the different steps and meaning of abbreviations in the text).

The data contributed by site teams for inclusion in FLUXNET2015 encompassed fluxes, meteorological, environmental, and soil time series at half-hourly or hourly resolutions. Contributed data underwent a uniform data quality control process, with issues addressed in consultation with site teams. Data were then processed using the pipeline (Fig. 2) described in this section. The resulting data products were distributed through the FLUXNET-Fluxdata web portal^23^, where the usage of the dataset is tracked through a registration of all the requests and details about the user and the data use plan. This information is crucial to better understand the user needs and the impact of the dataset.

### Data sources

The first level of processing was completed by the site teams, including the calculation of half-hourly or hourly turbulent fluxes from high-frequency wind and concentration measurements, the averaging of meteorological variables sampled at shorter temporal resolutions, and the site team’s own quality control procedures. The contributed fluxes were required to be submitted separately as turbulent and storage fluxes (components to be added for total flux), and not gap-filled or filtered for low turbulence conditions–see Aubinet *et al*.^24^ for details. Control checks were implemented to ensure that this was the case, and to detect additional inconsistencies that site teams were asked to address, for example coherence of the timestamps, consistency across correlated variables, etc^25^. Starting from these data, we applied the same set of methods for all the remaining processing steps (from filtering to gap-filling and partitioning), increasing uniformity, and allowing quantification of the uncertainty introduced by the processing. The harmonization of data–especially of data quality–was a high priority while creating the dataset, and therefore extensive interaction with the site teams was necessary. Data were contributed through a regional network, and the formats provided by the networks were converted into a standard input for processing. This FLUXNET dataset led to the creation of a new cross-network specification for standard site data and metadata submission formats, now adopted by different regional networks.

### Data processing pipeline overview

The data processing pipeline (Fig. 2) is divided into four main processing blocks. The first one is the data quality assurance and quality control (QA/QC) activities our team applied to data from all sites. This part was done with a combination of automated procedures and manual checks for all the variables in the contributed data. The next three blocks were all part of an automated pipeline that was executed separately for each site: the Energy & Water Fluxes processing block encompasses sensible and latent heat variables; the Carbon Fluxes processing block handles the variables for CO_2_ fluxes like net ecosystem exchange (NEE); and, the Meteorological Variables processing block deals with all the meteorological measurements that are also used in the processing of fluxes and other products. At each processing step, a set of automated pre- and post-conditions are enforced, making sure the inputs and outputs of each step are within the expected behavior. The final step involved merging all the products generated at previous steps, and adding daily through yearly temporal aggregations of most of the variables in the dataset and related quality flags. At this step, automated checks were performed on all the variables to ensure consistency, and the final files with all the contributed and derived data products to be distributed are created. Supplementary Fig. SM2 shows the general steps involved in the processing in the sequence organized in the code, as available in the ONEFlux package^22^. All the steps have been implemented in the shared code except the Sundown partitioning (see Implementation Approach section for details).

### Ensemble of results

The adoption of multiple methods for the same step (e.g., two methods for USTAR threshold calculation, or two or three methods for CO_2_ flux partitioning, see below in this section for details) is motivated by the existence of different methods in the literature, using different assumptions and potentially having diverging results^26,27^. On the one hand, this lack of uniformity can represent a problem for synthesis studies. On the other hand, adopting a single method could lead to biases and underestimation of the uncertainty in the methodology. The approach taken here, which simultaneously adopts multiple methods, allows the creation of an ensemble, helping assess uncertainty, and also the suitability of individual methods to a site’s conditions.

### Data quality assurance and quality control

Prior to the processing that generated the derived data products (hereafter called *post-processing*), the data for each site went through QA/QC checks following Pastorello *et al*.^25^. All variables included in the dataset underwent checks, and critical variables underwent further scrutiny. These additional checks targeted variables critical to the processing, e.g., flux variables, meteorological variables used in gap-filling, and variables used by uncertainty estimation procedures. The processing did not proceed for sites with pending issues in critical variables.

**Critical metadata variables for the post-processing:**

- The site FluxID in the form CC-SSS (two character country code, three character site identifier within country) – e.g., US-Ha1
- The latitude and longitude for the site in the WGS 84 decimal format with at least four decimal points resolution – e.g., 42.5378/−72.1715
- Time zone of the site (time series, if time zone changed; timestamps are all local standard time, no daylight savings) – e.g., UTC-5
- Height of the gas analyzer – e.g., 30.0 m

Critical data variables for the post-processing, averaged or integrated over 30 or 60 minutes (*required):

*CO_**2**_ (µmolCO_2_ mol^−1^): Carbon Dioxide (CO_2_) mole fraction in moist air
*FC (µmolCO_2_ m^−2^ s^−1^): Carbon Dioxide (CO_2_) turbulent flux (without storage component)
*SC (µmolCO_2_ m^−2^ s^−1^): Carbon Dioxide (CO_2_) storage flux measured with a vertical profile system, optional if tower shorter than 3 m
*H (W m^−2^): sensible heat turbulent flux, without storage correction
*LE (W m^−2^): latent heat turbulent flux, without storage correction
*WS (m s^−1^): horizontal wind speed
*USTAR (m s^−1^): friction velocity
*TA (deg C): air temperature
*RH (%): relative humidity (range 0–100%)
*PA (kPa): atmospheric pressure
- **G** (W m^−2^): ground heat flux, not mandatory, but needed for the energy balance closure calculations
- **NETRAD** (W m^−2^): net radiation, not mandatory, but needed for the energy balance closure calculations
*SW_IN (W m^−2^): incoming shortwave radiation
- **SW_IN_POT** (W m^−2^): potential incoming shortwave radiation (top of atmosphere theoretical maximum radiation), calculated based on the site coordinates^22^
- **PPFD_IN** (µmolPhotons m^−2^ s^−1^): incoming photosynthetic photon flux density
- **P** (mm): precipitation total of each 30 or 60 minute period
- **LW_IN** (W m^−2^): incoming (down-welling) longwave radiation
- **SWC** (%): soil water content (volumetric), range 0–100%
- **TS** (deg C): soil temperature

#### File format standardization

To process hundreds of sites, we needed consistent file formats that supported the input data and metadata. This led to multi-network agreements and creation of formats for data and metadata contribution to the regional networks^28,29^. These formats have now been adopted by networks in Europe and the Americas and by some instrument manufacturers, and are under consideration by other regional networks. In addition, automated extraction and conversion tools for direct format translation were implemented to work with data in older formats.

#### Data QA/QC steps

Data quality was checked before the processing started. If issues were identified that could not be resolved by the network-level data team, the site team was asked to suggest a course of action or send a new version of the data addressing the quality issue identified. The main data QA/QC steps were: single-variable checks, multi-variable checks, specialized checks, and automatic checks. Single-variable checks look at patterns in the time series of one variable at a time, for long- and short-term trends and other issues. Multi-variable checks look at the relationships among correlated variables (e.g., different radiation variables) to identify periods with disagreements. Specialized checks test for common issues in EC and meteorological data, like timestamp shifts or sensor deterioration. During this phase, a time series of top-of-the-atmosphere potential radiation (SW_IN_POT) is also computed, using latitude/longitude coordinates and time^22^. These three types of checks are detailed in Pastorello *et al*.^25^. The automated checks apply variable-specific despiking routines adapted from Papale *et al*.^30^ and apply a set of range controls per variable. This last step creates a series of flags that were discussed with the site managers for corrections and resubmissions and then used to filter the data in subsequent steps.

### Meteorological products

The main processing applied to meteorological data was gap-filling by two independent methods: Marginal Distribution Sampling^31^ (MDS) and ERA-Interim^32^. Data gap-filled by MDS (applied to all variables that are gap-filled) are identified by the _F_MDS suffix. Data gap-filled using ERA-Interim downscaling (six variables that are available in the reanalysis dataset) have a _F_ERA suffix. The final gap-filled time series for variables combines both of these methods (indicated by an unqualified _F suffix), following a data-quality-based selection approach (see below). For SW_IN, in case of gaps or in case the variable was not measured, we performed a calculation from PPFD_IN when available, calculating the conversion factor from the periods of overlap of the two measurements (and assuming a factor 0.48 J (µmol photon)^−1^ when the sensors did not run in parallel at the site).

#### MDS

The MDS method, introduced in Reichstein *et al*.^31^, is applied to all variables that may be gap-filled. It works by seeking meteorological conditions physically and temporally similar to the ones for the missing data point(s). The restrictions on the size of the time window and which variables must be available are incrementally relaxed until a suitable set of records is found to fill the gap in the target variable. All values of the target variable satisfying the current set of conditions are averaged to generate the fill value. The method was applied as described in the original implementation^31^, using SW_IN, TA and VPD as drivers. The basic three scenarios for the time when the target variable is missing are: (i) all three drivers are available; (ii) only SW_IN is available; and (iii) all three drivers are missing. Based on the available co-located variables, a search for similar conditions is started, keeping the searching window as small as possible to avoid changes in other slow-changing drivers (phenology, water availability, etc.). The more variables missing and the larger the time window, the lower the confidence indicated by the _F_MDS_QC flag. The values for this flag are (0–3): _F_MDS_QC = 0 (measured); _F_MDS_QC = 1 (filled with high confidence); _F_MDS_QC = 2 (filled with medium confidence); _F_MDS_QC = 3 (filled with low confidence). For details on the implementation, see the original paper^31^ and the ONEFlux source code^22^.

#### ERA-Interim

This method is based on the ERA-Interim (ERA-I) Reanalysis global atmospheric product^33,34^ created by the European Centre for Medium‐Range Weather Forecasts (ECMWF)–ERA stands for ECMWF Re-Analysis. Applied to the subset of variables that are also available in the ERA-Interim product, the method involves a spatial and temporal downscaling process using the measured variable at the site. The ERA-I variables that were used are: air temperature at 2 m (t2m, K), incoming shortwave solar radiation at the surface (Sw, W m^−2^), dew point temperature at 2 m (dt2m, K), wind speed horizontal components at 10 m (u10 and v10, m s^−1^), total precipitation (Pr, m of water per time step), and incoming longwave solar radiation at the surface (Lw, W m^−2^). The gap-filling procedure harmonizes units, identifies periods that are long enough to allow a linear relationship to be built, a simple debiasing of the linear relationship, evaluation of the diurnal cycle in the subset of variables, and other evaluations of the results to identify potential missing or incorrect information (e.g., coordinate or temporal mismatches). The linear relationships are built taking into account instantaneous and averaged variables, and then applied to the whole ERA-I record, generating the spatially (coordinate-based) and temporally (diurnal cycle-based) downscaled version of each variable. The method was applied as in the original implementation; additional details can be found in Vuichard and Papale^32^.

#### Final gap-filled product

Measured or high quality gap-filled records using MDS (_F_MDS_QC < 2) are used in the final gap-filled products (_F suffixed variables, without _MDS or _ERA). If the variable has a low quality gap-fill flag (2 or 3), the ERA-I product is used instead. The final quality flag (_F_QC) is 0 for measured, 1 for high quality fill using MDS, and 2 for data gap-filled with the ERA-I downscaled product. A gap-filled version of CO_2_ concentration is also generated (CO_2__F_MDS) using the MDS method as described above, including the corresponding quality flag.

### Energy and water products

The main data products associated with energy and water fluxes are the gap-filled versions of the data and the estimation of a version ensuring the energy balance closure and estimating its uncertainty–for a description of the issue see Stoy *et al*.^35^ Turbulent energy fluxes (sensible and latent heat, H and LE, respectively) are gap-filled using the MDS method^31^ described above. From LE, it is possible to calculate the water flux (evapotranspiration) using the latent heat of vaporization. An energy balance corrected version of LE and H is also created, a data product often needed when data are used in model parameterization and validation for which the closure of the energy balance is prescribed. There is no general agreement on the reasons and approaches to correct the imbalance in the energy budget within EC measurements. In this product, the methodology used to calculate the energy balance corrected fluxes is based on the assumption that the Bowen ratio is correct^36^. Fluxes are corrected by multiplying the original, gap-filled LE and H data by an energy balance closure correction factor (EBC_CF, in the dataset). The correction factor is calculated starting from the half-hours where all the variables needed to estimate energy balance closure are available (measured NETRAD and G, and measured or good-quality gap-filled H and LE). The correction factor for each single half-hour is calculated as in Eq. (1), but is not applied directly.

1 $$EBC\_CF=(NETRAD-G)/(H+LE)$$

First, to avoid transient conditions, the calculated EBC_CF time series is filtered by removing values outside of 1.5 times its own interquartile range. Then, the correction factor used in the calculations is obtained using one of three methods, applied hierarchically (see also diagram in Supplementary Fig. SM3):

- **EBC_CF Method 1:** For each half-hour, a sliding window of ±15 days (31 days total) is used to select half-hours between time periods 22:00–02:30 and 10:00–14:30 (local standard time). These time-of-day restrictions aim at removing sunrise and sunset time periods, when changes in ecosystem heat storage (not measured) are more significant, preventing energy balance closures. For all half-hours meeting these criteria, the corresponding EBC_CFs are selected and used to calculate the corrected values of H and LE for the half-hour processed (center of the sliding window), generating a pool of values for each of these two variables. From each of these two pools, the 25th, 50th (median), and 75th percentiles are extracted for their corresponding variables, generating the values for H_CORR25, H_CORR, H_CORR75 and LE_CORR25, LE_CORR, LE_CORR75. If fewer than five EBC_CF values are present in the sliding window, Method 2 is used for the half-hour. (*Note on temporal aggregations: for DD the sliding window size is* ±*7 days and the EBC_CF are calculated from the daily average values of G, NETRAD, H and LE. For WW, MM, and YY the EBC_CFs are calculated from corresponding average fluxes of the period analysed, but no percentiles are computed. For WW, MM, and YY, Method 1 fails if less than 50% of half-hours within the window have measured values for all four component variables*.)
- **EBC_CF Method 2:** For the current half-hour, EBC_CF is calculated as the average of the EBC_CF values used to calculate the H_CORR and LE_CORR with Method 1 within a sliding window of ±5 days and ±1 hour of the time-of-day of the current timestamp. H_CORR and LE_CORR are calculated and the corresponding _CORR25 and _CORR75 percentiles are not generated. If no EBC_CF is available, Method 3 is used for the current half-hour. (*Note on temporal aggregations: differing sliding windows are: DD*: ±*2 weeks, WW:* ±*2 weeks, MM*: ±*1 month, and YY*: ±*1 year.)*
- **EBC_CF Method 3:** An approach like Method 2 is applied but using a sliding window of ±5 days for the same half-hour in the previous and next years, with the current EBC_CF being calculated from the average of the EBC_CF values used to calculate the H_CORR and LE_CORR. H_CORR and LE_CORR are calculated and the corresponding _CORR25 and _CORR75 percentiles are not generated. In case this method also cannot be applied due to missing values, the energy balance closure corrected fluxes are not calculated. (*Note on temporal aggregations: differing sliding windows are: DD:* ±*2 weeks, WW:* ±*2 weeks, MM:* ±*1 month, and YY:* ±*2 years*.)

#### H and LE Random Uncertainty

The random uncertainty for H and LE is also estimated at half-hourly resolution, based on the method introduced by Hollinger & Richardson^37^ and then aggregated at the other temporal resolutions. The random uncertainty (indicated by the suffix _RANDUNC) in the measurements is estimated using one of two methods, applied hierarchically:

- **H-LE-RANDUNC Method 1 (direct standard deviation method):** For a sliding window of ±5 days and ±1 hour of the time-of-day of the current timestamp, the random uncertainty is calculated as the standard deviation of the measured fluxes. The similarity in the meteorological conditions evaluated as in the MDS gap-filling method^31^ and a minimum of five measured values must be present; otherwise, method 2 is used.
- **H-LE-RANDUNC Method 2 (median standard deviation method):** For the same sliding window of ±5 days and ±1 hour of the time-of-day of the current timestamp, random uncertainty is calculated as the median of the random uncertainty (calculated with H-LE-RANDUNC Method 1) of similar fluxes, i.e., within the range of ±20% and not less than 10 W m^–2^.

The joint uncertainty for H and LE is computed from the combination of the uncertainty from the energy balance closure correction factor and random uncertainty.

2 $$H\_CORR\_JOINTUNC=\sqrt{H\_RANDUN{C}^{2}+{\left(\frac{H\_CORR75-H\_CORR25}{1.349}\right)}^{2}}$$

These variables are identified by the _JOINTUNC suffix and are computed for H as in Eq. (2), and similarly for LE. (*Note on temporal aggregations: joint uncertainties for H and LE are recomputed at HH and DD resolutions separately, and not generated for WW, MM, and YY resolutions*.)

### CO_2_ products

The processing steps applied to CO_2_ fluxes were: calculation of net ecosystem exchange (NEE) from CO_2_ turbulent and storage fluxes, applying a spike detection algorithm, filtering for low turbulence conditions using multiple friction velocity (USTAR) thresholds, gap-filling of all NEE time series generated by an ensemble of USTAR thresholds, estimation of random uncertainty, and partitioning of NEE into its ecosystem respiration (RECO) and gross primary production (GPP) components.

#### Calculation of NEE

CO_2_ storage fluxes (SC) express the change of CO_2_ concentration below the measurement level of the eddy covariance system within the half-hour. NEE was calculated as the sum of the CO_2_ turbulent fluxes (FC) and SC. Both FC and SC are part of the required data contributed by site teams. SC is usually estimated using a profile system^38^. If SC was not provided or missing, two cases were implemented: for measurement heights lower than 3 m and short canopies, the SC term was considered to be 0; for taller towers/canopies, a discrete estimation based on the top measurement of CO_2_ concentration was used to compute SC^39^.

#### Despiking of NEE

Although the processing of high frequency data into half-hourly fluxes usually includes steps to remove spikes from instantaneous measurements, spikes can also occur in the half-hourly data. The method described in Papale *et al*.^30^, based on the median absolute deviation (MAD) with z = 5.5, was applied to filter NEE for residual spikes that were removed.

#### USTAR threshold estimation and filtering

Filtering for low turbulence conditions is necessary when there is not enough turbulence, causing the ecosystem flux to be transported by advective flows and missed by both the eddy covariance system and the storage profile, resulting in underestimated fluxes. Despite different approaches having been tested to measure and quantify horizontal and vertical advection^40^, the most often used method to avoid the underestimation of fluxes is removing the data points potentially affected by strong advection^1^. These points are identified using the friction velocity (USTAR) as an indicator of turbulence strength, defining a threshold value under which NEE measurements are discarded and replaced by gap-filled estimates.

This USTAR threshold is linked to the canopy structure, measurement height, wind regimes, and other factors specific to an individual site. It is estimated using nighttime NEE measurements (only ecosystem respiration), based on the dependency between USTAR and NEE at similar temperatures and periods of the year (main drivers of ecosystem respiration). Under these conditions, NEE is assumed (and expected) to be independent from USTAR, which is not a driver of respiration. However, in most sites below a certain USTAR threshold value, NEE is found to increase with USTAR; this USTAR value is selected as the threshold to define conditions with reduced risk of flux underestimation. Different methods have been proposed to estimate the USTAR thresholds and the related uncertainty as to how the approach works at a specific site^1^.

#### CP and MP USTAR threshold methods

Two methods to calculate USTAR thresholds were used: change-point-detection (CP) proposed by Barr *et al*.^41^ and a modified version of the moving-point-transition (MP) described originally by Reichstein *et al*.^31^ and Papale *et al*.^30^. Both methods are similar in terms of data selection, preparation and grouping and aim to estimate the USTAR threshold value. Measurements collected when USTAR is below the threshold are removed. The difference between these methods is in how this threshold value is estimated. For both methods, the nighttime data of a full year are divided in four three-month periods (seasons) and 7 temperature classes (of equal size in terms of number of observations). For each season/temperature group the data are divided into 20 USTAR classes (also with equal number of observations) and the average NEE for each USTAR class is computed. The calculation of the threshold uses each of the methods (see below for details on their differences). For each season, the median value of the 7 temperature classes is calculated and a final threshold is defined by selecting the maximum of the 4 seasonal values.

The CP method uses two linear regressions between NEE and USTAR, the second with an imposed zero slope. The change point is defined as where the two lines cross, i.e., constraining the shape of the NEE-USTAR dependency. The method is extensively used to detect temporal discontinuities in climatic data. Details can be found in Barr *et al*.^41^.

For the MP method^30,31^, the mean NEE value in each of the 20 USTAR classes is compared to the mean NEE measured in the 10 higher USTAR classes. The threshold selected is the USTAR class in which the average nighttime NEE reaches more than 99% of the average NEE at the higher USTAR classes. An improvement of the MP method was implemented here for robustness over noisy data, by adding a second step to the original MP implementation: when a threshold is selected, it was tested to ensure it was also valid for the following USTAR class. In other words, assuming that Eq. (3) holds, where *x* is one of the 20 USTAR classes and $NEE{\rm{\_}}USTAR(x)$ is the average NEE for that USTAR class.

3 $$\begin{array}{c}NEE{\rm{\_}}USTAR(x) > 0.99\,\times MEAN(NEE{\rm{\_}}USTAR(x+1),\\NEE{\rm{\_}}USTAR(x+2),\,...,\,NEE{\rm{\_}}USTAR(x+10))\end{array}$$

The USTAR value associated to the *x*^*th*^-class was selected as threshold only if Eq. (4) also holds, to confirm that the plateau where NEE is USTAR-independent was reached. If not, the search for the plateau and threshold continued toward higher USTAR values.

4 $$\begin{array}{c}NEE{\rm{\_}}USTAR(x+1) > 0.99\,\times MEAN(NEE{\rm{\_}}USTAR(x+2),\\NEE{\rm{\_}}USTAR(x+3),\,\ldots ,\,NEE{\rm{\_}}USTAR(x+11))\end{array}$$

#### Bootstrapping USTAR threshold estimation

**Fig. 3:**
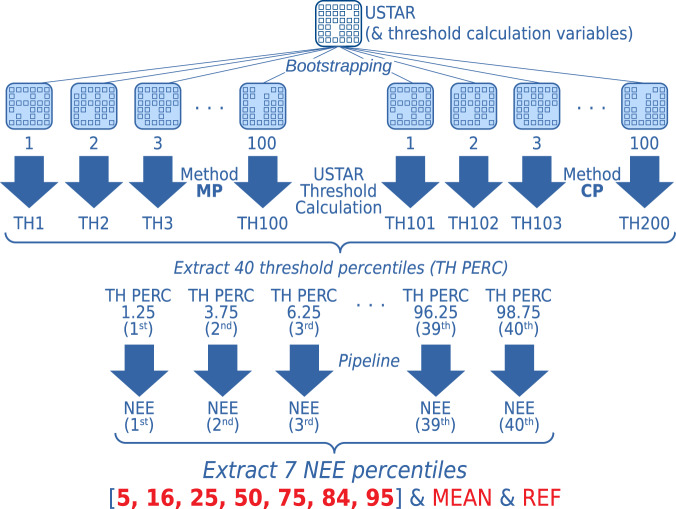
To identify and remove data collected under low turbulence conditions, under which advective fluxes could lead to an underestimation of fluxes, filtering based on the USTAR threshold was used. In order to estimate the uncertainty in the USTAR threshold calculation, a bootstrapping approach was implemented, with a selection of values representative of the distribution included in the final data products. From the (up to) 200 thresholds from the combined bootstrapping of the two methods, 40 percentiles are extracted. All the subsequent steps of the pipeline are applied to all 40 versions. For each of the final output products (e.g., NEE, as illustrated here), seven percentiles representative of the distribution are included.

For each of the two methods, a bootstrapping technique was used. The full dataset (year of measurement) was re-sampled 100 times with the possibility to select the same data point multiple times (i.e., with replacement), creating 100 versions of the dataset. The threshold values were calculated for each of them, obtaining 100 threshold values per method (CP and MP) and year, for a total of 200 USTAR threshold estimates for each year. This process and next steps are illustrated in Fig. 3. These 200 threshold values represent the uncertainty in the threshold estimation that could also impact the uncertainty of NEE. It is worth noting that there is not always a direct relationship between the threshold and NEE uncertainties. It is possible, for instance, that a small variability in the thresholds has a strong effect on NEE or, conversely, with NEE almost insensitive to the threshold value. This is related to the site characteristics (USTAR variability) and to the level of difficulty in filling the gaps created by the filtering.

There are cases where not enough data are present to calculate a USTAR threshold (for both the CP and MP methods) or where it is not possible to identify a clear change point (CP method only). This leads to the uncertainty being underestimated (fewer or no USTAR threshold values available). This should be considered as a general indication of difficulties in the application of the USTAR filtering for the specific sites or years. Sites and years where these conditions occurred are reported in the SUCCESS_RUN variable in the AUXNEE product (values 1: threshold found, 0: failed/no threshold found).

#### Variable and constant variants of the USTAR threshold methods

To calculate the uncertainty in NEE due to the uncertainty in the selected USTAR threshold, all the threshold values obtained with the two methods and the bootstrapping were pooled together, from which 40 representative values were extracted: from the percentile 1.25 of the series to the percentile 98.75, with a step of 2.5, i.e., [1.25:2.5:98.75]. When long time series (multi-years) are processed, it is possible to extract the 40 representative thresholds for each of the years. The threshold is a function of slow-changing dynamics (height of canopy, height of measurement, roughness), but a threshold changing every year could introduce false interannual variability. On the other hand, a constant threshold across all the years would not represent changes in the ecosystem structure and EC system setup. For this reason, two approaches were implemented:

- Variable USTAR Threshold (VUT): The thresholds found for each year and the years immediately before and after (if available) have been pooled together, and from their joint population, the final 40 thresholds extracted. With that, the USTAR thresholds vary from year to year; however, they are still influenced by neighboring years. This is identified in FLUXNET2015 variables by the “_VUT” suffix;
- Constant USTAR Threshold (CUT): Across years, all the thresholds found have been pooled together and the final 40 thresholds extracted from this dataset. With that, all years were filtered with the same USTAR threshold. This is identified in FLUXNET2015 variables by the “_CUT” suffix.

If the dataset includes up to two years of data, the two methods give the same result, and only the _VUT is generated.

**Fig. 4:**
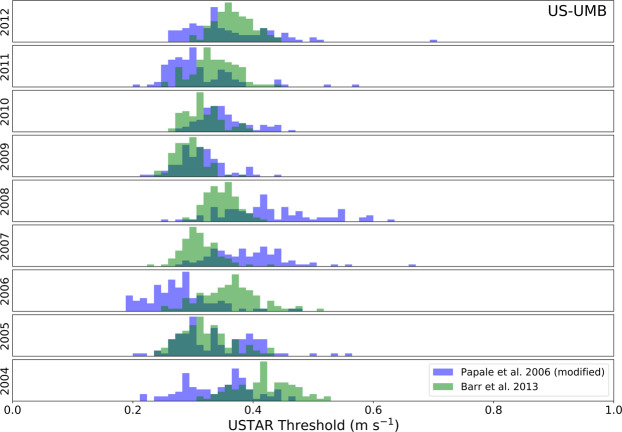
Example of the distribution of USTAR thresholds calculated for each year using the MP^30^ method in blue and CP^41^ method in green for the US-UMB site (dark green where they overlap). All these thresholds were pulled together to extract the CUT final 40 thresholds, while for the VUT thresholds, each year was pulled with the two immediately before and after (e.g., 2005 + 2006 + 2007 to extract the 40 thresholds to be used to filter 2006). Note that the level of agreement between methods and between subsequent years is variable, justifying the approach that propagates this variability into uncertainty in NEE.

For both the VUT and CUT approaches, 40 NEE datasets have been created, filtering the original NEE time series using 40 different USTAR values estimated as explained above. The values of the thresholds are reported in the AUXNEE product file. These 40 NEE versions have been used as the basis for all the derived variables provided. An example of the variability of the two methods (CP and MP) is shown in Fig. 4, contrasting the distribution of the bootstrapped results for each method, showing comparable values for some years and divergent values for other years (of the same site). This highlights the importance of applying both methods in this ensemble-like way.

#### Filtering NEE based on USTAR thresholds

**Fig. 5:**
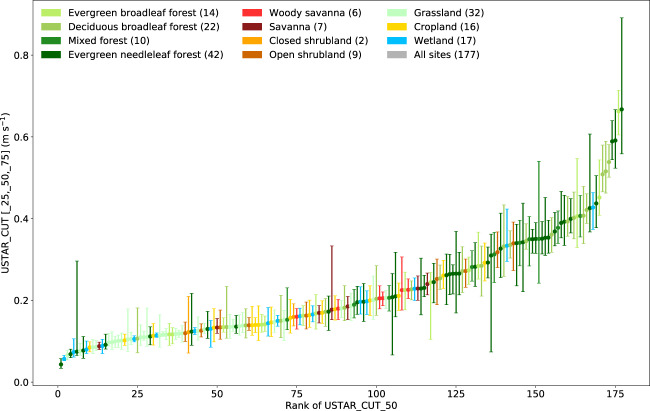
Ranked USTAR thresholds based on median threshold and error bars showing 25^th^ to 75^th^ percentiles of the 40 thresholds calculated with the Constant USTAR Threshold (CUT) method – only computed for sites with 3 or more years, so only 177 sites out of the 206 are shown. Colors show different ecosystem classes based on the site’s IGBP.

The USTAR thresholds are applied to daytime and nighttime data, removing NEE values collected when USTAR is below the threshold and removing also the first half-hour with high turbulence after a period of low turbulence to avoid false emission pulses due to CO_2_ accumulated under the canopy and not detected by the storage system (in particular, when a profile is not available at the site). The USTAR filtering is not applied to H and LE, because it has not been proved that when there are CO_2_ advective fluxes, these also impact energy fluxes, specifically due to the fact that when advection is in general large (nighttime), energy fluxes are small. Figure 5 shows the range of thresholds found (interquartile ranges) across sites in FLUXNET2015. While some sites had low thresholds and low variability in the USTAR thresholds, others show large ranges of values in some more extreme cases (indicating difficulties in estimating the “real” threshold).

#### Gap-filling of NEE

Existing gaps from instrument or power failures are further increased after QC and USTAR filtering. The time series with gaps need to be filled, especially before aggregated values can be calculated (from daily to annual). Moffat *et al*.^26^ compared different gap-filling methods for CO_2_ concluding that most of the methods currently available perform sufficiently well with respect to the general uncertainty associated with the measurements. The method implemented here is the Marginal Distribution Sampling (MDS) method already described in the meteorological products.

#### Selection of reference NEE variables

After filtering NEE using the 40 USTAR thresholds and gap-filling, 40 complete (gap-free) NEE time series were available for each site. For each half hour, it is possible to use the 40 values to estimate the NEE uncertainty resulting from the USTAR threshold estimation (reported as percentiles of the NEE distribution, identified by the “_XX” numeric suffix) and the average value (identified in the dataset by the “_MEAN” suffix). Since the average value has a smoothing effect on the time series, an additional reference value of NEE was selected and identified in FLUXNET2015 variables by the “_REF” suffix, in an attempt to identify which of the 40 NEE realizations was the most representative of the ensemble. The “_REF” NEE was selected among the 40 different NEE instances in this way: (1) the Nash–Sutcliffe model efficiency coefficient^42^ was calculated between each NEE instance and the remaining 39; (2) the reference NEE has been selected as the one with the highest model efficiency coefficients sum, i.e., the most similar to the other 39. Note that determining the reference NEE is done independently for variables using VUT and CUT USTAR thresholds, as well as for each temporal aggregation. Therefore, the version selected as REF could be different for different temporal resolutions. For instance, NEE_VUT_REF at half-hourly resolution might have been generated using a different USTAR threshold than NEE_VUT_REF at daily resolution. Information on which threshold values were used for each version and temporal aggregation can be found in the auxiliary products for NEE processing (AUXNEE). In addition to the reference NEE, the NEE instance obtained by filtering the data with the median value of the USTAR thresholds distribution is also included. This NEE is identified in FLUXNET2015 variables by the “_USTAR50” suffix (for both CP and MP methods, and both VUT and CUT approaches) and is stable across temporal aggregation resolutions. Individual percentiles of the USTAR thresholds distribution are reported in the AUXNEE file (40 instances for CUT and 40 per year for VUT).

#### Random uncertainty for NEE

In addition to the uncertainty estimates based on multiple thresholds for USTAR filtering, the random uncertainty for NEE is also estimated based on the method used by Hollinger & Richardson^37^. Variables expressing random uncertainty are identified by the suffix _RANDUNC. One of two methods are used to estimate random uncertainty, applied hierarchically:

- **NEE-RANDUNC Method 1 (direct standard deviation method):** For a sliding window of ±7 days and ±1 hour of the time-of-day of the current timestamp, the random uncertainty is calculated as the standard deviation of the measured fluxes. The similarity in the meteorological conditions evaluated as in the MDS gap-filling method^31^ and a minimum of five measured values must be present; otherwise, method 2 is used.
- **NEE-RANDUNC Method 2 (median standard deviation method):** For a sliding window of ±5 days and ±1 hour of the time-of-day of the current timestamp, random uncertainty is calculated as the median of the random uncertainty (calculated with NEE-RANDUNC Method 1) of similar fluxes, i.e., within the range of ±20% and not less than 2 µmolCO_2_ m^–2^ s^–1^. (*Note on temporal aggregations: differing sliding windows are: WW: ±2 weeks, MM: ±1 month, and YY: ±2 years*.)

The joint uncertainty for NEE is computed from the combination of the uncertainty from multiple USTAR thresholds and random uncertainty. These variables identified by the _JOINTUNC suffix and are computed for NEE filtered using the VUT method as in Eq. (5), and similarly for NEE filtered with the CUT method.

5 $$NEE{\rm{\_}}VUT{\rm{\_}}REF{\rm{\_}}JOINTUNC\,=\sqrt{NEE{\rm{\_}}VUT{\rm{\_}}REF{\rm{\_}}RANDUN{C}^{2}+{\left(\frac{NEE{\rm{\_}}VUT{\rm{\_}}84-NEE{\rm{\_}}VUT{\rm{\_}}16}{2}\right)}^{2}}$$

The 16th and 84th percentiles are used because they are equivalent to ±1 Standard Deviation in case of a normal distribution. (*Note on temporal aggregations: joint uncertainties for NEE are recomputed at all temporal resolutions*.)

#### CO_2_ flux partitioning in GPP and RECO

Partitioning CO_2_ fluxes from NEE into estimates of its two main components, Gross Primary Production (GPP) and Ecosystem Respiration (RECO), was done by parameterizations of models using measured data. All sites were partitioned with the nighttime fluxes method^31^ (_NT suffixes) and the daytime fluxes method^43^ (_DT suffixes), while a third method, sundown reference respiration^44^ (_SR suffixes), was applied to all sites meeting the method’s requirements (e.g., high quality storage measurement).

The nighttime method uses nighttime data to parameterize a respiration-temperature model that is then applied to the whole dataset to estimate RECO. GPP is then calculated as the difference between RECO and NEE. The parameterization uses short windows of time (14 days) to account for the dynamic of other important respiration drivers such as water, substrate availability, and phenology (see Reichstein *et al*.^31^ for details on the implementation and ONEFlux^22^ for the code).

The daytime method uses daytime and nighttime data to parameterize a model with one component based on a light-response curve and vapor pressure deficit for GPP, and a second component using a respiration-temperature relationship similar to the nighttime method. In this case, NEE becomes a function of both GPP and RECO, both of which are estimated by the model. Similarly to the nighttime method, the parameterization is done for short windows (8 days) to take into consideration other slower-changing factors (see Lasslop *et al*.^43^ for details on the implementation and ONEFlux^22^ for the code).

For forest sites where a CO_2_ concentration profile for storage fluxes was available, an additional RECO estimate was calculated using the method from van Gorsel *et al*.^44^, with variables identified by the _SR suffix. In this method, the parameterization of a respiration-temperature model is based solely on data acquired just after sundown, aiming at excluding the measurements potentially affected by advection and also assuming that in the first hour of the evening the advective transport is not yet established.

The sundown partitioning method requires that the NEE is not filtered for low turbulence conditions (USTAR), and for this reason it was applied only to the original time series. The nighttime and daytime methods instead require NEE filtered for low turbulence conditions. For this reason they were applied to all the 40 NEE versions resulting from the 40 USTAR thresholds, obtaining 40 versions of GPP and RECO for each of the two partitioning methods, propagating the uncertainty from NEE to GPP and RECO. This has been done for both the CUT and VUT filtering methods.

Similarly to NEE, the 40 GPP and RECO estimates (for each method and for CUT and VUT) have been used to calculate the percentiles of their distribution for each timestep (describing their uncertainty due to the NEE uncertainty). The average value (_MEAN) and the reference value use the same model efficiency approach used for NEE for each temporal aggregation. Similarly, NEE filtered with the median USTAR value (_USTAR50) has been partitioned into GPP and RECO. Information on the threshold values used for all versions of GPP and RECO (_NT and _DT, _VUT and _CUT, HH to YY resolutions) are in the auxiliary files for NEE processing (AUXNEE). Variables for reference GPP and RECO are also identified by a _REF suffix. The two methods for the partitioning (three for the cases in which the sundown method is applied) are not merged in any way, because their difference is informative with the respect to the uncertainty of the methods, as in the case of a model comparison exercise.

### Implementation approach

To increase the traceability of changes between versions of datasets and reduce uncertainty stemming from choices made at implementation time, we favored using original code implementations or thoroughly validated re-implementations of original codes. Thus, our code organization strings together loosely coupled components which implement each step, with clear-cut interfaces between steps. This modular approach eases the maintenance and change efforts for any individual step, but adds complexity to evaluating changes for the entire pipeline. Different programming languages (Python, C, MATLAB and IDL, plus PV-WAVE for FLUXNET2015) were used to implement the different steps, all connected using a controller code that makes appropriate calls in the correct order. The ONEFlux^22^ code collection replaced the PV-WAVE code with a re-implementation in Python, and also collates most of these steps into a cohesive pipeline (see also the Code Availability section). The IDL code, which applies the sundown partitioning method^44^, is not yet currently implemented in ONEFlux, because some additional testing and development are needed to make it robust and more suitable for general application. Implementation details of individual steps are discussed next, with references to the outputs each step identified by an execution sequential number and the step name–e.g., 01_qc_visual contains the results of the first processing step, the visual check step. Each of these steps correspond to a code module. Supplementary Fig. SM2 shows the steps and their inter-dependencies.

#### Steps implemented in python

The main controller code for ONEFlux is implemented in Python. Besides being the glue code that executes each step, pre- and post-checks are also executed before and after each step. These checks guarantee that the input data meet the minimum requirements to run the step, that the minimum expected outputs were generated by the execution of the step, and that any errors or exception conditions were handled correctly. Information about execution is recorded in a log for the entire pipeline, along with logs for individual steps. Besides the controller code, two of the three flux partitioning steps were re-implemented in Python (the nighttime and daytime methods, 10_nee_partition_nt and 11_nee_partition_dt), together with other specific steps such as data preparation for the uncertainty estimates (12_ure_input), and the creation and checking of final products (99_fluxnet2015). The original flux partitioning implementation in PV-WAVE was used for the LaThuile2007 and FLUXNET2015 datasets. Also, the tool for the downscaling of the ERA-I meteorological data is implemented in Python and runs on a server connected to the ERA data.

#### Steps implemented in C

Several steps are implemented in the C programming language, allowing better control over execution performance of these steps. These steps include:

automated QA/QC flagging (02_qc_auto), USTAR threshold estimation using the MP method (04_ustar_mp), the filtering and gap-filling of meteorological data, including the merging with the ERA-I downscaled data (07_meteo_proc), the filtering and gap-filling of CO_2_ fluxes (08_nee_proc), the filtering, gap-filling, and energy corrections of energy fluxes (09_energy_proc), and the computation of uncertainty products (12_ure). The source codes and the compiled executables are provided for steps implemented in C, as well as build procedures in make/Makefile format.

#### Steps implemented in MATLAB

The estimation of USTAR thresholds using the CP method (05_ustar_cp) is the only step implemented in MATLAB. It is distributed both as source code and compiled code to be used with the MATLAB Runtime Environment, such that it does not require a license purchase.

## Data Records

**Fig. 6:**
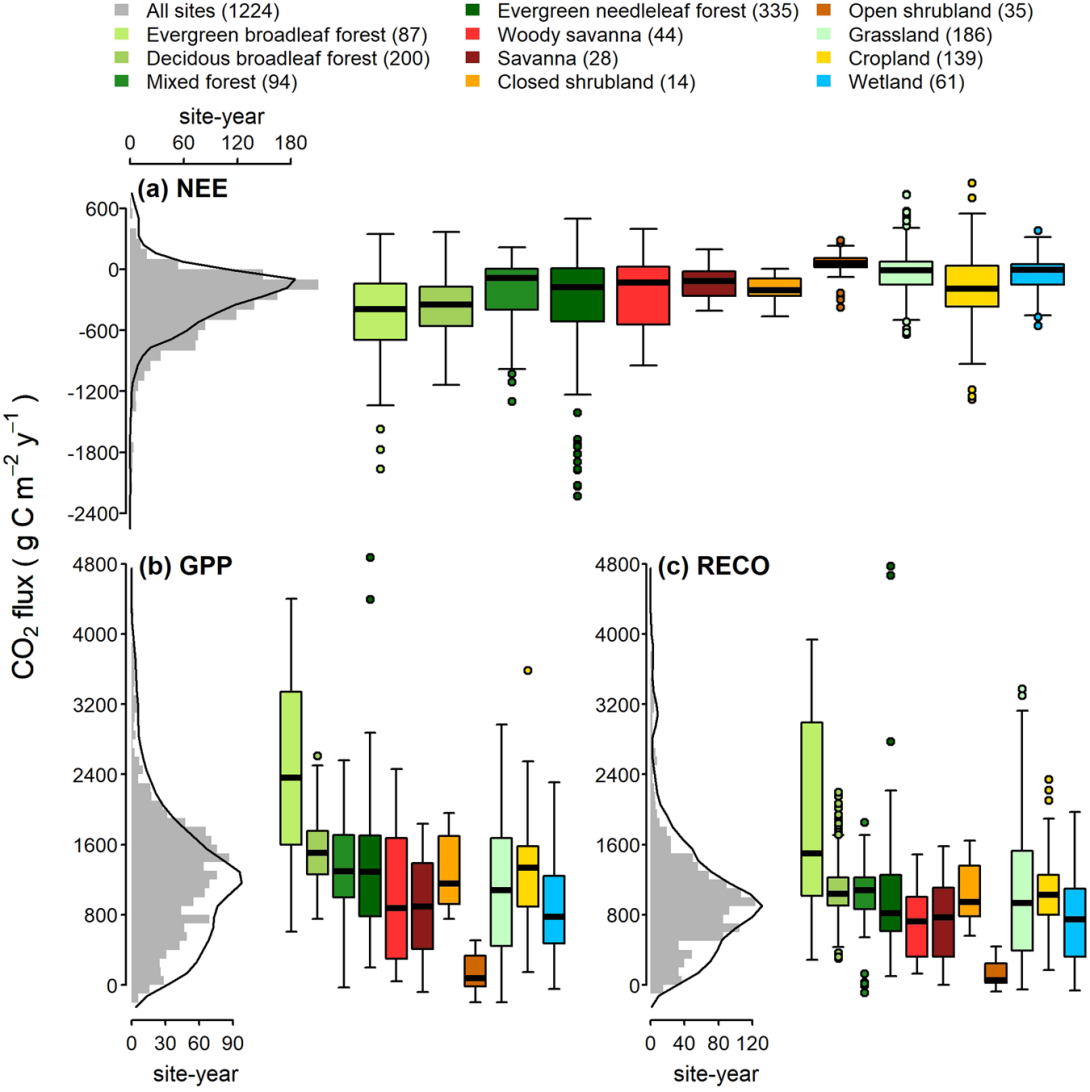
Distribution of the yearly (**a**) net ecosystem exchange (NEE), (**b**) gross primary production (GPP), and (**c**) ecosystem respiration (RECO) in FLUXNET2015. Only data with QC flag (NEE_VUT_REF_QC) higher than 0.5 are shown here. The values are reference NEE, GPP, and RECO based on the Variable USTAR Threshold (VUT) and selected reference for model efficiency (REF). GPP and RECO are based on the nighttime partitioning (NT) method. The grey histogram (bin width 100 gC m^−2^ y^−1^) shows the flux distribution in 1224 of the available site-years; negative GPP and RECO values are kept to preserve distributions, see Data processing methods section for details. Black lines show the distribution curves based on published data^253,254^. The boxplots show the flux distribution (i.e., 25th, 50th, and 75th percentiles) for vegetation types defined and color-coded according to IGBP (International Geosphere–Biosphere Programme) definitions. Circles represent data points beyond the 1.5-times interquartile range (25th to 75th percentile) plus the 75th percentile or minus 25th percentile (whisker). Numbers in parentheses indicate the number of site-years used in each IGBP group. The NO-Blv site from the snow/ice IGBP group is not shown in the boxplots.

The FLUXNET2015 portion presented in this paper contains 1496 site-years of data from 206 sites^45–250^, characterizing ecosystem-level carbon and energy fluxes in diverse ecosystems across the globe (Fig. 1, Supplementary Fig. SM1^251,252^), spanning from the early 1990s to 2014, with 69 sites having decade-long records. The dataset covers the distribution of ecosystem fluxes as reported in the recent meta-analyses^253,254^ (Fig. 6).

The dataset is distributed in files separated by sites, by temporal aggregation resolutions (e.g., hourly, weekly), and by data products (e.g., FULLSET with all the variables and SUBSET designed for less experienced users). All data files for a site are available for download as a single ZIP file archive with site-specific DOI. The file-naming conventions details these options for each file (Table 1). Site metadata are also available as a single file containing metadata for all sites, detailed later in this section and Supplementary Table SM8. Note that DOIs are assigned at the site level, one DOI per site for all of that site’s products. A DOI was not assigned to the whole FLUXNET2015 dataset, since this would make citation and assigning credit imprecise and hard to track.

**Table 1:** The template of file naming conventions, including the field, field definition, and the possible options.

File Name Conventions		
FLX_[SITE_ID]_FLUXNET2015_[DATA_PRODUCT]_[RESOLUTION]_[FIRST_YEAR]-[LAST_YEAR]_ [SITE_VERSION]-[CODE_VERSION].[EXT]		
Field	Definition	Possible options
SITE_ID	FLUXNET site ID in the format CC-SSS (CC is two-letter country code, SSS is three-character site-level identifier)	
DATA_PRODUCT	Grouping of variables from release included in file.	• SUBSET: Core set of variables with quality and uncertainty information needed for general uses of the data • FULLSET: All variables, including all quality and uncertainty information, and key variables from intermediate processing steps • AUXMETEO: Auxiliary variables related to meteorological downscaling • AUXNEE: Auxiliary variables related to NEE, RECO, and GPP processing • ERAI: Full record (1989–2014) of ERA-Interim downscaled meteorological variables for the site
RESOLUTION	Temporal resolution of data product	• HH: Half-Hourly time steps • HR: Hourly time steps • DD: Daily time steps • WW: Weekly time steps • MM: Monthly time steps • YY: Yearly time steps
FIRST_YEAR LAST_YEAR	First and last years of eddy covariance flux data	
SITE_VERSION CODE_VERSION	Version string in integer. SITE_VERSION indicates the version of the original dataset for the site used; CODE_VERSION indicates the version of the code of the data processing pipeline used to process the dataset for the site	
EXT	File extension	• csv: Comma-separated values in a text file (ASCII) • zip: Archive file with all temporal resolutions for the same site and data product
**Examples of file names and structures:** FLX_US-Ha1_FLUXNET2015_FULLSET_HH_1992-2012_1-3.zip - FLX_US-Ha1_FLUXNET2015_FULLSET_HH_1992-2012_1-3.csv - FLX_US-Ha1_FLUXNET2015_FULLSET_DD_1992-2012_1-3.csv - FLX_US-Ha1_FLUXNET2015_FULLSET_WW_1992-2012_1-3.csv - FLX_US-Ha1_FLUXNET2015_FULLSET_MM_1992-2012_1-3.csv - FLX_US-Ha1_FLUXNET2015_FULLSET_YY_1992-2012_1-3.csv - FLX_US-Ha1_FLUXNET2015_ERAI_HH_1992-2012_1-3.csv - FLX_US-Ha1_FLUXNET2015_ERAI_DD_1992-2012_1-3.csv - FLX_US-Ha1_FLUXNET2015_ERAI_WW_1992-2012_1-3.csv - FLX_US-Ha1_FLUXNET2015_ERAI_MM_1992-2012_1-3.csv - FLX_US-Ha1_FLUXNET2015_ERAI_YY_1992-2012_1-3.csv - FLX_US-Ha1_FLUXNET2015_AUXMETEO_1992-2012_1-3.csv - FLX_US-Ha1_FLUXNET2015_AUXNEE_1992-2012_1-3.csv		

Examples of file names from a zipped file of a single site are provided.

The FLUXNET2015 dataset provides data at five temporal resolutions. Site teams contribute either half-hourly (HH) or hourly (HR) datasets, depending on the integration/aggregation time decided by the site managers and function of the characteristics of the turbulence. References to half-hourly in this paper also apply to hourly data, unless explicitly stated otherwise. Half-hourly data are the basis of all the processing done for this dataset and are the finest grained temporal resolution provided. Coarser aggregations are generated uniformly from half-hourly data within the data processing pipeline. The other standard temporal aggregations are: daily (DD), weekly (WW), monthly (MM), and yearly (YY).

The complete output from the data processing pipeline includes over 200 variables–among which are measured and derived data, quality flags, uncertainty quantification variables, and results from intermediate data processing steps. The variable names follow the naming conventions of <BASENAME>_<QUALIFIER>, where BASENAME describes the physical quantities (e.g., TA, NEE, Table 2) and QUALIFIER describes the information of processing methods (e.g., VUT, CUT), uncertainties (e.g., RANDUNC), and quality flags (e.g., QC) (see Supplementary Table SM1).

**Table 2:** List of the variable basenames, descriptions, available resolutions and units.

Basename	Description	Units by Resolution				
		HH/HR	DD	WW	MM	YY
TA	Air temperature	deg C				
SW_IN_POT	Shortwave radiation, incoming, potential (top of atmosphere)	W m^−2^				
SW_IN	Shortwave radiation	W m^−2^				
LW_IN	Longwave radiation, incoming	W m^−2^				
VPD	Vapor Pressure saturation Deficit	hPa				
PA	Atmospheric pressure	kPa				
P	Precipitation	mm	mm d^−1^			mm y^−1^
WS	Wind speed	m s^−1^				
WD	Wind direction	Decimal degrees	n/a			
RH	Relative humidity	%	n/a			
USTAR	Friction velocity	m s^−1^				
NETRAD	Net radiation	W m^−2^				
PPFD_IN	Photosynthetic photon flux density, incoming	µmolPhoton m^−2^ s^−1^				
PPFD_DIF	Photosynthetic photon flux density, diffuse incoming	µmolPhoton m^−2^ s^−1^				
PPFD_OUT	Photosynthetic photon flux density, outgoing	µmolPhoton m^−2^ s^−1^				
SW_DIF	Shortwave radiation, diffuse incoming	W m^−2^				
SW_OUT	Shortwave radiation, outgoing	W m^−2^				
LW_OUT	Longwave radiation, outgoing	W m^−2^				
CO_2_	CO_2_ mole fraction	µmolCO_2_ mol^−1^				
TS	Soil temperature	deg C				
SWC	Soil water content	%				
G	Soil heat flux	W m^−2^				
LE	Latent heat flux	W m^−2^				
H	Sensible heat flux	W m^−2^				
NEE	Net Ecosystem Exchange	µmolCO_2_ m^−2^ s^−1^	gC m^−2^ d^−1^			gC m^−2^ y^−1^
RECO	Ecosystem Respiration	µmolCO_2_ m^−2^ s^−1^	gC m^−2^ d^−1^			gC m^−2^ y^−1^
GPP	Gross Primary Production	µmolCO_2_ m^−2^ s^−1^	gC m^−2^ d^−1^			gC m^−2^ y^−1^

Separate units are listed if different units are used in different temporal aggregation resolutions. n/a indicates a variable is not provided at the specified resolution.

To serve the users with an easier-to-use data product, we created two variants with different selections of variables for data distribution: the FULLSET with all the results and variables; and the SUBSET, designed to help non-expert users, with a reduced set of variables that should fit most needs.

- **FULLSET**: variables generated by the processing such as uncertainty quantification variables, all variants of the data products, all quality information flags, and many variables generated by intermediate processing steps to allow in-depth understanding of individual processing steps and their effect in the final data products. A summary of the main variable basenames is in Table 2, while a full list of variables is provided in Supplementary Table SM1. Key features of the FULLSET version are:
  - Meteorological variables filled with multiple gap-filling methods (e.g., MDS, ERA) are provided separately.
  - NEE versions filtered with two different methods of extracting the USTAR thresholds (i.e., CUT, VUT) are provided. Multiple percentiles and reference NEE are also provided.
  - GPP and RECO partitioned from NEE filtered with VUT and CUT methods, using both daytime and nighttime partitioning methods (i.e., NT, DT). Multiple percentiles and reference GPP and RECO are provided.
  - LE and H gap-filled, adjusted and non-adjusted for energy balance closure, are both provided.
  - Random, methodological, and joint uncertainties for NEE, GPP, RECO, LE, and H are provided.
- **SUBSET**: Includes a subset of the data product. The selection of the variables for this data product was done based on the expected usage for most users and to help less experienced users. Although the number of variables used is reduced, they are still accompanied by a set of quality flags and uncertainty quantification variables essential to correctly interpret the data. Key features of the SUBSET version are:
  - Only the consolidated gap-filled meteorological variables are provided.
  - Only the REF version of NEE filtered with the VUT method is provided. Selected percentiles and reference NEE are also provided.
  - GPP and RECO (only REF versions) partitioned from NEE filtered with only the VUT method, using both daytime and nighttime partitioning methods, are provided. Selected percentiles and reference GPP and RECO are also provided.
  - LE and H gap-filled, adjusted and non-adjusted for energy balance closure, are both provided.
  - Random and methodological uncertainties for NEE, GPP, RECO, LE, and H are provided.

The variable proposed in the SUBSET product is NEE_VUT_REF since it maintains the temporal variability (as opposed to the MEAN NEE), it is representative of the ensemble, and the VUT method is sensitive to possible changes of the canopy (density and height) and site setup, which can have an impact on the turbulence and consequently on the USTAR threshold. The RECO and GPP products in SUBSET are calculated from the corresponding NEE variables filtered with the VUT method, generating RECO_NT_VUT_REF and RECO_DT_VUT_REF for RECO, and GPP_NT_VUT_REF and GPP_DT_VUT_REF for GPP. It is important to use both daytime (DT) and nighttime (NT) variables, and consider their difference as uncertainty.

Auxiliary data products provide extra information on specific parameters of the data processing pipeline. The groups of products are:

- **AUXMETEO**: Auxiliary data product containing information about the downscaling of meteorological variables using the ERA-Interim reanalysis data product (TA, PA, VPD, WS, P, SW_IN, and LW_IN). Variables in these files relate to the linear regression and error/correlation estimates for each data variable used in the downscaling.
  - Parameters:
    - ERA_SLOPE: the slope of linear regression
    - ERA_INTERCEPT: intercept point of linear regression
    - ERA_RMSE: root mean square error between site data and downscaled data
    - ERA_CORRELATION: correlation coefficient of linear fit
- **AUXNEE**: Auxiliary data product with variables resulting from the processing of NEE (mainly related to USTAR filtering) and generation of RECO and GPP. Variables in this product include success/failure of execution of USTAR filtering methods, USTAR thresholds applied to different versions of variables, and percentile/threshold pairs with best model efficiency results.
  - Variables:
    - USTAR_MP_METHOD: Moving Point Test USTAR threshold method run
    - USTAR_CP_METHOD: Change Point Detection USTAR threshold method run
    - NEE_USTAR50_[UT]: 50th percentile of USTAR thresholds obtained from bootstrapping and sed to generate NEE_USTAR50_[UT] (with UT either CUT or VUT)
    - NEE_[UT]_REF: USTAR threshold used to calculate the reference NEE, using model efficiency approach (with UT either CUT or VUT)
    - [PROD]_[ALG]_[UT]_REF: USTAR threshold used to filter the NEE that was used to produce the reference product PROD (RECO or GPP), selected using model efficiency approach, using algorithm ALG (NT, DT) (with UT either CUT or VUT)
  - Parameters:
    - SUCCESS_RUN: 1 if a run of a method (USTAR_MP_METHOD or USTAR_CP_METHOD) was successful, 0 otherwise
    - USTAR_PERCENTILE: percentile of USTAR thresholds from bootstrapping at USTAR filtering step
    - USTAR_THRESHOLD: USTAR threshold value corresponding to USTAR_PERCENTILE
    - [RR]_USTAR_PERCENTILE: percentile of USTAR thresholds from bootstrapping at USTAR filtering step at resolution RR (HH, DD, WW, MM, YY)
    - [RR]_USTAR_THRESHOLD: USTAR threshold value corresponding to USTAR_PERCENTILE at resolution RR (HH, DD, WW, MM, YY)
- **ERAI**: Auxiliary data product containing full record (1989–2014) of downscaled meteorological variables using the ERA-Interim reanalysis data product, including TA, PA, VPD, WS, P, SW_IN, and LW_IN.

The FLUXNET2015 metadata are included in a single file (FLX_AA-Flx_BIF_[RESOLUTION]_[YYYYMMDD].xlsx) for all sites for each data product resolution (see Table 1 for resolution options). The metadata follow the Biological, Ancillary, Disturbance, and Metadata (BADM^255,256^) standards and are provided in the BADM Interchange Format^29^ (BIF). Table 3 illustrates the type of metadata included with selected metadata variables (See full lists and descriptions of the metadata in Supplementary Tables SM2–SM7). Height and instrument models for the flux variables, as well as soil temperature and moisture depths, are reported in the Variable Information metadata.

**Table 3:** Metadata types and selected variables. See Supplementary Tables SM2–SM7 for a full list of metadata with descriptions. Variables collected from or generated for all sites are in bold.

Metadata Type	Selected Metadata Variables
Site General Info *(25 variables)*	**SITE_ID**: Unique site identifier (CC-sss, where CC is the country code) **SITE_NAME**: Site name SITE_DESC: Site description **LOCATION_LAT:** Latitude of site **LOCATION_LONG**: Longitude of site FLUX_MEASUREMENTS_VARIABLE: Flux variables measured at the site **IGBP**: Vegetation type based on International Geosphere-Biosphere Programme classification **UTC_OFFSET**: Offset from UTC of site data **TEAM_MEMBER_NAME**: Team member name MAT, MAP: Mean annual temperature and precipitation TOWER_TYPE, TOWER_POWER: Type of tower and power type
DOI *(12 variables)*	**DOI**: Digital Object Identifier (DOI) for the flux-met data product DOI_CONTRIBUTOR_NAME: Name of contributor to the development of data (and associated info) DOI_ORGANIZATION: Organization contributing to the data
Reference publications *(4 variables)*	REFERENCE_PAPER: Reference for understanding the site REFERENCE_DOI: DOI of the reference REFERENCE_USAGE: Suggested usage of the reference
Canopy Height *(2 variables)*	**HEIGHTC**: Canopy height. In a forest ecosystem, canopy height is the distribution of overstory trees that see light at the top of the canopy. *Note: The reported value is representative of the mean of such a distribution*. **HEIGHTC_DATE**: Date of canopy height observation
Variable Information *(5 variables)* *Note: Variable Information groups are only reported for variables with data*.	**VAR_INFO_VARNAME**: Variable name **VAR_INFO_UNIT**: Variable unit **VAR_INFO_DATE**: Start date for reported variable information VAR_INFO_HEIGHT: Height/depth of observation (meters) VAR_INFO_MODEL: Model(s) used to collect observation.

## Technical Validation

Eddy covariance measurements offer a direct method to estimate trace gas or energy exchanges between surface and atmosphere at an ecosystem scale (approximately up to 1 km around the measurement point). This makes eddy covariance difficult to compare with other methods. Nonetheless, eddy covariance data have been extensively used in numerous scientific papers and studies that indirectly validate their reliability and usefulness. Hundreds of articles have been published based on eddy covariance measurements; examples of multi-site studies using FLUXNET2015 data include Jung *et al*.^15^, Tramontana *et al*.^257^, and Keenan *et al*.^258^.

Eddy covariance data were evaluated with respect to other methods such as inventory and chambers by Campioli *et al*.^259^, who showed that “EC [eddy covariance] biases are not apparent across sites, suggesting the effectiveness of standard post-processing procedures. Our results increase confidence in EC …”. The approach of Campioli *et al*.^259^ requires sites that have several additional (and rare) pieces of information; therefore, it is not generally applicable, particularly not across the sites used in this study. However, the eddy covariance site teams co-authoring this paper have compared and technically validated their measurements with respect to knowledge of their site. Unavoidably, measurement and processing uncertainties exist, and can be large for certain sites and ecosystem conditions. However, in general, flux values provided in this dataset are consistent with expectations, and eddy covariance remains one of the more reliable techniques for assessing land-air exchanges at ecosystem scales.

## Usage Notes

Detailed documentation on how to use and interpret FLUXNET2015 is available online at https://fluxnet.fluxdata.org/data/fluxnet2015-dataset/. Here, we present some of the main points to guide the usage of the data.

### Risks in the application of standard procedures

When standardized procedures are applied across different sites, the possible differences owing to data treatment are avoided or minimized; this is one of the main goals of FLUXNET2015 and ONEFlux. However, there is also the risk and possibility that the standard methods don’t work properly or as expected at specific sites and under certain conditions. This is particularly true for the CO_2_ flux partitioning, which as with all models is based on assumptions that could not always be not valid. For this reason, it could be necessary to contact the site PIs that are listed in Supplementary Table SM9.

### Using the QC flags

There are quality-control flag variables in the dataset to help users filter and interpret variables, especially for gap-filled and process knowledge-based variables. These flags are described in the variable documentation (Supplementary Table SM1). It is highly recommended that one carefully considers the QC flags when using the data.

### Percentile variants for fluxes and reference values

For most flux variables, there are reference values and percentile versions of the variables to help understand some of the uncertainty in the record. For NEE, RECO, and GPP, the percentiles are generated from the bootstrapping of the USTAR threshold estimation step, i.e., they characterize the variability from a range of values obtained as USTAR thresholds. In addition, three different reference values are provided (“_MEAN”, “_USTAR50” and “_REF”) in order to cover different user needs. In general the “_REF” version should be the most representative, particularly if related to the percentiles. It is, however, important to clearly refer to which NEE version is used in order to ensure reproducibility. For the energy balance corrected H and LE variables, the percentiles indicate the variability due to the uncertainty in the correction factor applied. Similarly to NEE, there are gap-filled and energy balance corrected versions of H and LE variables; therefore, it is also important to clearly refer to which version is used. The SUBSET version of the dataset includes a reduced number of variables, selected for non-expert users. We encourage users to carefully evaluate their requirements and options in the dataset, and if needed to contact regional networks, site teams, or even co-authors of this article for help and recommendations. For more detail, see the Methods section above.

### Temporally aggregated resolutions

All data products are provided at multiple temporal resolutions where feasible. The finest resolution is either hourly or half-hourly (indicated by the filename tags HR and HH, respectively). These data are then aggregated into daily (DD), 7-day weekly (WW), monthly (MM), and yearly (YY) resolutions, with appropriate aggregations for each variable, such as averaging for TA and summation for P.

### Timestamps

Timestamps in the data and metadata files use the format YYYYMMDDHHMM, truncated at the appropriate resolution (e.g., YYYYMMDD for a date or YYYYMM for a month). Two formats of time associated with a record are used: (1) single timestamp, where the meaning of the timestamp is unambiguous, and, (2) a pair of timestamps, indicating the start and end of the period the timestamps represent.

### Time zones

To allow more direct site comparability, all time variables are reported in local standard time (i.e., without daylight saving time). The time zone information with respect to UTC time is reported in the site metadata.

### Numeric resolution

The floating point numbers are maintained at their original resolution throughout processing steps, using double precision for the majority of cases, and are truncated at up to nine decimal places in the distributed files for numbers between 0.0 and 1.0, and at up to five decimal places for larger numbers.

### Column ordering

The order of columns is not always the same in different files (e.g., different sites). User data-processing routines should use the variable label (which is always consistent) and not the order of occurrence of that variable in the file. Timestamps are the only exception and will always be the first variable(s)/column(s) of the data file. This applies to text file data representations (i.e., CSV formatted).

### Missing data

Missing data values are indicated with −9999, without decimal points, independent of the cause of the missing value.

### Known issues

A list of known issues and limitations relevant to the dataset is maintained online: http://fluxnet.fluxdata.org/data/fluxnet2015-dataset/known-issues/.

### Releases of the FLUXNET2015 dataset

The original FLUXNET2015 release was in December 2015, followed by incremental releases in July 2016 and November 2016, and, finally, a release in February 2020 with fixes and additional metadata as described in this paper. More information on the releases can be found in the online change log: http://fluxnet.fluxdata.org/change-log/. A newer release replaces all previous ones, and only the newest release is available for direct download. Access to previous versions can be obtained upon request.

### Support to FLUXNET2015 data users

Scientists and staff responsible for the creation of the dataset offer support to data users and can be reached at fluxdata-support@fluxdata.org.

### Updates and future versions

There is strong interest and engagement in order to ensure the availability of new data (new sites and new years), keeping the open policy and the high quality data that we tried to reach with this work. We expect that the processing pipeline and QA/QC procedures will continue evolving, in support of new products. However, the amount of both technical and coordination work, along with difficulty securing long-term international funding, hamper creation of new versions of the dataset. There are ongoing discussions among regional networks and FLUXNET on this coordination, but currently there is no plan for a follow-up version of FLUXNET2015.

## Supplementary information

Supplementary Materials

## Data Availability

The ONEFlux collection of codes used to create data intercomparable with FLUXNET2015 has been packaged to be executed as a complete pipeline and is available in both source-code and executable forms under a 3-clause BSD license on GitHub: https://github.com/AmeriFlux/ONEFlux. The complete environment to run this pipeline requires a GCC compatible C compiler (or capability to run pre-compiled Windows, Linux, and/or Mac executables), a MATLAB Runtime Environment, and a Python interpreter with a few numeric and scientific packages installed. All of these can be obtained at no cost.
