# Author Correction: The FLUXNET2015 dataset and the ONEFlux processing pipeline for eddy covariance data

**DOI:** 10.1038/s41597-021-00851-9

**Published:** 2021-02-25

**Authors:** Gilberto Pastorello, Carlo Trotta, Eleonora Canfora, Housen Chu, Danielle Christianson, You-Wei Cheah, Cristina Poindexter, Jiquan Chen, Abdelrahman Elbashandy, Marty Humphrey, Peter Isaac, Diego Polidori, Markus Reichstein, Alessio Ribeca, Catharine van Ingen, Nicolas Vuichard, Leiming Zhang, Brian Amiro, Christof Ammann, M. Altaf Arain, Jonas Ardö, Timothy Arkebauer, Stefan K. Arndt, Nicola Arriga, Marc Aubinet, Mika Aurela, Dennis Baldocchi, Alan Barr, Eric Beamesderfer, Luca Belelli Marchesini, Onil Bergeron, Jason Beringer, Christian Bernhofer, Daniel Berveiller, Dave Billesbach, Thomas Andrew Black, Peter D. Blanken, Gil Bohrer, Julia Boike, Paul V. Bolstad, Damien Bonal, Jean-Marc Bonnefond, David R. Bowling, Rosvel Bracho, Jason Brodeur, Christian Brümmer, Nina Buchmann, Benoit Burban, Sean P. Burns, Pauline Buysse, Peter Cale, Mauro Cavagna, Pierre Cellier, Shiping Chen, Isaac Chini, Torben R. Christensen, James Cleverly, Alessio Collalti, Claudia Consalvo, Bruce D. Cook, David Cook, Carole Coursolle, Edoardo Cremonese, Peter S. Curtis, Ettore D’Andrea, Humberto da Rocha, Xiaoqin Dai, Kenneth J. Davis, Bruno De Cinti, Agnes de Grandcourt, Anne De Ligne, Raimundo C. De Oliveira, Nicolas Delpierre, Ankur R. Desai, Carlos Marcelo Di Bella, Paul di Tommasi, Han Dolman, Francisco Domingo, Gang Dong, Sabina Dore, Pierpaolo Duce, Eric Dufrêne, Allison Dunn, Jiří Dušek, Derek Eamus, Uwe Eichelmann, Hatim Abdalla M. ElKhidir, Werner Eugster, Cacilia M. Ewenz, Brent Ewers, Daniela Famulari, Silvano Fares, Iris Feigenwinter, Andrew Feitz, Rasmus Fensholt, Gianluca Filippa, Marc Fischer, John Frank, Marta Galvagno, Mana Gharun, Damiano Gianelle, Bert Gielen, Beniamino Gioli, Anatoly Gitelson, Ignacio Goded, Mathias Goeckede, Allen H. Goldstein, Christopher M. Gough, Michael L. Goulden, Alexander Graf, Anne Griebel, Carsten Gruening, Thomas Grünwald, Albin Hammerle, Shijie Han, Xingguo Han, Birger Ulf Hansen, Chad Hanson, Juha Hatakka, Yongtao He, Markus Hehn, Bernard Heinesch, Nina Hinko-Najera, Lukas Hörtnagl, Lindsay Hutley, Andreas Ibrom, Hiroki Ikawa, Marcin Jackowicz-Korczynski, Dalibor Janouš, Wilma Jans, Rachhpal Jassal, Shicheng Jiang, Tomomichi Kato, Myroslava Khomik, Janina Klatt, Alexander Knohl, Sara Knox, Hideki Kobayashi, Georgia Koerber, Olaf Kolle, Yoshiko Kosugi, Ayumi Kotani, Andrew Kowalski, Bart Kruijt, Julia Kurbatova, Werner L. Kutsch, Hyojung Kwon, Samuli Launiainen, Tuomas Laurila, Bev Law, Ray Leuning, Yingnian Li, Michael Liddell, Jean-Marc Limousin, Marryanna Lion, Adam J. Liska, Annalea Lohila, Ana López-Ballesteros, Efrén López-Blanco, Benjamin Loubet, Denis Loustau, Antje Lucas-Moffat, Johannes Lüers, Siyan Ma, Craig Macfarlane, Vincenzo Magliulo, Regine Maier, Ivan Mammarella, Giovanni Manca, Barbara Marcolla, Hank A. Margolis, Serena Marras, William Massman, Mikhail Mastepanov, Roser Matamala, Jaclyn Hatala Matthes, Francesco Mazzenga, Harry McCaughey, Ian McHugh, Andrew M. S. McMillan, Lutz Merbold, Wayne Meyer, Tilden Meyers, Scott D. Miller, Stefano Minerbi, Uta Moderow, Russell K. Monson, Leonardo Montagnani, Caitlin E. Moore, Eddy Moors, Virginie Moreaux, Christine Moureaux, J. William Munger, Taro Nakai, Johan Neirynck, Zoran Nesic, Giacomo Nicolini, Asko Noormets, Matthew Northwood, Marcelo Nosetto, Yann Nouvellon, Kimberly Novick, Walter Oechel, Jørgen Eivind Olesen, Jean-Marc Ourcival, Shirley A. Papuga, Frans-Jan Parmentier, Eugenie Paul-Limoges, Marian Pavelka, Matthias Peichl, Elise Pendall, Richard P. Phillips, Kim Pilegaard, Norbert Pirk, Gabriela Posse, Thomas Powell, Heiko Prasse, Suzanne M. Prober, Serge Rambal, Üllar Rannik, Naama Raz-Yaseef, Corinna Rebmann, David Reed, Victor Resco de Dios, Natalia Restrepo-Coupe, Borja R. Reverter, Marilyn Roland, Simone Sabbatini, Torsten Sachs, Scott R. Saleska, Enrique P. Sánchez-Cañete, Zulia M. Sanchez-Mejia, Hans Peter Schmid, Marius Schmidt, Karl Schneider, Frederik Schrader, Ivan Schroder, Russell L. Scott, Pavel Sedlák, Penélope Serrano-Ortíz, Changliang Shao, Peili Shi, Ivan Shironya, Lukas Siebicke, Ladislav Šigut, Richard Silberstein, Costantino Sirca, Donatella Spano, Rainer Steinbrecher, Robert M. Stevens, Cove Sturtevant, Andy Suyker, Torbern Tagesson, Satoru Takanashi, Yanhong Tang, Nigel Tapper, Jonathan Thom, Michele Tomassucci, Juha-Pekka Tuovinen, Shawn Urbanski, Riccardo Valentini, Michiel van der Molen, Eva van Gorsel, Ko van Huissteden, Andrej Varlagin, Joseph Verfaillie, Timo Vesala, Caroline Vincke, Domenico Vitale, Natalia Vygodskaya, Jeffrey P. Walker, Elizabeth Walter-Shea, Huimin Wang, Robin Weber, Sebastian Westermann, Christian Wille, Steven Wofsy, Georg Wohlfahrt, Sebastian Wolf, William Woodgate, Yuelin Li, Roberto Zampedri, Junhui Zhang, Guoyi Zhou, Donatella Zona, Deb Agarwal, Sebastien Biraud, Margaret Torn, Dario Papale

**Affiliations:** 1 Computational Research Division, Lawrence Berkeley National Laboratory, Berkeley, CA 94720 USA; 2 DIBAF, University of Tuscia, Viterbo, 01100 Italy; 3 Euro-Mediterranean Centre on Climate Change Foundation (CMCC), Lecce, 73100 Italy; 4 Climate & Ecosystem Sciences Division, Lawrence Berkeley National Laboratory, Berkeley, CA 94720 USA; 5 Department of Civil Engineering, California State University, Sacramento, CA 95819 USA; 6 Department of Geography, Environment, and Spatial Sciences, Michigan State University, East Lansing, MI 48823 USA; 7 Department of Computer Science, University of Virginia, Charlottesville, VA 22904 USA; 8 TERN Ecosystem Processes, Menzies Creek, VIC3159 Australia; 9 Max Planck Institute for Biogeochemistry, Jena, 07701 Germany; 10 Laboratoire des Sciences du Climat et de l’Environnement (LSCE), CEA CNRS, UVSQ UPSACLAY, Gif sur Yvette, 91190 France; 11 Key Laboratory of Ecosystem Network Observation and Modeling, Institute of Geographic Sciences and Natural Resources Research, Chinese Academy of Sciences, Beijing, 100101 China; 12 Department of Soil Science, University of Manitoba, Winnipeg, MB R3T2N2 Canada; 13 Department of Agroecology and Environment, Agroscope Research Institute, Zürich, 8046 Switzerland; 14 School of Geography and Earth Sciences, McMaster University, L8S4K1 Hamilton, ON Canada; 15 Department of Physical Geography and Ecosystem Science, Lund University, Lund, 22362 Sweden; 16 Department of Agronomy and Horticulture, University of Nebraska-Lincoln, Lincoln, NE 68583 USA; 17 School of Ecosystem and Forest Sciences, The University of Melbourne, Richmond, VIC3121 Australia; 18 Department of Biology, Research Group PLECO, University of Antwerp, Antwerp, 2610 Belgium; 19 Joint Research Centre, European Commission, Ispra, 21027 Italy; 20 TERRA Teaching and Research Center, University of Liege, Gembloux, B-5030 Belgium; 21 Finnish Meteorological Institute, Helsinki, 00560 Finland; 22 ESPM, University of California Berkeley, Berkeley, CA 94720 USA; 23 Global Institute for Water Security, University of Saskatchewan, Saskatoon, SK S7N3H5 Canada; 24 Climate Research Division, Environment and Climate Change Canada, Saskatoon, SK S7N3H5 Canada; 25 Department of Sustainable Agro-ecosystems and Bioresources, Research and Innovation Centre, Fondazione Edmund Mach, San Michele All’adige, 38010 Italy; 26 Department of Landscape Design and Sustainable Ecosystems, Agrarian-Technological Institute, RUDN University, Moscow, 117198 Russia; 27 Direction du marché du carbone, Ministère du Développement durable de l’Environnement et de la Lutte contre les changements climatiques, Québec, QC G1R5V7 Canada; 28 School of Agriculture and Environment, University of Western Australia, Crawley, 6009 Australia; 29 Institute of Hydrology and Meteorology, Technische Universität Dresden, Tharandt, 01737 Germany; 30 Université Paris-Saclay, CNRS, AgroParisTech, Ecologie Systématique et Evolution, Orsay, 91405 France; 31 Biological Systems Engineering, University of Nebraska-Lincoln, Lincoln, NE 68583 USA; 32 Faculty of Land and Food Systems, University of British Columbia, Vancouver, BC V6T1Z4 Canada; 33 Department of Geography, University of Colorado, Boulder, CO 80309 USA; 34 Department of Civil, Environmental & Geodetic Engineering, Ohio State University, Columbus, OH 43210 USA; 35 Alfred Wegener Institute Helmholtz Centre for Polar and Marine Research, Potsdam, 14482 Germany; 36 Geography Department, Humboldt-Universität zu Berlin, Berlin, Germany; 37 Forest Resources, University of Minnesota, St Paul, MN 55108 USA; 38 Université de Lorraine, AgroParisTech, INRAE, UMR Silva, Nancy, 54000 France; 39 ISPA, Bordeaux Sciences Agro, INRAE, Villenave d’Ornon, 33140 France; 40 School of Biological Sciences, University of Utah, Salt Lake City, UT 84112 USA; 41 School of Forest Resources and Conservation, University of Florida, Gainesville, FL 32611 USA; 42 McMaster University Library, McMaster University, Hamilton, ON L8S4L6 Canada; 43 Thünen Institute of Climate-Smart Agriculture, Federal Research Institute of Rural Areas, Forestry and Fisheries, Braunschweig, 38116 Germany; 44 Department of Environmental Systems Science, ETH Zurich, Zurich, 8092 Switzerland; 45 INRAE UMR ECOFOG, Kourou, 97387 French Guiana; 46 Mesoscale and Microscale Meteorology Laboratory, National Center for Atmospheric Research, Boulder, CO 80301 USA; 47 Université Paris-Saclay, INRAE, AgroParisTech, UMR ECOSYS, Thiverval-Grignon, 78850 France; 48 Australian Landscape Trust, Renmark, SA5341 Australia; 49 State Key Laboratory of Vegetation and Environmental Change, Institute of Botany, Chinese Academy of Sciences, Beijing, 100093 China; 50 Department of Bioscience, Arctic Research Center, Aarhus University, Roskilde, 4000 Denmark; 51 School of Life Sciences, University of Technology Sydney, Sydney, 2007 Australia; 52 Terrestrial Ecosystem Research Network TERN, University of Technology, Sydney, 2007 Australia; 53 Institute for Agricultural and Forestry Systems in the Mediterranean, National Research Council of Italy, Ercolano, 80056 Italy; 54 Research Institute on Terrestrial Ecosystems, National Research Council of Italy, Porano, 05010 Italy; 55 Biospheric Sciences Laboratory, NASA Goddard Space Flight Center, Greenbelt, MD 20771 USA; 56 Environmental Science Division, Argonne National Laboratory, Lemont, IL 60439 USA; 57 Canadian Forest Service, Natural Resources Canada, Québec, QC G1V4C7 Canada; 58 Centre d’étude de la forêt, Faculté de foresterie, de géographie et de géomatique, Université Laval, Québec, QC G1V0A6 Canada; 59 Climate Change Unit, Environmental Protection Agency of Aosta Valley, Saint Christophe, 11020 Italy; 60 Department of Evolution, Ecology, and Organismal Biology, Ohio State University, Columbus, OH 43210 USA; 61 Instituto de Astronomia, Geofísica e Ciências Atmosféricas, Universidade de São Paulo, São Paulo, SP 01000-000 Brazil; 62 Department of Meteorology and Atmospheric Science, The Pennsylvania State University, University Park, PA 16802 USA; 63 Institute of Research on Terrestrial Ecosystems, National Research Council of Italy, Montelibretti, 00010 Italy; 64 UMR Eco&Sols, CIRAD, Montpellier, 34060 France; 65 Pedology, Embrapa Amazonia Oriental, Belém, PA 68020640 Brazil; 66 Atmospheric and Oceanic Sciences, University of Wisconsin-Madison, Madison, WI 53706 USA; 67 Departamento de Métodos Cuantitativos y Sistemas de Información, Facultad de Agronomía, UBA, Buenos Aires, 1417 Argentina; 68 Department of Earth Sciences, Vrije Universiteit Amsterdam, Amsterdam, 1081 HV The Netherlands; 69 Desertification and Geoecology Department, Experimental Station of Arid Zones, CSIC, Almería, 04120 Spain; 70 School of Life Science, Shanxi University, Taiyuan, 030006 China; 71 HydroFocus, Davis, CA 95618 USA; 72 Institute of BioEconomy, National Research Council of Italy, Sassari, 07100 Italy; 73 Department of Earth, Environment, and Physics, Worcester State University, Worcester, MA 01602 USA; 74 Department of Matter and Energy Fluxes, Global Change Research Institute of the Czech Academy of Sciences, Brno, 60300 Czech Republic; 75 ElObeid Research Station, Agricultural Research Corporation, ElObeid, 51111 Sudan; 76 Airborne Research Australia, TERN Ecosystem Processes Central Node, Parafield, 5106 Australia; 77 Department of Botany, Program in Ecology, University of Wyoming, 1000 E. Univ. Ave, Laramie, WY 82071 USA; 78 Institute of BioEconomy, National Research Council of Italy, Rome, 00100 Italy; 79 Research Centre for Forestry and Wood, Council for Agricultural Research and Economics, Rome, 00166 Italy; 80 Geoscience Australia, Canberra, 2601 Australia; 81 Department of Geosciences and Natural Resource Management, University of Copenhagen, Copenhagen, 1350 Denmark; 82 Energy Analysis & Environmental Impacts Division, Lawrence Berkeley National Laboratory, Berkeley, CA 94720 USA; 83 USDA Forest Service, Rocky Mountain Research Station, Fort Collins, CO 80526 USA; 84 Institute of BioEconomy, National Research Council of Italy, Firenze, 50145 Italy; 85 School of Natural Resources, University of Nebraska-Lincoln, Lincoln, NE 68583 USA; 86 Max Planck Institute for Biogeochemistry, Jena, 03641 Germany; 87 Department of Biology, Virginia Commonwealth University, Richmond, VA 23284 USA; 88 Department of Earth System Science, University of California, Irvine, CA 92697 USA; 89 Agrosphere (IBG3), Forschungszentrum Jülich, Jülich, 52428 Germany; 90 Department of Ecology, University of Innsbruck, Innsbruck, 6020 Austria; 91 International Joint Research Laboratory for Global Change Ecology, School of Life Sciences, Henan University, Kaifeng, 450000 China; 92 Institute of Applied Ecology, Chinese Academy of Sciences, Shenyang, 110016 China; 93 Department of Forest Ecosystems and Society, Oregon State University, Corvallis, OR 97333 USA; 94 College of Resources and Environment, University of Chinese Academy of Sciences, Beijing, 100190 China; 95 School of Ecosystem and Forest Sciences, The University of Melbourne, Creswick, VIC3363 Australia; 96 Research Institute for the Environment and Livelihoods, Charles Darwin University, Darwin, 0909 Australia; 97 Department of Environmental Engineering, Technical University of Denmark (DTU), Kongens Lyngby, 2800 Denmark; 98 Institute for Agro-Environmental Sciences, National Agriculture and Food Research Organization, Tsukuba, 305-8604 Japan; 99 Wageningen Environmental Research, Wageningen University and Research, Wageningen, 6708PB The Netherlands; 100 Key Laboratory of Vegetation Ecology, Ministry of Education, Northeast Normal University, Changchun, 130024 China; 101 Research Faculty of Agriculture, Hokkaido University, Sapporo, 060-8589 Japan; 102 GI-Core, Hokkaido University, Sapporo, 060-0808 Japan; 103 Geography and Environmental Management, Waterloo, ON N2L3G1 Canada; 104 Institute of Meteorology and Climate Research, Karlsruhe Institute of Technology, Garmisch-Partenkirchen, 82467 Germany; 105 Bioclimatology, University of Goettingen, Goettingen, 37077 Germany; 106 Centre of Biodiversity and Sustainable Land Use (CBL), University of Goettingen, Goettingen, 37077 Germany; 107 Department of Geography, The University of British Columbia, Vancouver, BC V6T1Z2 Canada; 108 Research Institute for Global Change, Institute of Arctic Climate and Environment Research, Japan Agency for Marine-Earth Science and Technology, Yokoama, 236-0001 Japan; 109 Biological Sciences, University of Adelaide, Adelaide, SA5064 Australia; 110 Graduate School of Agriculture, Kyoto University, Kyoto, 606-8502 Japan; 111 Graduate School of Bioagricultural Sciences, Nagoya University, Nagoya, 4648601 Japan; 112 Department of Applied Physics, University of Granada, Granada, 18071 Spain; 113 Water systems and Global Change group, Wageningen University, Wageningen, 6500 The Netherlands; 114 A.N. Severtsov Institute of Ecology and Evolution, Russian Academy of Sciences, Moscow, 119071 Russia; 115 Head Office, Integrated Carbon Observation System (ICOS ERIC), Helsinki, 00560 Finland; 116 Natural Resources Institute Finland, Helsinki, 00790 Finland; 117 Key Laboratory of Adaptation and Evolution of Plateau Biota, Northwest Institute of Plateau Biology, Chinese Academy of Sciences, Xining, 810008 China; 118 Centre for Tropical Environmental Sustainability Studies, James Cook University, Cairns, 4878 Australia; 119 CEFE, CNRS, Univ Montpellier, Montpellier, 34293 France; 120 Forestry and Environment Division, Forest Research Institute Malaysia (FRIM), Kepong, 52109 Malaysia; 121 Institute for Atmosphere and Earth System Research/Physics, University of Helsinki, Helsinki, 00560 Finland; 122 Department of Botany, School of Natural Sciences, Trinity College Dublin, Dublin, D02PN40 Ireland; 123 German Meteorological Service (DWD), Centre for Agrometeorological Research, Braunschweig, 38116 Germany; 124 Micrometeorology, University of Bayreuth, Bayreuth, 95440 Germany; 125 Bayreuth Center of Ecology and Environmental Research, 95448 Bayreuth, Germany; 126 CSIRO Land and Water, Floreat, 6014 Australia; 127 Department of Agriculture, University of Sassari, Sassari, 07100 Italy; 128 Oulanka research station, University of Oulu, Kuusamo, 93900 Finland; 129 Dept. Biological Sciences, Wellesley College, Wellesley, MA 02481 USA; 130 Research Institute on Terrestrial Ecosystems, National Research Council of Italy, Monterotondo Scalo, 00015 Italy; 131 Department of Geography and Planning, Queen’s University, Kingston, ON K7L3N6 Canada; 132 Environmental Analytics NZ, Ltd. Raumati South, Paraparaumu, 5032 New Zealand; 133 Mazingira Centre, International Livestock Research Institute (ILRI), Nairobi, 00100 Kenya; 134 NOAA/OAR/Air Resources Laboratory, 325 Broadway, Boulder, CO 80303 USA; 135 Atmospheric Sciences Research Center, State University of New York at Albany, Albany, NY 12203 USA; 136 Forest Department of South Tyrol, Bolzano, 39100 Italy; 137 Department of Ecology and Evolutionary Biology, University of Arizona, Tucson, AZ 85721 USA; 138 Faculty of Science and Technology, Free University of Bolzano, Bolzano, 39100 Italy; 139 Department of Plant Biology, University of Illinois at Urbana-Champaign, Urbana, IL 61801 USA; 140 IHE Delft, Delft, 2611 The Netherlands; 141 Faculty of Science, VU Amsterdam, Amsterdam, 1081 The Netherlands; 142 University Grenoble Alpes, IRD, CNRS, IGE, Grenoble, 38000 France; 143 School of Engineering and Applied Sciences, Harvard University, Cambridge, MA 02138 USA; 144 Department of Earth and Planetary Sciences, Harvard University, Cambridge, MA 02138 USA; 145 School of Forestry and Resource Conservation, National Taiwan University, Taipei, 0617 Taiwan; 146 International Arctic Research Center, University of Alaska Fairbanks, Fairbanks, AK 99775 USA; 147 Environment and Climate, Research Institute for Nature and Forest, Geraardsbergen, 9500 Belgium; 148 Department of Ecosystem Science and Management, Texas A&M University, College Station, TX 77843 USA; 149 Research Institute for the Environment and Livelihoods, Charles Darwin University, Casuarina, 0810 Australia; 150 Grupo de Estudios Ambientales, Instituto de Matemática Aplicada San Luis (UNSL & CONICET), San Luis, D5700HHW Argentina; 151 Facultad de Ciencias Agropecuarias (UNER), Oro Verde, 3100 Argentina; 152 Eco&Sols, Univ Montpellier-CIRAD-INRA-IRD-Montpellier SupAgro, Montpellier, 34060 France; 153 O’Neill School of Public and Environmental Affairs, Indiana University Bloomington, Bloomington, IN 47405 USA; 154 Global Change Research Group, Dept. Biology, San Diego State University, San Diego, CA 92182 USA; 155 Department of Geography, College of Life and Environmental Sciences, University of Exeter, Exeter, EX44RJ United Kingdom; 156 Department of Agroecology, Aarhus University, Tjele, 8830 Denmark; 157 iCLIMATE, Aarhus University, Tjele, 8830 Denmark; 158 Department of Geology, Wayne State University, Detroit, MI 48202 USA; 159 Department of Geosciences, University of Oslo, Oslo, 0315 Norway; 160 Department of Geography, University of Zurich, Zurich, 8057 Switzerland; 161 Department of Forest Ecology and Management, Swedish University of Agricultural Sciences, Umeå, 90183 Sweden; 162 Hawkesbury Institute for the Environment, Western Sydney University, Penrith, 2751 Australia; 163 Department of Biology, Indiana University Bloomington, Bloomington, IN 47401 USA; 164 CSIRO Land and Water, Wembley, 6913 Australia; 165 Instituto de Clima y Agua, Instituto Nacional de Tecnologia Agropecuaria (INTA), Buenos Aires, 1686 Argentina; 166 Department Computational Hydrosystems, Helmholtz Centre for Environmental Research UFZ, Leipzig, 04318 Germany; 167 Center for Global Change & Earth Observations, Michigan State University, East Lansing, MI 48823 USA; 168 School of Life Science and Engineering, Southwest University of Science and Technology, Mianyang, 621010 China; 169 Departamento de Química e Física, Universidade Federal da Paraiba, Areia, PB 58397-000 Brazil; 170 Remote Sensing and Geoinformatics, GFZ German Research Centre for Geosciences, Potsdam, 14473 Germany; 171 Andalusian Institute for Earth System Research (CEAMA-IISTA), Granada, 18006 Spain; 172 Ciencias del Agua y Medioambiente, Instituto Tecnológico de Sonora, Ciudad Obregón, 85000 Mexico; 173 Geographical Institute, University of Cologne, Cologne, 50923 Germany; 174 Department of Industry, Innovation and Science, Geoscience Australia, Canberra, 2609 Australia; 175 Southwest Watershed Research Center, USDA-ARS, Tucson, AZ 85719 USA; 176 Institute of Atmospheric Physics of the Czech Academy of Sciences, Prague, 14100 Czech Republic; 177 Department of Ecology, University of Granada, Granada, 18071 Spain; 178 National Hulunber Grassland Ecosystem Observation and Research Station & Institute of Agricultural Resources and Regional Planning, Chinese Academy of Agricultural Sciences, Beijing, 100081 China; 179 School of Science, Edith Cowan University, Joondalup, 6027 Australia; 180 Sentek Pty Ltd, Stepney, SA5069 Australia; 181 National Ecological Observatory Network Program, Boulder, CO 80301 USA; 182 Kansai Research Center, Forestry and Forest Products Research Institute, Kyoto, 612-0855 Japan; 183 College of Urban and Environmental Sciences, Peking University, Beijing, 100871 China; 184 School of Earth, Atmosphere and Environment, Monash University, Clayton, 3800 Australia; 185 Space Science and Engineering Center, University of Wisconsin-Madison, Madison, WI 53706 USA; 186 Terrasystem srl, Viterbo, 01100 Italy; 187 USDA Forest Service, Rocky Mountain Research Station, Missoula, MT 59808 USA; 188 Meteorology and Air Quality group, Wageningen University, 6500 Wageningen, The Netherlands; 189 Fenner School of Environment and Society, Australian National University Canberra, Canberra, ACT 2600 Australia; 190 Earth and Life Institute, Université Catholique de Louvain, Louvain-la-Neuve, 1348 Belgium; 191 Department of Civil Engineering, Monash University, Clayton, 3800 Australia; 192 CSIRO Land and Water, Canberra, 2601 Australia; 193 School of Earth and Environmental Sciences, The University of Queensland, St Lucia, 4072 Australia; 194 South China Botanical Garden, Chinese Academy of Sciences, Guangzhou, 510650 China; 195 College of Applied Meteorology, Nanjing University of Information Science & Technology, Nanjing, 210044 China; 196 Department of Animal and Plant Sciences, University of Sheffield, Sheffield, S102TN United Kingdom

**Keywords:** Environmental sciences, Climate sciences, Carbon cycle

Correction to: *Scientific Data* 10.1038/s41597-020-0534-3, published online 09 July 2020

The following authors were omitted from the original version of this Data Descriptor: Markus Reichstein and Nicolas Vuichard. Both contributed to the code development and N. Vuichard contributed to the processing of the ERA-Interim data downscaling. Furthermore, the contribution of the co-author Frank Tiedemann was re-evaluated relative to the colleague Corinna Rebmann, both working at the same sites, and based on this re-evaluation a substitution in the co-author list is implemented (with Rebmann replacing Tiedemann). Finally, two affiliations were listed incorrectly and are corrected here (entries 190 and 193). The author list and affiliations have been amended to address these omissions in both the HTML and PDF versions.

