# Supplementary material for: The FLUXNET2015 dataset and the ONEFlux processing pipeline for eddy covariance data
Source: Sci Data. 2020 Jul 9;7:225. doi: 10.1038/s41597-020-0534-3 (PMC7347557; doi:10.1038/s41597-020-0534-3)
Supplement: Supplementary file 1 — Supplementary Materials [file 41597_2020_534_MOESM1_ESM.pdf]

Table of contents for supplementary materials

Appendix SM1. FLUXNET2015 Variables Quick Start Guide	2-SM
Supplementary Materials: Figures	4-SM
Supplementary Figure SM1. Distribution of sites by mean annual air temperature and precipitation	5-SM
Supplementary Figure SM2.The processing steps order and dependencies	6-SM
Supplementary Figure SM3. Workflow for method selection for the estimation of EBC_CF	7-SM
Supplementary Figure SM4. Continental scale maps with site positions	8-SM
Supplementary Materials: Tables	9-SM
Table SM1. Complete variables list for FLUXNET2015 dataset	*10-SM
Table SM2. Site General Information metadata	11-SM
Table SM3. DOI metadata	14-SM
Table SM4.Publications metadata	15-SM
Table SM5. Canopy Height metadata	16-SM
Table SM6. Variable Information metadata	17-SM
Table SM7. Predefined unit options	*18-SM
Table SM8. BADM Interchange Format (BIF) Description and Examples	19-SM
Table SM9. Co-author list and site affiliation	21-SM

** Long tables, placed at the end of the document.*

Appendix SM1. FLUXNET2015 Variables Quick Start Guide

This document is designed to guide non-expert users to quickly get started selecting variables from the FLUXNET2015 dataset. Key points are explained here, but we strongly recommend looking at documentation of the dataset to understand the different versions of variables, as the choice of variable can impact analysis results. See the main text of this paper and online documentation for more information, and for in depth questions, contact regional network teams or site teams.

FLUXNET2015 data products. Non-expert users can get started with the **FLUXNET2015 SUBSET** data product. Compared to the FULLSET product, SUBSET includes all the same sites and the complete temporal record, but only includes a selection of the variables, which should fit the required data for most users. All variables mentioned in this guide are available in the SUBSET data product.

Quality flags and gap-filled data. The ***_QC** variables are quality control flags for the records of associated variables, identifying originally measured or gap-filled values, along with a quality indicator in the latter case. For instance, TA_F_QC is the quality flag for the gap-filled air temperature variable TA_F. At the half-hourly or hourly resolution (HH), the _QC variable indicates if the corresponding record is a measured value (`*_QC=0`), or the quality level of the gap-filling that was used for that record (`*_QC=1` better, `*_QC=3` worse quality). At coarser temporal resolutions, i.e., daily (DD) through yearly (YY), the quality flag indicates the **percentage of measured (`*_QC=0`) or good quality gap-filled (`*_QC=1`) records** aggregated from finer temporal resolutions.

CO_2 Flux Variables. CO_2 fluxes are key in the dataset, being the variable with most related variables and versions produced. The main versions of CO_2 variables discussed in this guide cover Net Ecosystem Exchange (NEE), Ecosystem Respiration (RECO), and Gross Primary Production (GPP).

> ***NEE:*** the variable proposed in the SUBSET product is **NEE_VUT_REF** since it maintains the temporal variability (as opposed to the MEAN NEE), it is representative of the ensemble, and the VUT method is sensitive to possible changes of the canopy (density and height) and site setup. The FULLSET includes other versions such the CUT where the same USTAR threshold is used for all the years, removing possible variability in the NEE due to the threshold value or the MEAN that is the average of the ensambles (which smooths over the variability). The other NEE variables in SUBSET represent uncertainty estimates: random uncertainty from measurements (NEE_VUT_REF_RANDUNC) and the uncertainty due to the USTAR threshold-based filtering (NEE_VUT_XX; see USTAR uncertainty details below).

RECO and GPP: in the SUBSET product there are two main versions of GPP and RECO. They originate from two CO_2 flux partitioning methods adopted for FLUXNET2015: nighttime (NT) and daytime (DT). The two methods are independent, making their consistency an indicator of the robustness of the estimates. Without a context in which they are being used, it is impossible to give a-priori preference to one or the other. Our suggestion is to use both daytime (DT) and nighttime (NT) variables and consider their difference as uncertainty. Alternatively, users can filter the sites to use in the analysis based on the consistency between the two products. Being highly dependent on site and time-aggregation, the difference between the two partitioning methods can reach, at an annual time resolution, over 500 gC m-2 yr-1. The RECO and GPP products in SUBSET are calculated from the corresponding NEE variables filtered with the VUT method, generating **RECO_NT_VUT_REF** and **RECO_DT_VUT_REF** for RECO, and **GPP_NT_VUT_REF** and **GPP_DT_VUT_REF** for GPP. As for the NEE variable above, GPP and RECO also include variables describing the uncertainty due to USTAR threshold-based filtering (see USTAR uncertainty details next).

USTAR Uncertainty: the uncertainty stemming from the USTAR threshold estimation is the main source of uncertainty for this dataset. An ensemble of USTAR thresholds are applied to filter NEE and the resulting versions of NEE are represented through percentiles (NEE_VUT_XX). The effect of the USTAR threshold uncertainty is also site and time-aggregation dependent, with an interquartile range at annual scale that can be up to 200 gC m-2 year-1. The ensemble of NEE versions originated using the different thresholds are all put through the partitioning with the two methods, resulting in versions of RECO and GPP also represented by percentiles (RECO_NT_VUT_XX/RECO_DT_VUT_XX and GPP_NT_VUT_XX/GPP_DT_VUT_XX).

Energy and Water Flux Variables. The main variables for Latent and Sensible Heat fluxes are **LE_F_MDS** and **H_F_MDS**, respectively, both gap-filled and with quality flags. Similarly from NEE, random uncertainty from measurements are estimated (LE_RANDUNC and H_RANDUNC). If the intended use requires ensuring the energy-balance closure, a version of LE and H is provided for which the closure is enforced using the Bowen ratio method (see paper for details): **LE_CORR** and **H_CORR**. These versions also include uncertainty estimation for the half-hourly and daily time resolution (LE_CORR_25/H_CORR_25 and LE_CORR_75/H_CORR_75).

Supplementary Materials: Figures

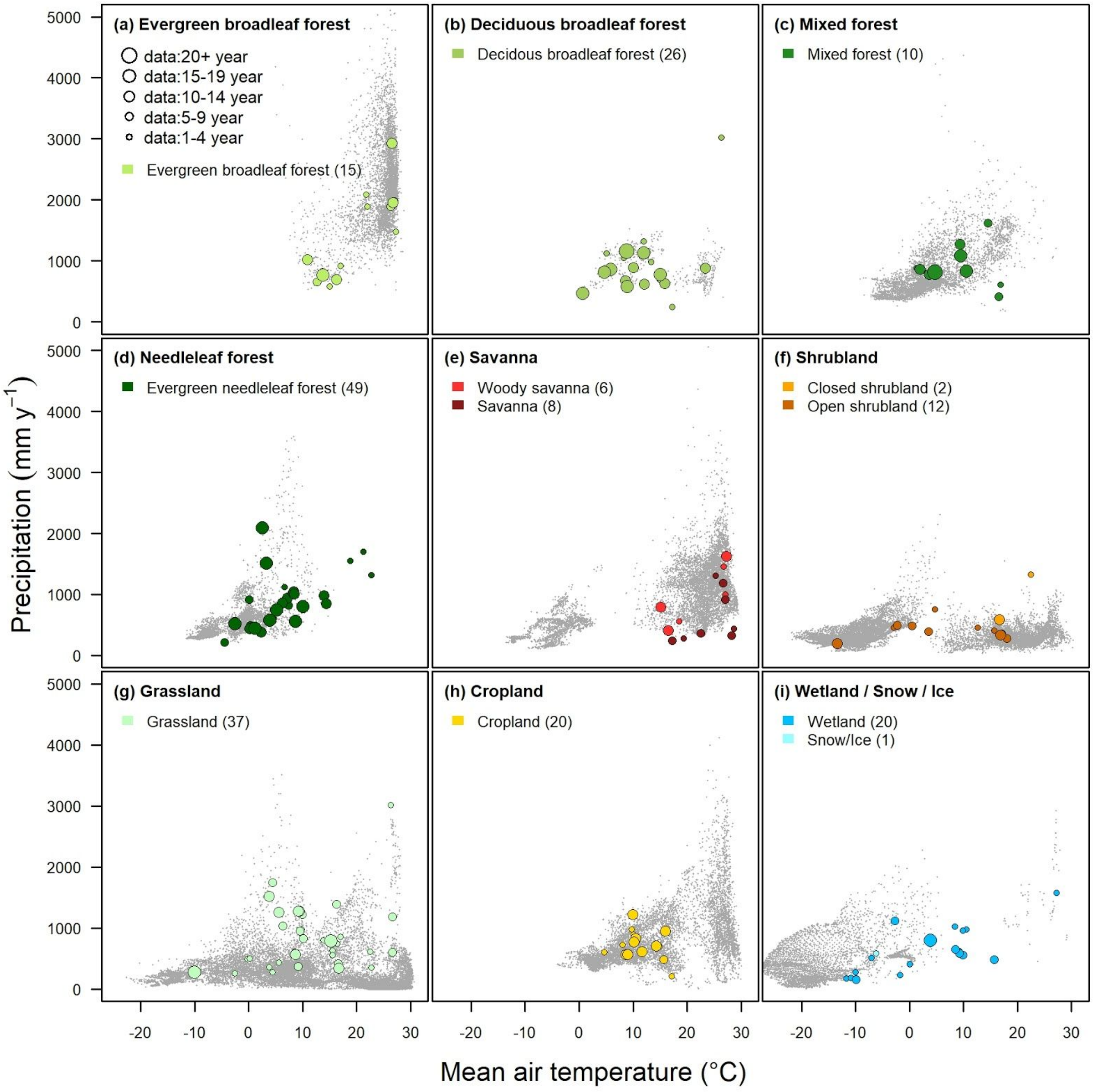

Supplementary Figure SM1. Distribution of sites by mean annual air temperature and precipitation. Tower locations are shown as circles, with vegetation type in color based on the IGBP definitions. The size of the circle indicates the length of the data record. Numbers in parentheses indicate the number of sites in each IGBP group. Gray dots represent annual mean temperature and total precipitation from the Climatic Research Unit (CRU) time series (TS) 3.23 gridded dataset from 1981 to 2014[251] and land cover types from the global mosaics of the MODIS land cover type data[252], both at 0.5° resolution.

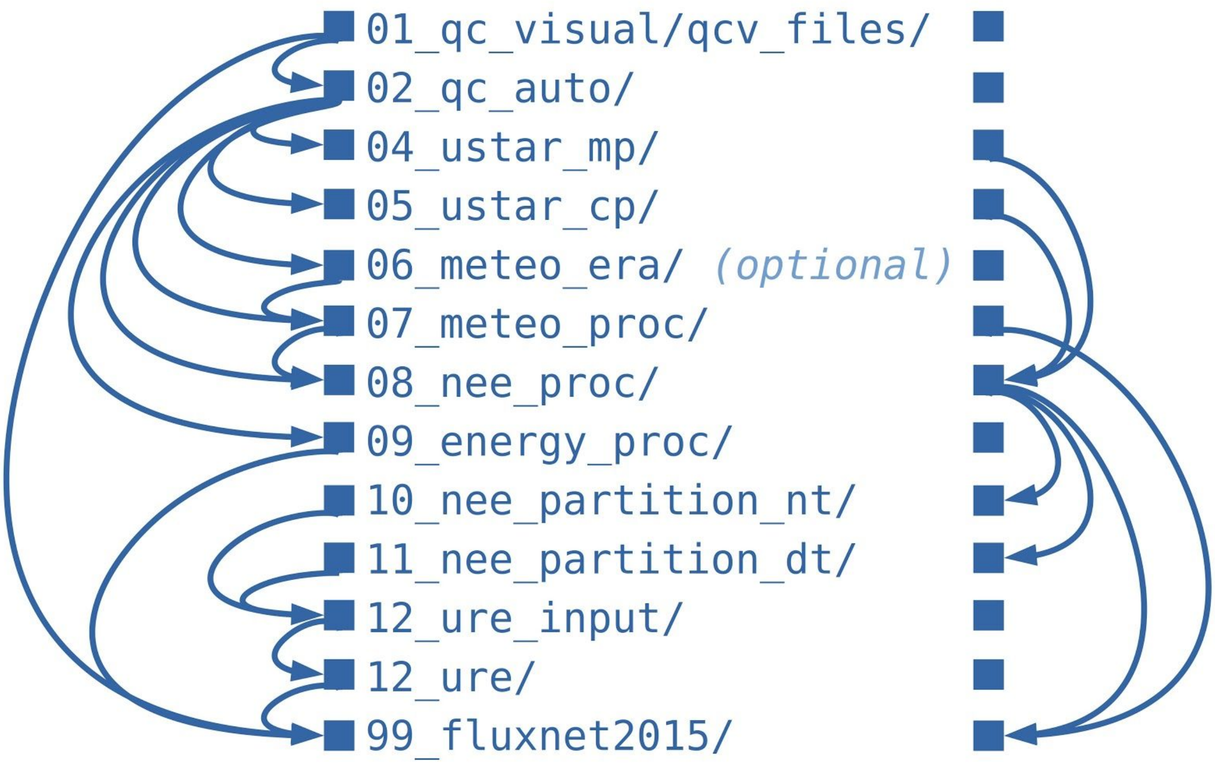

Supplementary Figure SM2. The processing steps as they are executed in the code collection package ONEFlux, with the order of execution (numbers) and their dependencies (arrows).

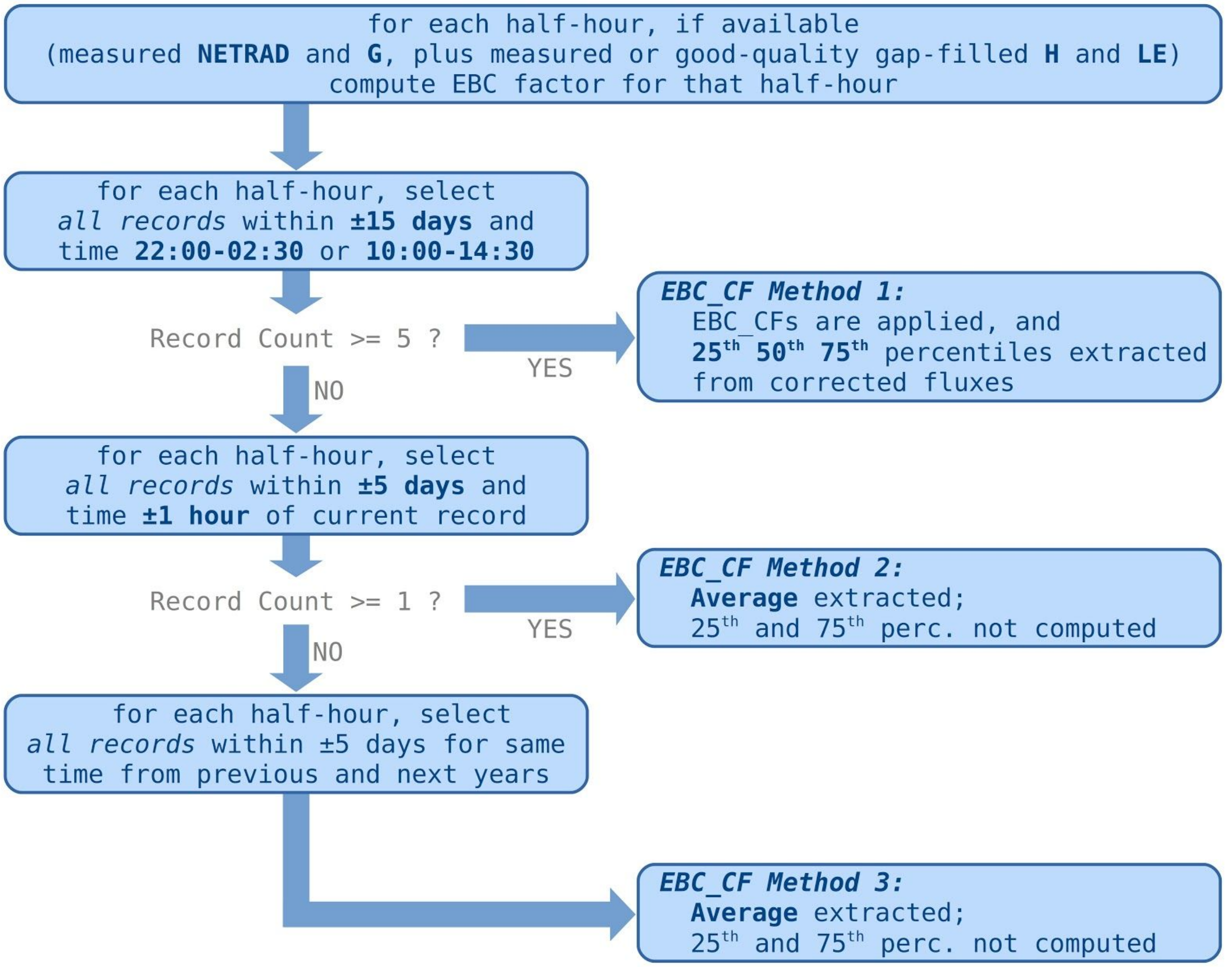

Supplementary Figure SM3. Workflow illustrating the method selection for the estimation of the correction factor (EBC_CF) to be applied to sensible (H) and latent heat (LE) fluxes, in order to ensure the energy balance closure. This figure shows the proceeding for half-hourly data. See energy and water products section in main text for more details and differences to other temporal resolutions.

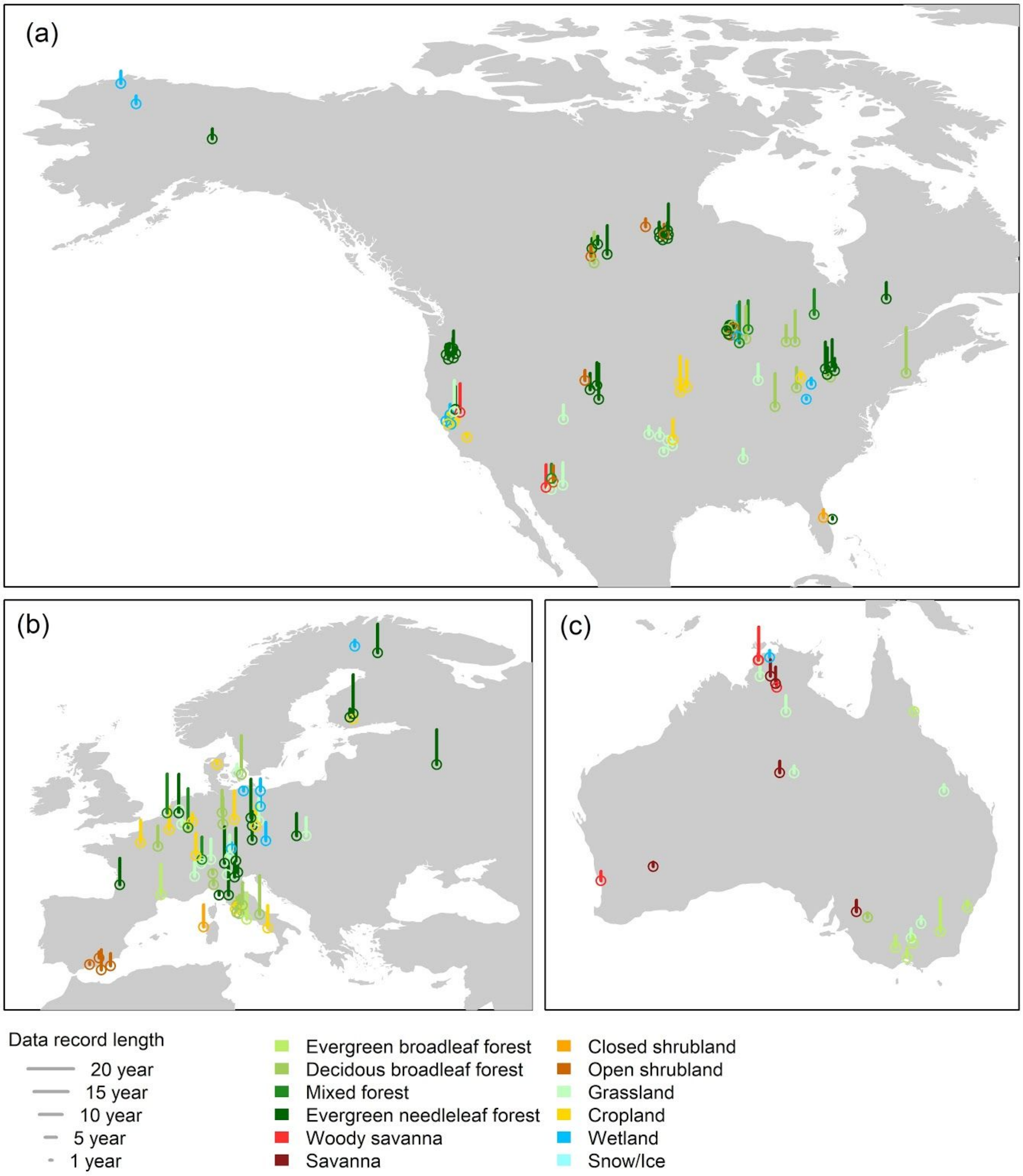

Supplementary Figure SM4. Companion figure to Fig. 1 in the main text. Continental scale maps with site positions, allowing better visualization of denser concentration of sites. Panel (a) shows upper North America, (b) Europe, and (c) Australia. As in Fig. 1, color indicates IGBP class, and here height of bar indicates length of record. When overlapping, locations are offset slightly to improve readability.

Supplementary Materials: Tables

Table SM1.
See
"***The FLUXNET2015 dataset and the ONEFlux processing pipeline for eddy covariance data—Supplementary Materials, Table SM1***"
placed at the end of the document.

Table SM2. Site General Information metadata. Required variables collected from or generated for each site are marked with an asterisk (*). Other metadata types include DOI, References, Canopy Height, and Variable Information, described in Tables S3-S6. Pre-defined options for all units are listed in Table S7. Note that all metadata files use the format YYYYMMDDHHMM for timestamps, and can be truncated at different appropriate resolutions (e.g., YYYYMMDD for a date or YYYYMM for a month).

Variable	Description / Units
ACKNOWLEDGEMENT	Acknowledgement text
ACKNOWLEDGEMENT_COMMENT	Additional information about the acknowledgement
ASPECT	Direction the site is facing (Exposure)<br>*Units: see options for ASPECT in Table S7.*
COUNTRY*	Country<br>*Units: see options for COUNTRY in Table S7.*
DOM_DIST_MGMT	Recent and historic disturbance and management events that affect the tower site years of measurement<br>*Units: see options for DOM_DIST_MGMT in Table S7.*
FLUX_MEASUREMENTS_VARIABLE	Flux variable measured<br>*Units: see options for FLUX_*MEASUREMENTS_*VARIABLE in Table S7.*
FLUX_MEASUREMENTS_METHOD	Method used to measure the flux variables<br>*Units: see options for FLUX_*MEASUREMENTS_*METHOD in Table S7.*
FLUX_MEASUREMENTS_OPERATIONS	Operational status of flux measurements<br>*Units: see options for FLUX_*MEASUREMENTS_*OPERATIONS in Table S7.*
FLUX_MEASUREMENTS_DATE_START	Date when data collection for the reported flux variable/method started<br>*Units: YYYYMMDDHHMM*
FLUX_MEASUREMENTS_DATE_END	Date when data collection for the reported flux variable/method ended<br>*Units: YYYYMMDDHHMM*
FLUX_MEASUREMENTS_COMMENT	Flux measurements comments
IGBP*	Vegetation type based on the IGBP definition

	Units: see pre-defined options for IGBP in Table S7.
IGBP_DATE_START	Date when this vegetation type first applied<br>*Units: YYYYMMDDHHMM*
IGBP_COMMENT	Vegetation type comments
LAND_OWNER	Land owner
LAND_OWNERSHIP	Land ownership type<br>*Units: see options for LAND_OWNERSHIP in Table S7.*
LOCATION_LAT*	Latitude of the site<br>*Units: decimal deg ref WGS84*
LOCATION_LONG*	Longitude of the site<br>*Units: decimal deg ref WGS84*
LOCATION_ELEV	Elevation of the site above sea level<br>*Units: m*
LOCATION_DATE_START	Begin date of the location information<br>*Units: YYYYMMDDHHMM*
LOCATION_COMMENT	Location information comments
MAP	Climatological long-term Mean Annual average Precipitation (MAP)<br>*Units: mm year-1*
MAT	Climatological long-term Mean Annual average air Temperature (MAT)<br>*Units: deg C*
NETWORK*	Network affiliation(s) of the site<br>*Units: see pre-defined options for NETWORK in Table S7.*
RESEARCH_TOPIC	Site research topics
SITE_NAME*	Site name
SITE_DESC	Short description of the site characteristics and history
SITE_FUNDING	Site funding agencies/institutions
SITE_SNOW_COVER_DAYS	Days per year that the site is covered by snow

	Units: days
SURFACE_HOMOGENEITY	Distance for which the ecosystem is homogeneous in the prevailing wind direction<br>*Units: m*
TEAM_MEMBER_NAME*	Site tower team member name (First/Given Last/Family)
TEAM_MEMBER_ROLE*	Site tower team member role<br>*Units: see options for* TEAM_MEMBER_ROLE *in Table S7.*
TEAM_MEMBER_EMAIL*	Site tower team member email
TEAM_MEMBER_INSTITUTION	Site tower team member institution
TEAM_MEMBER_ADDRESS	Site tower team member address
TERRAIN	Slope and/or relief of the site<br>*Units: see options for TERRAIN in Table S7.*
TOWER_POWER	How the eddy covariance system is powered<br>*Units: see options for TOWER_POWER in Table S7.*
TOWER_TYPE	Type of physical tower structure where the main, above canopy eddy covariance system is installed for the site<br>*Units: see options for TOWER_TYPE in Table S7.*
URL	Tower web site URL (maintained by tower team)<br>*Units: URL*
URL_FLUXNET*	URL of site page on the fluxnet.fluxdata.org<br>*Units: URL*
UTC_OFFSET*	Offset from UTC of site data<br>*Units: hours*
UTC_OFFSET_DATE_START	Begin date of the UTC offset<br>*Units: YYYYMMDDHHMM*
UTC_OFFSET_COMMENT	Offset from UTC comments
WIND_DIRECTION	Prevailing wind direction<br>*Units: see options for* WIND_DIRECTION *in Table S7.*

Table SM3. DOI metadata. Variables generated for all sites are marked with an asterisk (*).

Variable	Description / Units
DOI*	DOI for the identified flux-met data product
DOI_DATAPRODUCT	Flux-met data product associated with the DOI<br>*Units: FLUXNET2015*
DOI_CONTRIBUTOR_NAME	Name of person that contributed to the development of the data
DOI_CONTRIBUTOR_EMAIL	Email of person that contributed to the development of the data
DOI_CONTRIBUTOR_ORCID	ORCID of person that contributed to the development of the data<br>*Units: ORCID identifier*
DOI_CONTRIBUTOR_INSTITUTION	Institution of person that contributed to the development of the data
DOI_CONTRIBUTOR_ROLE	Role of person that contributed to the development of the data. Authors are listed in the DOI citation.<br>*Units: see options for* DOI_CONTRIBUTOR_ROLE *in Table S7.*
DOI_CONTRIBUTOR_ORDINAL	Listing order in the citation and DOI landing page for this contributor (1 is first)<br>*Units: integer number*
DOI_CONTRIBUTOR_DATE_START	Start date of data for which the person is a contributor<br>*Units: YYYYMMDDHHMM*
DOI_CONTRIBUTOR_DATE_END	End date of data for which the person is a contributor<br>*Units: YYYYMMDDHHMM*
DOI_ORGANIZATION	Name of the organization to be recognized for contributing to the data
DOI_ORGANIZATION_ROLE	Role of the organization to be recognized for contributing to the data<br>*Units: see options for* DOI_ORGANIZATION_ROLE *in Table S7.*

Table SM4. Publications metadata.

Variable	Description / Units
REFERENCE_PAPER	Papers relevant for understanding the site
REFERENCE_DOI	DOI of the reference
REFERENCE_USAGE	Suggested use of the reference<br>*Units: see options for* REFERENCE_USAGE *in Table S7.*
REFERENCE_COMMENT	Brief description of paper relevance or other comments

Table SM5. Canopy Height metadata. Required variables collected from all sites are marked with an asterisk (*).

Variable	Description / Units
HEIGHTC_DATE*	**Canopy height measurement date**<br>Date is reported at the precision known.<br>*Units: YYYYMMDDHHMM*
HEIGHTC*<br><br>*Note: In the case of a forest or similar ecosystems, canopy height values reported are* representative *of the distribution of overstory trees.*	**Canopy height**<br>In a forest ecosystem, canopy height is the distribution of overstory trees that see light at the top of the canopy.<br>*Units: m*

Table SM6. Variable Information metadata. Required variables collected from or generated for all sites are marked with an asterisk (*). For each site, only variables with valid data have a corresponding Variable Information group in the metadata. The earliest VAR_INFO_DATE value for each variable is the first date where observations are reported for the variable. As noted before, timestamps might be truncated to the best information available (e.g., for long running sites it might only be known the month when a sensor was installed, not the exact day and time).

Variable	Description / Units
VAR_INFO_VARNAME*	Variable name including variable qualifiers as needed.<br>*Units: Variable code. See Table S1.*
VAR_INFO_UNIT*	Variable units<br>*Units: See Table SM1. VAR_INFO_UNIT is reported for the resolution specified in the metadata file name.*
VAR_INFO_DATE*	Variable information start date<br>*Units: YYYYMMDDHHMM*
VAR_INFO_HEIGHT	Distance above or below (negative values) the ground surface at which the variable was observed<br>*Units: m*
VAR_INFO_MODEL	Instrument model(s) used for the observations reported by the variable.<br>*Units: see options for* VAR_INFO_MODEL *in Table S7.*

Table SM7.
See
"***The FLUXNET2015 dataset and the ONEFlux processing pipeline for eddy covariance data—Supplementary Materials, Table SM7***"
placed at the end of the document.

Table SM8. BADM Interchange Format (BIF) Description and Examples. All FLUXNET2015 metadata are available in files following the BADM Interchange Format (BIF), a machine-readable format for BADM. There are five BIF files distributed with FLUXNET2015 (filename: FLX_AA-Flx_BIF_<RES>_<RELEASE_DATE>.xlsx), one for each temporal resolution (<RES>: HH, DD, WW, MM, and YY). The five files follow the same format, and contain the same entries for most variables except for Variable Information metadata (Table S6), which include only variables available for each resolution and resolution specific units (see Table 2 for examples). All these five BIF files are packed in a single zip file (<RES>: ALL). The BIF format consists of five columns: (1) SITE_ID: the site FLUXNET ID; (2) GROUP_ID: a numeric identifier that is unique for all rows that should be used together (e.g., a value, the date it was collected, and the method used to collect it); (3) VARIABLE_GROUP: the label of the variable group, which can be used to separate variables by metadata types (such as those listed in Table 3); (4) VARIABLE: the label of the variable (see Tables S2-S6 for a full list of variables present in the metadata); (6) DATAVALUE: the value/measurement of the variable. Note that sites often report multiple instances of the same variable group associated with different measurements collected with different times, instruments, instrument depths, etc. The GROUP_ID uniquely identifies the data belonging to the same instance of a reported variable, and can be used to identify separate instances within the BIF file. This table shows an excerpt from the hourly (HH) BIF file for FLUXNET2015. The first three rows (with GROUP_ID 40066631) show the long wave incoming radiation variable using W m-2 as a unit and the data values for the sensor measuring this variable begin at 200402261100. Similarly, entries with GROUP_IDs of 40066659 and 40066660 describe the USTAR variable, in this case including the models and heights of the instruments, the first instrument being a SA-Gill R2, with observations starting at 199607051400, later replaced by a SA-Gill R3-50 at 200605230000. Finally, GROUP_IDs 40066736 and 40066737 show a variable (NEE_VUT_REF) which is generated by a combination of two instruments: initially GA_CP-LI-COR LI-6262 and SA-Gill R2, and after 200605230000 (when the sonic anemometer was replaced), GA_CP-LI-COR LI-6262 and SA-Gill R3-50.

SITE_ID	GROUP_ID	VARIABLE_GROUP	VARIABLE	DATAVALUE
[…]				
DE-Tha	40066631	GRP_VAR_INFO	VAR_INFO_VARNAME	LW_IN_F_MDS
DE-Tha	40066631	GRP_VAR_INFO	VAR_INFO_UNIT	W m-2
DE-Tha	40066631	GRP_VAR_INFO	VAR_INFO_DATE	200402261100
[...]				
DE-Tha	40066659	GRP_VAR_INFO	VAR_INFO_VARNAME	USTAR
DE-Tha	40066659	GRP_VAR_INFO	VAR_INFO_HEIGHT	42
DE-Tha	40066659	GRP_VAR_INFO	VAR_INFO_MODEL	SA-Gill R2

DE-Tha	40066659	GRP_VAR_INFO	VAR_INFO_UNIT	m s-1
DE-Tha	40066659	GRP_VAR_INFO	VAR_INFO_DATE	199607051400
DE-Tha	40066660	GRP_VAR_INFO	VAR_INFO_VARNAME	USTAR
DE-Tha	40066660	GRP_VAR_INFO	VAR_INFO_HEIGHT	42
DE-Tha	40066660	GRP_VAR_INFO	VAR_INFO_MODEL	SA-Gill R3-50
DE-Tha	40066660	GRP_VAR_INFO	VAR_INFO_UNIT	m s-1
DE-Tha	40066660	GRP_VAR_INFO	VAR_INFO_DATE	200605230000
[...]				
DE-Tha	40066736	GRP_VAR_INFO	VAR_INFO_VARNAME	NEE_VUT_REF
DE-Tha	40066736	GRP_VAR_INFO	VAR_INFO_HEIGHT	42
DE-Tha	40066736	GRP_VAR_INFO	VAR_INFO_MODEL	GA_CP-LI-COR LI-6262;SA-Gill R2
DE-Tha	40066736	GRP_VAR_INFO	VAR_INFO_UNIT	µmolCO2 m-2 s-1
DE-Tha	40066736	GRP_VAR_INFO	VAR_INFO_DATE	199601010000
DE-Tha	40066737	GRP_VAR_INFO	VAR_INFO_VARNAME	NEE_VUT_REF
DE-Tha	40066737	GRP_VAR_INFO	VAR_INFO_HEIGHT	42
DE-Tha	40066737	GRP_VAR_INFO	VAR_INFO_MODEL	GA_CP-LI-COR LI-6262;SA-Gill R3-50
DE-Tha	40066737	GRP_VAR_INFO	VAR_INFO_UNIT	µmolCO2 m-2 s-1
DE-Tha	40066737	GRP_VAR_INFO	VAR_INFO_DATE	200605230000
[...]				

Table SM9. Co-author list and site affiliation

Site	Co-Authors
AR-SLu	Gabriela Posse; Carlos Marcelo Di Bella; Marcelo Nosetto
AR-Vir	Gabriela Posse; Carlos Marcelo Di Bella; Marcelo Nosetto
AT-Neu	Georg Wohlfahrt; Albin Hammerle
AU-Ade	Jason Beringer; Lindsay Hutley; Ian McHugh; Matthew Northwood; Nigel Tapper; Caitlin Moore; Jeffrey P. Walker
AU-ASM	James Cleverly; Derek Eamus
AU-Cpr	Wayne Meyer; Georgia Koerber; Peter Cale
AU-Cum	Elise Pendall; Victor Resco de Dios
AU-DaP	Jason Beringer; Lindsay Hutley; Ian McHugh; Matthew Northwood; Nigel Tapper; Caitlin Moore; Jeffrey P. Walker
AU-DaS	Jason Beringer; Lindsay Hutley; Ian McHugh; Matthew Northwood; Nigel Tapper; Caitlin Moore; Jeffrey P. Walker
AU-Dry	Jason Beringer; Lindsay Hutley; Ian McHugh; Matthew Northwood; Nigel Tapper; Caitlin Moore; Jeffrey P. Walker
AU-Emr	Ivan Schroder; Andrew Feitz
AU-Fog	Jason Beringer; Lindsay Hutley; Ian McHugh; Matthew Northwood; Nigel Tapper; Caitlin Moore; Jeffrey P. Walker
AU-Gin	Richard Silberstein
AU-GWW	Craig Macfarlane; Suzanne Prober
AU-How	Jason Beringer; Lindsay Hutley; Ian McHugh; Matthew Northwood; Nigel Tapper; Caitlin Moore; Jeffrey P. Walker
AU-Lox	Cacilia M. Ewenz; Robert M. Stevens
AU-RDF	Jason Beringer; Lindsay Hutley; Ian McHugh; Matthew Northwood; Nigel Tapper; Caitlin Moore; Jeffrey P. Walker
AU-Rig	Jason Beringer; Lindsay Hutley; Ian McHugh; Matthew Northwood; Nigel Tapper; Caitlin Moore; Jeffrey P. Walker
AU-Rob	Michael Liddell
AU-Stp	Jason Beringer; Lindsay Hutley; Ian McHugh; Matthew Northwood; Nigel Tapper; Caitlin Moore; Jeffrey P. Walker
AU-TTE	James Cleverly; Derek Eamus

AU-Tum	William Woodgate; Eva van Gorsel; Ray Leuning
AU-Wac	Jason Beringer; Lindsay Hutley; Ian McHugh; Matthew Northwood; Nigel Tapper; Caitlin Moore; Jeffrey P. Walker
AU-Whr	Jason Beringer; Lindsay Hutley; Ian McHugh; Matthew Northwood; Nigel Tapper; Caitlin Moore; Jeffrey P. Walker
AU-Wom	Stefan K. Arndt; Nina Hinko-Najera; Anne Griebel
AU-Ync	Jason Beringer; Lindsay Hutley; Ian McHugh; Matthew Northwood; Nigel Tapper; Caitlin Moore; Jeffrey P. Walker
BE-Bra	Bert Gielen; Nicola Arriga; Marilyn Roland; Johan Neirynck
BE-Lon	Bernard Heinesch; Christine Moureaux; Anne De Ligne; Caroline Vincke; Marc Aubinet
BE-Vie	Bernard Heinesch; Christine Moureaux; Anne De Ligne; Caroline Vincke; Marc Aubinet
BR-Sa1	Scott R. Saleska; Natalia Restrepo-Coupe; Raimundo C. De Oliveira
BR-Sa3	Michael L. Goulden; Humberto da Rocha
CA-Gro	Harry McCaughey
CA-Man	Brian Amiro; Steve Wofsy; Allison Dunn
CA-NS1	Michael L. Goulden; Scott D. Miller; Andrew M. S. McMillan
CA-NS2	Michael L. Goulden; Scott D. Miller; Andrew M. S. McMillan
CA-NS3	Michael L. Goulden; Scott D. Miller; Andrew M. S. McMillan
CA-NS4	Michael L. Goulden; Scott D. Miller; Andrew M. S. McMillan
CA-NS5	Michael L. Goulden; Scott D. Miller; Andrew M. S. McMillan
CA-NS6	Michael L. Goulden; Scott D. Miller; Andrew M. S. McMillan
CA-NS7	Michael L. Goulden; Scott D. Miller; Andrew M. S. McMillan
CA-Oas	Thomas Andrew Black; Rachhpal Jassal; Zoran Nesic; Alan Barr
CA-Obs	Thomas Andrew Black; Rachhpal Jassal; Zoran Nesic; Alan Barr
CA-Qfo	Carole Coursolle; Onil Bergeron; Hank A. Margolis
CA-SF1	Brian Amiro; Steve Wofsy; Allison Dunn
CA-SF2	Brian Amiro; Steve Wofsy; Allison Dunn
CA-SF3	Brian Amiro; Steve Wofsy; Allison Dunn
CA-TP1	M. Altaf Arain; Jason Brodeur; Natalia Restrepo-Coupe; Matthias Peichl; Myroslava Khomik; Eric Beamesderfer
CA-TP2	M. Altaf Arain; Jason Brodeur; Natalia Restrepo-Coupe; Matthias Peichl; Myroslava Khomik; Eric Beamesderfer
CA-TP3	M. Altaf Arain; Jason Brodeur; Natalia Restrepo-Coupe; Matthias Peichl; Myroslava Khomik; Eric Beamesderfer

CA-TP4	M. Altaf Arain; Jason Brodeur; Natalia Restrepo-Coupe; Matthias Peichl; Myroslava Khomik; Eric Beamesderfer
CA-TPD	M. Altaf Arain; Jason Brodeur; Natalia Restrepo-Coupe; Matthias Peichl; Myroslava Khomik; Eric Beamesderfer
CG-Tch	Agnes de Grandcourt; Yann Nouvellon
CH-Cha	Lukas Hörtnagl; Werner Eugster; Nina Buchmann; Iris Feigenwinter
CH-Dav	Lukas Hörtnagl; Werner Eugster; Nina Buchmann; Mana Gharun
CH-Fru	Lukas Hörtnagl; Werner Eugster; Nina Buchmann
CH-Lae	Lukas Hörtnagl; Werner Eugster; Nina Buchmann; Eugenie Paul-Limoges
CH-Oe1	Christof Ammann
CH-Oe2	Lukas Hörtnagl; Werner Eugster; Nina Buchmann; Regine Maier
CN-Cha	Junhui Zhang; Shijie Han
CN-Cng	Gang Dong; Shicheng Jiang
CN-Dan	Peili Shi; Yongtao He
CN-Din	Guoyi Zhou; Yuelin Li
CN-Du2	Shiping Chen; Xingguo Han
CN-Du3	Changliang Shao; Jiquan Chen
CN-Ha2	Yingnian Li
CN-HaM	Tomomichi Kato; Yanhong Tang
CN-Qia	Huimin Wang; Xiaoqin Dai
CN-Sw2	Changliang Shao; Jiquan Chen
CZ-BK1	Jiří Dušek; Ladislav Šigut; Marian Pavelka; Dalibor Janouš; Pavel Sedlák
CZ-BK2	Jiří Dušek; Ladislav Šigut; Marian Pavelka; Dalibor Janouš; Pavel Sedlák
CZ-wet	Jiří Dušek; Ladislav Šigut; Marian Pavelka; Dalibor Janouš; Pavel Sedlák
DE-Akm	Christian Bernhofer; Thomas Grünwald; Uta Moderow; Uwe Eichelmann; Markus Hehn; Heiko Prasse
DE-Geb	Christian Brümmer; Frederik Schrader; Antje Lucas-Moffat
DE-Gri	Christian Bernhofer; Thomas Grünwald; Uta Moderow; Uwe Eichelmann; Markus Hehn; Heiko Prasse
DE-Hai	Olaf Kolle; Werner L Kutsch; Frank Tiedemann; Lukas Siebicke; Alexander Knohl
DE-Kli	Christian Bernhofer; Thomas Grünwald; Uta Moderow; Uwe Eichelmann; Markus Hehn; Heiko Prasse
DE-Lkb	Rainer Steinbrecher; Hans Peter Schmid; Janina Klatt
DE-Lnf	Olaf Kolle; Werner L Kutsch; Frank Tiedemann; Lukas Siebicke; Alexander Knohl
DE-Obe	Christian Bernhofer; Thomas Grünwald; Uta Moderow; Uwe Eichelmann; Markus Hehn; Heiko Prasse
DE-RuR	Marius Schmidt; Alexander Graf

DE-RuS	Marius Schmidt; Alexander Graf
DE-Seh	Karl Schneider; Marius Schmidt
DE-SfN	Rainer Steinbrecher; Hans Peter Schmid; Janina Klatt
DE-Spw	Christian Bernhofer; Thomas Grünwald; Uta Moderow; Uwe Eichelmann; Markus Hehn; Heiko Prasse
DE-Tha	Christian Bernhofer; Thomas Grünwald; Uta Moderow; Uwe Eichelmann; Markus Hehn; Heiko Prasse
DE-Zrk	Torsten Sachs; Christian Wille
DK-Eng	Andreas Ibrom; Kim Pilegaard
DK-Fou	Jørgen Eivind Olesen
DK-Sor	Andreas Ibrom; Kim Pilegaard
ES-Amo	Francisco Domingo; Ana López-Ballesteros; Penélope Serrano-Ortiz; Enrique P. Sánchez-Cañete
ES-LgS	Borja Ruiz; Andrew Kowalsky
ES-LJu	Francisco Domingo; Ana López-Ballesteros; Penélope Serrano-Ortiz; Enrique P. Sánchez-Cañete
ES-Ln2	Borja Ruiz; Andrew Kowalsky
FI-Hyy	Ivan Mammarella; Timo Vesala; Samuli Launiainen; Üllar Rannik
FI-Jok	Aurela Mika; Tuomas Laurila; Annalea Lohila; Juha-Pekka Tuovinen; Juha Hatakka
FI-Let	Aurela Mika; Tuomas Laurila; Annalea Lohila; Juha-Pekka Tuovinen; Juha Hatakka
FI-Lom	Aurela Mika; Tuomas Laurila; Annalea Lohila; Juha-Pekka Tuovinen; Juha Hatakka
FI-Sod	Aurela Mika; Tuomas Laurila; Annalea Lohila; Juha-Pekka Tuovinen; Juha Hatakka
FR-Fon	Daniel Berveiller; Eric Dufrêne; Nicolas Delpierre
FR-Gri	Pauline Buysse; Benjamin Loubet; Pierre Cellier
FR-LBr	Denis Loustau; Jean-Marc Bonnefond; Virginie Moreaux
FR-Pue	Jean-Marc Ourcival; Serge Rambal; Jean-Marc Limousin
GF-Guy	Damien Bonal; Benoit Burban
GH-Ank	Riccardo Valentini; Agnes de Grandcourt; Giacomo Nicolini
GL-NuF	Birger Ulf Hansen; Efrén López-Blanco
GL-ZaF	Mikhail Mastepanov; Marcin Jackowicz-Korchynski; Torben R. Christensen
GL-ZaH	Mikhail Mastepanov; Marcin Jackowicz-Korchynski; Torben R. Christensen
IT-BCi	Vincenzo Magliulo; Paul di Tommasi; Daniela Famulari
IT-CA1	Simone Sabbatini; Domenico Vitale; Michele Tomassucci; Claudia Consalvo; Nicola Arriga; Dario Papale
IT-CA2	Simone Sabbatini; Beniamino Gioli; Domenico Vitale; Michele Tomassucci; Claudia Consalvo; Nicola Arriga; Dario Papale

IT-CA3	Simone Sabbatini; Domenico Vitale; Michele Tomassucci; Claudia Consalvo; Nicola Arriga; Dario Papale
IT-Col	Francesco Mazzenga; Ettore D'Andrea; Bruno De Cinti; Alessio Collalti; Giovanni Manca; Riccardo Valentini
IT-Cp2	Silvano Fares
IT-Cpz	Simone Sabbatini; Domenico Vitale; Michele Tomassucci; Sabina Dore; Riccardo Valentini; Dario Papale; Francesco Mazzenga
IT-Isp	Ignacio Goded; Carsten Gruening
IT-La2	Damiano Gianelle; Isaac Chini; Barbara Marcolla; Roberto Zampedri; Mauro Cavagna
IT-Lav	Damiano Gianelle; Isaac Chini; Barbara Marcolla; Roberto Zampedri; Mauro Cavagna
IT-MBo	Damiano Gianelle; Isaac Chini; Barbara Marcolla; Roberto Zampedri; Mauro Cavagna
IT-Noe	Donatella Spano; Pierpaolo Duce; Costantino Sirca; Serena Marras
IT-PT1	Giovanni Manca; Ignacio Goded; Carsten Gruening
IT-Ren	Leonardo Montagnani; Stefano Minerbi
IT-Ro1	Simone Sabbatini; Domenico Vitale; Michele Tomassucci; Claudia Consalvo; Sabina Dore; Luca Belelli Marchesini; Nicola Arriga; Giovanni Manca; Riccardo Valentini; Dario Papale; Francesco Mazzenga
IT-Ro2	Simone Sabbatini; Domenico Vitale; Michele Tomassucci; Claudia Consalvo; Sabina Dore; Luca Belelli Marchesini; Nicola Arriga; Giovanni Manca; Riccardo Valentini; Dario Papale; Francesco Mazzenga
IT-SR2	Ignacio Goded; Carsten Gruening
IT-SRo	Giovanni Manca; Ignacio Goded; Carsten Gruening
IT-Tor	Edoardo Cremonese; Marta Galvagno; Gianluca Filippa
JP-MBF	Ayumi Kotani; Taro Nakai
JP-SMF	Ayumi Kotani; Taro Nakai
MY-PSO	Satoru Takanashi; Yoshiko Kosugi; Marryanna Lion
NL-Hor	Luca Belelli Marchesini; Han Dolman; Ko van Huissteden
NL-Loo	Eddy Moors; Wilma Jans; Michiel van der Molen; Bart Kruijt
PA-SPn	Sebastian Wolf; Werner Eugster
PA-SPs	Sebastian Wolf; Werner Eugster
RU-Che	Mathias Goeckede; Lutz Merbold
RU-Cok	Luca Belelli Marchesini; Han Dolman; Ko van Huissteden
RU-Fyo	Natalia Vygodskaya; Ivan Shironya; Julia Kurbatova; Andrej Varlagin
RU-Ha1	Luca Belelli Marchesini; Dario Papale, Riccardo Valentini

SD-Dem	Jonas Ardö; Hatim Abdalla M. ElKhidir
SJ-Adv	Frans-Jan Parmentier; Norbert Pirk
SJ-Blv	Julia Boike; Sebastian Westermann; Johannes Lüers
SN-Dhr	Torbern Tagesson; Rasmus Fensholt
US-AR1	Margaret Torn; Dave Billesbach; Naama Raz-Yaseef
US-AR2	Margaret Torn; Dave Billesbach; Naama Raz-Yaseef
US-ARb	Margaret Torn; Dave Billesbach; Naama Raz-Yaseef
US-ARc	Margaret Torn; Dave Billesbach; Naama Raz-Yaseef
US-ARM	Sebastien Biraud; Marc Fischer; Margaret Torn
US-Atq	Donatella Zona; Oechel Walter
US-Blo	Allen H Goldstein; Silvano Fares; Robin Weber
US-Cop	David Bowling
US-CRT	Jiquan Chen; Asko Noormets; Housen Chu
US-GBT	William Massman; John Frank
US-GLE	William Massman; John Frank
US-Goo	Tilden Meyers
US-Ha1	J William Munger; Michael L. Goulden; Shawn Urbanski; Steven Wofsy
US-IB2	Roser Matamala; David Cook
US-Ivo	Donatella Zona; Oechel Walter
US-KS1	Powell Thomas; Bracho Rosvel
US-KS2	Powell Thomas; Bracho Rosvel
US-Lin	Allen H Goldstein; Silvano Fares; Robin Weber
US-Los	Ankur R. Desai; Kenneth J. Davis; Paul V. Bolstad; Bruce D. Cook; Jonathan Thom
US-LWW	Tilden Meyers
US-Me1	Bev Law; Hyojung Kwon; Chad Hanson
US-Me2	Bev Law; Hyojung Kwon; Chad Hanson
US-Me3	Bev Law; Hyojung Kwon; Chad Hanson
US-Me4	Bev Law; Hyojung Kwon; Chad Hanson
US-Me5	Bev Law; Hyojung Kwon; Chad Hanson
US-Me6	Bev Law; Hyojung Kwon; Chad Hanson
US-MMS	Kimberly Novick; Richard Phillips
US-Myb	Siyan Ma; Jaclyn Hatala Matthes; Sara Knox; Cove Sturtevant; Joseph Verfaillie; Dennis Baldocchi
US-Ne1	Andy Suyker; Timothy Arkebauer; Elizabeth Walter-Shea; Adam Liska; Anatoly Gitelson
US-Ne2	Andy Suyker; Timothy Arkebauer; Elizabeth Walter-Shea; Adam Liska; Anatoly Gitelson

US-Ne3	Andy Suyker; Timothy Arkebauer; Elizabeth Walter-Shea; Adam Liska; Anatoly Gitelson
US-NR1	Peter D. Blanken; Russell K. Monson; Sean P. Burns; David R. Bowling
US-Oho	Jiquan Chen; Asko Noormets; Housen Chu
US-ORv	Gil Bohrer
US-PFa	Ankur R. Desai; Kenneth J. Davis; Paul V. Bolstad; Bruce D. Cook; Jonathan Thom; Arlyn E. Andrews
US-Prr	Hiroki Ikawa; Hideki Kobayashi
US-SRC	Shirley A. Papuga; Zulia M. Sanchez-Mejia
US-SRG	Russell L. Scott
US-SRM	Russell L. Scott
US-Sta	Brent Ewers; David Reed
US-Syv	Ankur R. Desai; Kenneth J. Davis; Paul V. Bolstad; Bruce D. Cook; Jonathan Thom
US-Ton	Siyan Ma; Jaclyn Hatala Matthes; Sara Knox; Cove Sturtevant; Joseph Verfaillie; Dennis Baldocchi
US-Tw1	Siyan Ma; Jaclyn Hatala Matthes; Sara Knox; Cove Sturtevant; Joseph Verfaillie; Dennis Baldocchi
US-Tw2	Siyan Ma; Jaclyn Hatala Matthes; Sara Knox; Cove Sturtevant; Joseph Verfaillie; Dennis Baldocchi
US-Tw3	Siyan Ma; Jaclyn Hatala Matthes; Sara Knox; Cove Sturtevant; Joseph Verfaillie; Dennis Baldocchi
US-Tw4	Siyan Ma; Jaclyn Hatala Matthes; Sara Knox; Cove Sturtevant; Joseph Verfaillie; Dennis Baldocchi
US-Twt	Siyan Ma; Jaclyn Hatala Matthes; Sara Knox; Cove Sturtevant; Joseph Verfaillie; Dennis Baldocchi
US-UMB	Gil Bohrer; Christopher M. Gough; Peter S. Curtis
US-UMd	Gil Bohrer; Christopher M. Gough; Peter S. Curtis
US-Var	Siyan Ma; Jaclyn Hatala Matthes; Sara Knox; Cove Sturtevant; Joseph Verfaillie; Dennis Baldocchi
US-WCr	Ankur R. Desai; Kenneth J. Davis; Paul V. Bolstad; Bruce D. Cook; Jonathan Thom
US-Whs	Russell L. Scott
US-Wi0	Jiquan Chen; Asko Noormets; Housen Chu
US-Wi1	Jiquan Chen; Asko Noormets; Housen Chu
US-Wi2	Jiquan Chen; Asko Noormets; Housen Chu
US-Wi3	Jiquan Chen; Asko Noormets; Housen Chu
US-Wi4	Jiquan Chen; Asko Noormets; Housen Chu
US-Wi5	Jiquan Chen; Asko Noormets; Housen Chu

US-Wi6	Jiquan Chen; Asko Noormets; Housen Chu
US-Wi7	Jiquan Chen; Asko Noormets; Housen Chu
US-Wi8	Jiquan Chen; Asko Noormets; Housen Chu
US-Wi9	Jiquan Chen; Asko Noormets; Housen Chu
US-Wkg	Russell L. Scott
US-WPT	Jiquan Chen; Asko Noormets; Housen Chu
ZM-Mon	Werner L Kutsch; Lutz Merbold

Table SM1. Complete variables list for FLUXNET2015 dataset

Variable	Units	Description
TIMEKEEPING		
TIMESTAMP	YYYYMMDDHHMM	ISO timestamp - short format
TIMESTAMP_START	YYYYMMDDHHMM	ISO timestamp start of averaging period - short format
TIMESTAMP_END	YYYYMMDDHHMM	ISO timestamp end of averaging period - short format
METEOROLOGICAL		
TA_F_MDS		Air temperature, gapfilled using MDS method
HH	deg C	
DD	deg C	average from half-hourly data
WW-YY	deg C	average from daily data
TA_F_MDS_QC		Quality flag for TA_F_MDS
HH	nondimensional	0 = measured; 1 = good quality gapfill; 2 = medium; 3 = poor
DD	nondimensional	fraction between 0-1, indicating percentage of measured and good quality gapfill data
WW-YY	nondimensional	fraction between 0-1, indicating percentage of measured and good quality gapfill data (average from daily data)
TA_F_MDS_NIGHT		Average nighttime TA_F_MDS
HH		not available
DD	deg C	average from half-hourly data
WW-YY	deg C	average from daily data
TA_F_MDS_NIGHT_SD		Standard deviation for TA_F_MDS_NIGHT
HH		not available
DD	deg C	from half-hourly data
WW-YY	deg C	average SD from daily data
TA_F_MDS_NIGHT_QC		Quality flag for TA_F_MDS_NIGHT
HH		not available
DD	nondimensional	fraction between 0-1, indicating percentage of measured and good quality gapfill data

WW-YY	nondimensional	fraction between 0-1, indicating percentage of measured and good quality gapfill data (average from daily data)
TA_F_MDS_DAY		Average daytime TA_F_MDS
HH		not available
DD	deg C	average from half-hourly data
WW-YY	deg C	average from daily data
TA_F_MDS_DAY_SD		Standard deviation for TA_F_MDS_DAY
HH		not available
DD	deg C	from half-hourly data
WW-YY	deg C	average SD from daily data
TA_F_MDS_DAY_QC		Quality flag for TA_F_MDS_DAY
HH		not available
DD	nondimensional	fraction between 0-1, indicating percentage of measured and good quality gapfill data
WW-YY	nondimensional	fraction between 0-1, indicating percentage of measured and good quality gapfill data (average from daily data)
TA_ERA		Air temperature, downscaled from ERA, linearly regressed using measured only site data
HH	deg C	
DD	deg C	average from half-hourly data
WW-YY	deg C	average from daily data
TA_ERA_NIGHT		Average nighttime TA_ERA
HH		not available
DD	deg C	average from half-hourly data
WW-YY	deg C	average from daily data
TA_ERA_NIGHT_SD		Standard deviation for TA_ERA_NIGHT
HH		not available
DD	deg C	from half-hourly data
WW-YY	deg C	average SD from daily data
TA_ERA_DAY		Average daytime TA_ERA
HH		not available
DD	deg C	average from half-hourly data
WW-YY	deg C	average from daily data

TA_ERA_DAY_SD		Standard deviation for TA_ERA_DAY
HH		not available
DD	deg C	from half-hourly data
WW-YY	deg C	average SD from daily data
TA_F		Air temperature, consolidated from TA_F_MDS and TA_ERA
HH	deg C	TA_F_MDS used if TA_F_MDS_QC is 0 or 1
DD	deg C	average from half-hourly data
WW-YY	deg C	average from daily data
TA_F_QC		Quality flag for TA_F
HH	nondimensional	0 = measured; 1 = good quality gapfill; 2 = downscaled from ERA
DD	nondimensional	fraction between 0-1, indicating percentage of measured and good quality gapfill data
WW-YY	nondimensional	fraction between 0-1, indicating percentage of measured and good quality gapfill data (average from daily data)
TA_F_NIGHT		Average nighttime TA_F
HH		not available
DD	deg C	average from half-hourly data
WW-YY	deg C	average from daily data
TA_F_NIGHT_SD		Standard deviation for TA_F_NIGHT
HH		not available
DD	deg C	from half-hourly data
WW-YY	deg C	average SD from daily data
TA_F_NIGHT_QC		Quality flag for TA_F_NIGHT
HH		not available
DD	nondimensional	fraction between 0-1, indicating percentage of measured and good quality gapfill data
WW-YY	nondimensional	fraction between 0-1, indicating percentage of measured and good quality gapfill data (average from daily data)
TA_F_DAY		Average daytime TA_F
HH		not available
DD	deg C	average from half-hourly data
WW-YY	deg C	average from daily data

TA_F_DAY_SD		Standard deviation for TA_F_DAY
HH		not available
DD	deg C	from half-hourly data
WW-YY	deg C	average SD from daily data
TA_F_DAY_QC		Quality flag for TA_F_DAY
HH		not available
DD	nondimensional	fraction between 0-1, indicating percentage of measured and good quality gapfill data
WW-YY	nondimensional	fraction between 0-1, indicating percentage of measured and good quality gapfill data (average from daily data)
SW_IN_POT		Shortwave radiation, incoming, potential (top of atmosphere)
HH	W m-2	
DD	W m-2	average from half-hourly data
WW-MM	W m-2	average from daily data
YY		not available
SW_IN_F_MDS		Shortwave radiation, incoming, gapfilled using MDS (negative values set to zero, e.g., negative values from instrumentation noise)
HH	W m-2	
DD	W m-2	average from half-hourly data
WW-YY	W m-2	average from daily data
SW_IN_F_MDS_QC		Quality flag for SW_IN_F_MDS
HH	nondimensional	0 = measured; 1 = good quality gapfill; 2 = medium; 3 = poor
DD	nondimensional	fraction between 0-1, indicating percentage of measured and good quality gapfill data
WW-YY	nondimensional	fraction between 0-1, indicating percentage of measured and good quality gapfill data (average from daily data)
SW_IN_ERA		Shortwave radiation, incoming, downscaled from ERA, linearly regressed using measured only site data (negative values set to zero)
HH	W m-2	
DD	W m-2	average from half-hourly data

WW-YY	W m-2	average from daily data
SW_IN_F		Shortwave radiation, incoming consolidated from SW_IN_F_MDS and SW_IN_ERA (negative values set to zero)
HH	W m-2	SW_IN_F_MDS used if SW_IN_F_MDS_QC is 0 or 1
DD	W m-2	average from half-hourly data
WW-YY	W m-2	average from daily data
SW_IN_F_QC		Quality flag for SW_IN_F
HH	nondimensional	0 = measured; 1 = good quality gapfill; 2 = downscaled from ERA
DD	nondimensional	fraction between 0-1, indicating percentage of measured and good quality gapfill data
WW-YY	nondimensional	fraction between 0-1, indicating percentage of measured and good quality gapfill data (average from daily data)
LW_IN_F_MDS		Longwave radiation, incoming, gapfilled using MDS
HH	W m-2	
DD	W m-2	average from half-hourly data
WW-YY	W m-2	average from daily data
LW_IN_F_MDS_QC		Quality flag for LW_IN_F_MDS
HH	nondimensional	0 = measured; 1 = good quality gapfill; 2 = medium; 3 = poor
DD	nondimensional	fraction between 0-1, indicating percentage of measured and good quality gapfill data
WW-YY	nondimensional	fraction between 0-1, indicating percentage of measured and good quality gapfill data (average from daily data)
LW_IN_ERA		Longwave radiation, incoming, downscaled from ERA, linearly regressed using measured only site data
HH	W m-2	
DD	W m-2	average from half-hourly data
WW-YY	W m-2	average from daily data
LW_IN_F		Longwave radiation, incoming, consolidated from LW_IN_F_MDS and LW_IN_ERA

HH	W m-2	LW_IN_F_MDS used if LW_IN_F_MDS_QC is 0 or 1
DD	W m-2	average from half-hourly data
WW-YY	W m-2	average from daily data
LW_IN_F_QC		Quality flag for LW_IN_F
HH	nondimensional	0 = measured; 1 = good quality gapfill; 2 = downscaled from ERA
DD	nondimensional	fraction between 0-1, indicating percentage of measured and good quality gapfill data
WW-YY	nondimensional	fraction between 0-1, indicating percentage of measured and good quality gapfill data (average from daily data)
LW_IN_JSB		Longwave radiation, incoming, calculated from TA_F_MDS, SW_IN_F_MDS, VPD_F_MDS and SW_IN_POT using the JSBACH algorithm (Sonke Zaehle)
HH	W m-2	
DD	W m-2	average from half-hourly data
WW-YY	W m-2	average from daily data
LW_IN_JSB_QC		Quality flag for LW_IN_JSB
HH	nondimensional	highest from TA_F_MDS_QC, SW_IN_F_MDS_QC, and VPD_F_MDS_QC, poorest quality prevails
DD	nondimensional	fraction between 0-1, indicating percentage of calculated LW_IN starting from measured and good quality gapfill drivers data
WW-YY	nondimensional	fraction between 0-1, indicating percentage of calculated LW_IN starting from measured and good quality gapfill drivers data (average from daily data)
LW_IN_JSB_ERA		Longwave radiation, incoming, downscaled from ERA, linearly regressed using site level LW_IN_JSB calculated from measured only drivers
HH	W m-2	
DD	W m-2	average from half-hourly data
WW-YY	W m-2	average from daily data
LW_IN_JSB_F		Longwave radiation, incoming, consolidated from LW_IN_JSB and LW_IN_JSB_ERA

HH	W m-2	LW_IN_JSB used if LW_IN_JSB_QC is 0 or 1
DD	W m-2	average from half-hourly data
WW-YY	W m-2	average from daily data
LW_IN_JSB_F_QC		Quality flag for LW_IN_JSB_F
HH	nondimensional	0 = calculated from measured drivers; 1 = calculated from good quality gapfilled drivers; 2: downscaled from ERA
DD	nondimensional	fraction between 0-1, indicating percentage of measured and good quality gapfill data
WW-YY	nondimensional	fraction between 0-1, indicating percentage of measured and good quality gapfill data (average from daily data)
VPD_F_MDS		Vapor Pressure Deficit, gapfilled using MDS
HH	hPa	
DD	hPa	average from half-hourly data
WW-YY	hPa	average from daily data
VPD_F_MDS_QC		Quality flag for VPD_F_MDS
HH	nondimensional	0 = measured; 1 = good quality gapfill; 2 = medium; 3 = poor
DD	nondimensional	fraction between 0-1, indicating percentage of measured and good quality gapfill data
WW-YY	nondimensional	fraction between 0-1, indicating percentage of measured and good quality gapfill data (average from daily data)
VPD_ERA		Vapor Pressure Deficit, downscaled from ERA, linearly regressed using measured only site data
HH	hPa	
DD	hPa	average from half-hourly data
WW-YY	hPa	average from daily data
VPD_F		Vapor Pressure Deficit consolidated from VPD_F_MDS and VPD_ERA
HH	hPa	VPD_F_MDS used if VPD_F_MDS_QC is 0 or 1
DD	hPa	average from half-hourly data
WW-YY	hPa	average from daily data
VPD_F_QC		Quality flag for VPD_F

HH	nondimensional	0 = measured; 1 = good quality gapfill; 2 = downscaled from ERA
DD	nondimensional	fraction between 0-1, indicating percentage of measured and good quality gapfill data
WW-YY	nondimensional	fraction between 0-1, indicating percentage of measured and good quality gapfill data (average from daily data)
PA		Atmospheric pressure
HH	kPa	
DD-YY		not available
PA_ERA		Atmospheric pressure, downscaled from ERA, linearly regressed using measured only site data
HH	kPa	
DD	kPa	average from half-hourly data
WW-YY	kPa	average from daily data
PA_F		Atmospheric pressure consolidated from PA and PA_ERA
HH	kPa	PA used if measured
DD	kPa	average from half-hourly data
WW-YY	kPa	average from daily data
PA_F_QC		Quality flag for PA_F
HH	nondimensional	0 = measured; 2 = downscaled from ERA
DD	nondimensional	fraction between 0-1, indicating percentage of measured data
WW-YY	nondimensional	fraction between 0-1, indicating percentage of measured data (average from daily data)
P		Precipitation
HH	mm	
DD-YY		not available
P_ERA		Precipitation, downscaled from ERA, linearly regressed using measured only site data
HH	mm	(mm per dataset resolution: either hour or half-hour)
DD	mm d-1	sum from half-hourly data (mm per day)
WW-MM	mm d-1	average from daily data (mm per day)
YY	mm y-1	sum from daily data (mm per year)

P_F		Precipitation consolidated from P and P_ERA
HH	mm	P used if measured (mm per dataset resolution: either hour or half-hour)
DD	mm d-1	sum from half-hourly data (mm per day)
WW-MM	mm d-1	average from daily data (mm per day)
YY	mm y-1	sum from daily data (mm per year)
P_F_QC		Quality flag for P_F
HH	nondimensional	0 = measured; 2 = downscaled from ERA
DD	nondimensional	fraction between 0-1, indicating percentage of measured data
WW-YY	nondimensional	fraction between 0-1, indicating percentage of measured data (average from daily data)
WS		Wind speed
HH	m s-1	
DD-YY		not available
WS_ERA		Wind speed, downscaled from ERA, linearly regressed using measured only site data
HH	m s-1	
DD	m s-1	average from half-hourly data
WW-YY	m s-1	average from daily data
WS_F		Wind speed, consolidated from WS and WS_ERA
HH	m s-1	WS used if measured
DD	m s-1	average from half-hourly data
WW-YY	m s-1	average from daily data
WS_F_QC		Quality flag of WS_F
HH	nondimensional	0 = measured; 2 = downscaled from ERA
DD	nondimensional	fraction between 0-1, indicating percentage of measured data
WW-YY	nondimensional	fraction between 0-1, indicating percentage of measured data (average from daily data)
WD		Wind direction
HH	Decimal degrees	
DD-YY		not available
RH		Relative humidity, range 0-100

HH	%	
DD-YY		not available
USTAR		Friction velocity
HH	m s-1	
DD	m s-1	average from half-hourly data (only days with more than 50% records available)
WW-YY	m s-1	average from daily data (only periods with more than 50% records available)
USTAR_QC		Quality flag of USTAR
HH		not available
DD	nondimensional	fraction between 0-1, indicating percentage of data available (measured)
WW-YY	nondimensional	fraction between 0-1, indicating percentage of data available (average from daily data)
NETRAD		Net radiation
HH	W m-2	
DD	W m-2	average from half-hourly data (only days with more than 50% records available)
WW-YY	W m-2	average from daily data (only periods with more than 50% records available)
NETRAD_QC		Quality flag of NETRAD
HH		not available
DD	nondimensional	fraction between 0-1, indicating percentage of data available (measured)
WW-YY	nondimensional	fraction between 0-1, indicating percentage of data available (average from daily data)
PPFD_IN		Photosynthetic photon flux density, incoming
HH	µmolPhoton m-2 s-1	
DD	µmolPhoton m-2 s-1	average from half-hourly data (only days with more than 50% records available)
WW-YY	µmolPhoton m-2 s-1	average from daily data (only periods with more than 50% records available)
PPFD_IN_QC		Quality flag of PPFD_IN
HH		not available
DD	nondimensional	fraction between 0-1, indicating percentage of data available (measured)

WW-YY	nondimensional	fraction between 0-1, indicating percentage of data available (average from daily data)
PPFD_DIF		Photosynthetic photon flux density, diffuse incoming
HH	µmolPhoton m-2 s-1	
DD	µmolPhoton m-2 s-1	average from half-hourly data (only days with more than 50% records available)
WW-YY	µmolPhoton m-2 s-1	average from daily data (only periods with more than 50% records available)
PPFD_DIF_QC		Quality flag of PPFD_DIF
HH		not available
DD	nondimensional	fraction between 0-1, indicating percentage of data available (measured)
WW-YY	nondimensional	fraction between 0-1, indicating percentage of data available (average from daily data)
PPFD_OUT		Photosynthetic photon flux density, outgoing
HH	µmolPhoton m-2 s-1	
DD	µmolPhoton m-2 s-1	average from half-hourly data (only days with more than 50% records available)
WW-YY	µmolPhoton m-2 s-1	average from daily data (only periods with more than 50% records available)
PPFD_OUT_QC		Quality flag of PPFD_OUT
HH		not available
DD	nondimensional	fraction between 0-1, indicating percentage of data available (measured)
WW-YY	nondimensional	fraction between 0-1, indicating percentage of data available (average from daily data)
SW_DIF		Shortwave radiation, diffuse incoming
HH	W m-2	
DD	W m-2	average from half-hourly data (only days with more than 50% records available)
WW-YY	W m-2	average from daily data (only periods with more than 50% records available)
SW_DIF_QC		Quality flag of SW_DIF
HH		not available

DD	nondimensional	fraction between 0-1, indicating percentage of data available (measured)
WW-YY	nondimensional	fraction between 0-1, indicating percentage of data available (average from daily data)
SW_OUT		Shortwave radiation, outgoing
HH	W m-2	
DD	W m-2	average from half-hourly data (only days with more than 50% records available)
WW-YY	W m-2	average from daily data (only periods with more than 50% records available)
SW_OUT_QC		Quality flag of SW_OUT
HH		not available
DD	nondimensional	fraction between 0-1, indicating percentage of data available (measured)
WW-YY	nondimensional	fraction between 0-1, indicating percentage of data available (average from daily data)
LW_OUT		Longwave radiation, outgoing
HH	W m-2	
DD	W m-2	average from half-hourly data (only days with more than 50% records available)
WW-YY	W m-2	average from daily data (only periods with more than 50% records available)
LW_OUT_QC		Quality flag of LW_OUT
HH		not available
DD	nondimensional	fraction between 0-1, indicating percentage of data available (measured)
WW-YY	nondimensional	fraction between 0-1, indicating percentage of data available (average from daily data)
CO2_F_MDS		CO2 mole fraction, gapfilled with MDS
HH	µmolCO2 mol-1	
DD	µmolCO2 mol-1	average from half-hourly data
WW-YY	µmolCO2 mol-1	average from daily data
CO2_F_MDS_QC		Quality flag for CO2_F_MDS
HH	nondimensional	0 = measured; 1 = good quality gapfill; 2 = medium; 3 = poor
DD	nondimensional	fraction between 0-1, indicating percentage of measured and good quality gapfill data

WW-YY	nondimensional	fraction between 0-1, indicating percentage of measured and good quality gapfill data (average from daily data)
TS_F_MDS_#		Soil temperature, gapfilled with MDS (numeric index "#" increases with the depth, 1 is shallowest)
HH	deg C	
DD	deg C	average from half-hourly data
WW-YY	deg C	average from daily data
TS_F_MDS_#_QC		Quality flag for TS_F_MDS_#
HH	nondimensional	0 = measured; 1 = good quality gapfill; 2 = medium; 3 = poor
DD	nondimensional	fraction between 0-1, indicating percentage of measured and good quality gapfill data
WW-YY	nondimensional	fraction between 0-1, indicating percentage of measured and good quality gapfill data (average from daily data)
SWC_F_MDS_#		Soil water content, gapfilled with MDS (numeric index "#" increases with the depth, 1 is shallowest)
HH	%	
DD	%	average from half-hourly data
WW-YY	%	average from daily data
SWC_F_MDS_#_QC		Quality flag for SWC_F_MDS_#
HH	nondimensional	0 = measured; 1 = good quality gapfill; 2 = medium; 3 = poor
DD	nondimensional	fraction between 0-1, indicating percentage of measured and good quality gapfill data
WW-YY	nondimensional	fraction between 0-1, indicating percentage of measured and good quality gapfill data (average from daily data)
ENERGY PROCESSING		
G_F_MDS		Soil heat flux
HH	W m-2	
DD	W m-2	average from half-hourly data
WW-YY	W m-2	average from daily data
G_F_MDS_QC		Quality flag of G_F_MDS

HH	nondimensional	0 = measured; 1 = good quality gapfill; 2 = medium; 3 = poor
DD	nondimensional	fraction between 0-1, indicating percentage of measured and good quality gapfill data
WW-YY	nondimensional	fraction between 0-1, indicating percentage of measured and good quality gapfill data (average from daily data)
LE_F_MDS		Latent heat flux, gapfilled using MDS method
HH	W m-2	
DD	W m-2	average from half-hourly data
WW-YY	W m-2	average from daily data
LE_F_MDS_QC		Quality flag for LE_F_MDS, LE_CORR, LE_CORR25, and LE_CORR75.
HH	nondimensional	0 = measured; 1 = good quality gapfill; 2 = medium; 3 = poor
DD	nondimensional	fraction between 0-1, indicating percentage of measured and good quality gapfill data
WW-YY	nondimensional	fraction between 0-1, indicating percentage of measured and good quality gapfill data (average from daily data)
LE_CORR		Latent heat flux, corrected LE_F_MDS by energy balance closure correction factor
HH	W m-2	
DD	W m-2	average from half-hourly data
WW-YY	W m-2	average from daily data
LE_CORR_25		Latent heat flux, corrected LE_F_MDS by energy balance closure correction factor, 25th percentile
HH	W m-2	
DD	W m-2	average from half-hourly data
WW-YY		not available
LE_CORR_75		Latent heat flux, corrected LE_F_MDS by energy balance closure correction factor, 75th percentile
HH	W m-2	
DD	W m-2	average from half-hourly data
WW-YY		not available

LE_RANDUNC		Random uncertainty of LE, from measured only data
HH	W m-2	uses only data point where LE_F_MDS_QC is 0 and two hierarchical methods (see header and LE_RANDUNC_METHOD)
DD-YY	W m-2	from random uncertainty of individual half-hours (rand(i)) = [SQRT(SUM(rand(i)^2)) / n], where n is the number of half-hours used
LE_RANDUNC_METHOD		Method used to estimate the random uncertainty of LE
HH	nondimensional	1 = RANDUNC Method 1 (direct SD method), 2 = RANDUNC Method 2 (median SD method)
DD-YY		not available
LE_RANDUNC_N		Number of half-hour data points used to estimate the random uncertainty of LE
HH	nondimensional	
DD-YY		not available
LE_CORR_JOINTUNC		Joint uncertainty estimation for LE
HH-DD	W m-2	[SQRT(LE_RANDUNC^2 + ((LE_CORR75 - LE_CORR25) / 1.349)^2)]
WW-YY		not available
H_F_MDS		Sensible heat flux, gapfilled using MDS method
HH	W m-2	
DD	W m-2	average from half-hourly data
WW-YY	W m-2	average from daily data
H_F_MDS_QC		Quality flag for H_F_MDS, H_CORR, H_CORR25, and H_CORR75.
HH	nondimensional	0 = measured; 1 = good quality gapfill; 2 = medium; 3 = poor
DD	nondimensional	fraction between 0-1, indicating percentage of measured and good quality gapfill data
WW-YY	nondimensional	fraction between 0-1, indicating percentage of measured and good quality gapfill data (average from daily data)
H_CORR		Sensible heat flux, corrected H_F_MDS by energy balance closure correction factor

HH	W m-2	
DD	W m-2	average from half-hourly data
WW-YY	W m-2	average from daily data
H_CORR_25		Sensible heat flux, corrected H_F_MDS by energy balance closure correction factor, 25th percentile
HH	W m-2	
DD	W m-2	average from half-hourly data
WW-YY		not available
H_CORR_75		Sensible heat flux, corrected H_F_MDS by energy balance closure correction factor, 75th percentile
HH	W m-2	
DD	W m-2	average from half-hourly data
WW-YY		not available
H_RANDUNC		Random uncertainty of H, from measured only data
HH	W m-2	uses only data point where H_F_MDS_QC is 0 and two hierarchical methods (see header and H_RANDUNC_METHOD)
DD-YY	W m-2	from random uncertainty of individual half-hours (rand(i)) = [SQRT(SUM(rand(i)^2)) / n], where n is the number of half-hours used
H_RANDUNC_METHOD		Method used to estimate the random uncertainty of H
HH	nondimensional	1 = RANDUNC Method 1 (direct SD method), 2 = RANDUNC Method 2 (median SD method)
DD-YY		not available
H_RANDUNC_N		Number of half-hour data points used to estimate the random uncertainty of H
HH	nondimensional	
DD-YY		not available
H_CORR_JOINTUNC		Joint uncertainty estimation for H
HH-DD	W m-2	[SQRT(H_RANDUNC^2 + ((H_CORR75 - H_CORR25) / 1.349)^2)]
WW-YY		not available

EBC_CF_N		Number of data points used to calculate energy closure balance correction factor. Driver data points within sliding window (ECB_CF Method 1) or number of ECB_CF data points (for ECB_CF Methods 2 and 3)
HH	nondimensional	for ECB_CF Method 1 (minimum 5, maximum 93)
DD	nondimensional	for ECB_CF Method 1 (minimum 5, maximum 15)
WW-YY	nondimensional	fraction between 0-1, indicating percentages of half-hours used with respect to theoretical maximum number of half hours
EBC_CF_METHOD		Method used to calculate the energy balance closure correction factor
HH-YY	nondimensional	1 = ECB_CF Method 1, 2 = ECB_CF Method 2, 3 = ECB_CF Method 3. See general description for details
NET ECOSYSTEM EXCHANGE		
NIGHT		Flag indicating nighttime interval based on SW_IN_POT
HH	nondimensional	0 = daytime, 1 = nighttime
DD-YY		not available
NIGHT_D		Number of half hours classified as nighttime in the period, i.e., when SW_IN_POT is 0
HH		not available
DD	nondimensional	number of half-hours
WW-MM	nondimensional	number of halfhours (average of the daily data)
YY		not available
DAY_D		Number of half hours classified as daytime in the period, i.e., when SW_IN_POT is greater than 0
HH		not available
DD	nondimensional	number of half-hours
WW-MM	nondimensional	number of halfhours (average of the daily data)
YY		not available

NIGHT_RANDUNC_N		Number of half hours classified as nighttime and used to calculate the aggregated random uncertainty
HH		not available
DD	nondimensional	number of half-hours
WW-YY	nondimensional	number of halfhours (average of the daily data)
DAY_RANDUNC_N		Number of half hours classified as daytime and used to calculate the aggregated random uncertainty
HH		not available
DD	nondimensional	number of half-hours
WW-YY	nondimensional	number of halfhours (average of the daily data)
NEE_CUT_REF		Net Ecosystem Exchange, using Constant Ustar Threshold (CUT) across years, reference selected on the basis of the model efficiency (MEF). The MEF analysis is repeated for each time aggregation
HH	µmolCO2 m-2 s-1	
DD	gC m-2 d-1	calculated from half-hourly data
WW-MM	gC m-2 d-1	average from daily data
YY	gC m-2 y-1	sum from daily data
NEE_VUT_REF		Net Ecosystem Exchange, using Variable Ustar Threshold (VUT) for each year, reference selected on the basis of the model efficiency (MEF). The MEF analysis is repeated for each time aggregation
HH	µmolCO2 m-2 s-1	
DD	gC m-2 d-1	calculated from half-hourly data
WW-MM	gC m-2 d-1	average from daily data
YY	gC m-2 y-1	sum from daily data
NEE_CUT_REF_QC		Quality flag for NEE_CUT_REF
HH	nondimensional	0 = measured; 1 = good quality gapfill; 2 = medium; 3 = poor
DD	nondimensional	fraction between 0-1, indicating percentage of measured and good quality gapfill data

WW-YY	nondimensional	fraction between 0-1, indicating percentage of measured and good quality gapfill data (average from daily data)
NEE_VUT_REF_QC		Quality flag for NEE_VUT_REF
HH	nondimensional	0 = measured; 1 = good quality gapfill; 2 = medium; 3 = poor
DD	nondimensional	fraction between 0-1, indicating percentage of measured and good quality gapfill data
WW-YY	nondimensional	fraction between 0-1, indicating percentage of measured and good quality gapfill data (average from daily data)
NEE_CUT_REF_RANDUNC		Random uncertainty for NEE_CUT_REF, from measured only data
HH	µmolCO2 m-2 s-1	uses only data points where NEE_CUT_REF_QC is 0 and two hierarchical methods - see header and NEE_CUT_REF_RANDUNC_METHOD
DD-MM	gC m-2 d-1	from random uncertainty of individual half-hours (rand(i)) = [SQRT(SUM(rand(i)^2)) / n], where n is the number of half-hours used
YY	gC m-2 y-1	from random uncertainty of individual half-hours (rand(i)) = [SQRT(SUM(rand(i)^2)) / n], where n is the number of half-hours used
NEE_VUT_REF_RANDUNC		Random uncertainty for NEE_VUT_REF, from measured only data
HH	µmolCO2 m-2 s-1	uses only data points where NEE_VUT_REF_QC is 0 and two hierarchical methods - see header and NEE_VUT_REF_RANDUNC_METHOD
DD-MM	gC m-2 d-1	from random uncertainty of individual half-hours (rand(i)) = [SQRT(SUM(rand(i)^2)) / n], where n is the number of half-hours used
YY	gC m-2 y-1	from random uncertainty of individual half-hours (rand(i)) = [SQRT(SUM(rand(i)^2)) / n], where n is the number of half-hours used
NEE_CUT_REF_RANDUNC_METHOD		Method used to estimate the random uncertainty of NEE_CUT_REF

HH	nondimensional	1 = RANDUNC Method 1 (direct SD method), 2 = RANDUNC Method 2 (median SD method)
DD-YY		not available
NEE_VUT_REF_RANDUNC_METHOD		Method used to estimate the random uncertainty of NEE_VUT_REF
HH	nondimensional	1 = RANDUNC Method 1 (direct SD method), 2 = RANDUNC Method 2 (median SD method)
DD-YY		not available
NEE_CUT_REF_RANDUNC_N		Number of data points used to estimate the random uncertainty of NEE_CUT_REF
HH	nondimensional	
DD-YY		not available
NEE_VUT_REF_RANDUNC_N		Number of data points used to estimate the random uncertainty of NEE_VUT_REF
HH	nondimensional	
DD-YY		not available
NEE_CUT_REF_JOINTUNC		Joint uncertainty estimation for NEE_CUT_REF, including random uncertainty and USTAR filtering uncertainty
HH	μmolCO2 m-2 s-1	[SQRT(NEE_CUT_REF_RANDUNC^2 + ((NEE_CUT_84 - NEE_CUT_16) / 2)^2)] for each half-hour
DD	gC m-2 d-1	[SQRT(NEE_CUT_REF_RANDUNC^2 + ((NEE_CUT_84 - NEE_CUT_16) / 2)^2)] for each day
WW	gC m-2 d-1	[SQRT(NEE_CUT_REF_RANDUNC^2 + ((NEE_CUT_84 - NEE_CUT_16) / 2)^2)] for each week
MM	gC m-2 d-1	[SQRT(NEE_CUT_REF_RANDUNC^2 + ((NEE_CUT_84 - NEE_CUT_16) / 2)^2)] for each month
YY	gC m-2 y-1	[SQRT(NEE_CUT_REF_RANDUNC^2 + ((NEE_CUT_84 - NEE_CUT_16) / 2)^2)] for each year
NEE_VUT_REF_JOINTUNC		Joint uncertainty estimation for NEE_VUT_REF, including random uncertainty and USTAR filtering uncertainty

HH	μmolCO2 m-2 s-1	[SQRT(NEE_VUT_REF_RANDUNC^2 + ((NEE_VUT_84 - NEE_VUT_16) / 2)^2)] for each half-hour
DD	gC m-2 d-1	[SQRT(NEE_VUT_REF_RANDUNC^2 + ((NEE_VUT_84 - NEE_VUT_16) / 2)^2)] for each day
WW	gC m-2 d-1	[SQRT(NEE_VUT_REF_RANDUNC^2 + ((NEE_VUT_84 - NEE_VUT_16) / 2)^2)] for each week
MM	gC m-2 d-1	[SQRT(NEE_VUT_REF_RANDUNC^2 + ((NEE_VUT_84 - NEE_VUT_16) / 2)^2)] for each month
YY	gC m-2 y-1	[SQRT(NEE_VUT_REF_RANDUNC^2 + ((NEE_VUT_84 - NEE_VUT_16) / 2)^2)] for each year
NEE_CUT_USTAR50		Net Ecosystem Exchange, using Constant Ustar Threshold (CUT) across years, from 50 percentile of USTAR threshold
HH	μmolCO2 m-2 s-1	
DD	gC m-2 d-1	calculated from half-hourly data
WW-MM	gC m-2 d-1	average from daily data
YY	gC m-2 y-1	sum from daily data
NEE_VUT_USTAR50		Net Ecosystem Exchange, using Variable Ustar Threshold (VUT) for each year, from 50 percentile of USTAR threshold
HH	μmolCO2 m-2 s-1	
DD	gC m-2 d-1	calculated from half-hourly data
WW-MM	gC m-2 d-1	average from daily data
YY	gC m-2 y-1	sum from daily data
NEE_CUT_USTAR50_QC		Quality flag for NEE_CUT_USTAR50
HH	nondimensional	0 = measured; 1 = good quality gapfill; 2 = medium; 3 = poor
DD	nondimensional	fraction between 0-1, indicating percentage of measured and good quality gapfill data
WW-YY	nondimensional	fraction between 0-1, indicating percentage of measured and good quality gapfill data (average from daily data)
NEE_VUT_USTAR50_QC		Quality flag for NEE_VUT_USTAR50

HH	nondimensional	0 = measured; 1 = good quality gapfill; 2 = medium; 3 = poor
DD	nondimensional	fraction between 0-1, indicating percentage of measured and good quality gapfill data
WW-YY	nondimensional	fraction between 0-1, indicating percentage of measured and good quality gapfill data (average from daily data)
NEE_CUT_USTAR50_RANDUNC		Random uncertainty for NEE_CUT_USTAR50, from measured only data
HH	µmolCO2 m-2 s-1	uses only data points where NEE_CUT_USTAR50_QC is 0 and two hierarchical methods - see header and NEE_CUT_USTAR50_RANDUNC_METHOD
DD-MM	gC m-2 d-1	from random uncertainty of individual half-hours (rand(i)) = [SQRT(SUM(rand(i)^2)) / n], where n is the number of half-hours used
YY	gC m-2 y-1	from random uncertainty of individual half-hours (rand(i)) = [SQRT(SUM(rand(i)^2)) / n], where n is the number of half-hours used
NEE_VUT_USTAR50_RANDUNC		Random uncertainty for NEE_VUT_USTAR50, from measured only data
HH	µmolCO2 m-2 s-1	uses only data points where NEE_VUT_USTAR50_QC is 0 and two hierarchical methods see header and NEE_VUT_USTAR50_RANDUNC_METHOD
DD-MM	gC m-2 d-1	from random uncertainty of individual half-hours (rand(i)) = [SQRT(SUM(rand(i)^2)) / n], where n is the number of half-hours used
YY	gC m-2 y-1	from random uncertainty of individual half-hours (rand(i)) = [SQRT(SUM(rand(i)^2)) / n], where n is the number of half-hours used
NEE_CUT_USTAR50_RANDUNC_METHOD		Method used to estimate the random uncertainty of NEE_CUT_USTAR50

HH	nondimensional	1 = RANDUNC Method 1 (direct SD method), 2 = RANDUNC Method 2 (median SD method)
DD-YY		not available
NEE_VUT_USTAR50_RANDUNC_METHOD		Method used to estimate the random uncertainty of NEE_VUT_USTAR50
HH	nondimensional	1 = RANDUNC Method 1 (direct SD method), 2 = RANDUNC Method 2 (median SD method)
DD-YY		not available
NEE_CUT_USTAR50_RANDUNC_N		Number of half-hour data points used to estimate the random uncertainty of NEE_CUT_USTAR50
HH	nondimensional	
DD-YY		not available
NEE_VUT_USTAR50_RANDUNC_N		Number of half-hour data points used to estimate the random uncertainty of NEE_VUT_USTAR50
HH	nondimensional	
DD-YY		not available
NEE_CUT_USTAR50_JOINTUNC		Joint uncertainty estimation for NEE_CUT_USTAR50, including random uncertainty and USTAR filtering uncertainty
HH	µmolCO2 m-2 s-1	[SQRT(NEE_CUT_USTAR50_RANDUNC^2 + ((NEE_CUT_84 - NEE_CUT_16) / 2)^2)] for each half-hour
DD	gC m-2 d-1	[SQRT(NEE_CUT_USTAR50_RANDUNC^2 + ((NEE_CUT_84 - NEE_CUT_16) / 2)^2)] for each day
WW	gC m-2 d-1	[SQRT(NEE_CUT_USTAR50_RANDUNC^2 + ((NEE_CUT_84 - NEE_CUT_16) / 2)^2)] for each week
MM	gC m-2 d-1	[SQRT(NEE_CUT_USTAR50_RANDUNC^2 + ((NEE_CUT_84 - NEE_CUT_16) / 2)^2)] for each month
YY	gC m-2 y-1	[SQRT(NEE_CUT_USTAR50_RANDUNC^2 + ((NEE_CUT_84 - NEE_CUT_16) / 2)^2)] for each year

NEE_VUT_USTAR50_JOINTUNC		Joint uncertainty estimation for NEE_VUT_USTAR50, including random uncertainty and USTAR filtering uncertainty
HH	μmolCO2 m-2 s-1	[SQRT(NEE_VUT_USTAR50_RANDUNC^2 + ((NEE_VUT_84 - NEE_VUT_16) / 2)^2)] for each half-hour
DD	gC m-2 d-1	[SQRT(NEE_VUT_USTAR50_RANDUNC^2 + ((NEE_VUT_84 - NEE_VUT_16) / 2)^2)] for each day
WW	gC m-2 d-1	[SQRT(NEE_VUT_USTAR50_RANDUNC^2 + ((NEE_VUT_84 - NEE_VUT_16) / 2)^2)] for each week
MM	gC m-2 d-1	[SQRT(NEE_VUT_USTAR50_RANDUNC^2 + ((NEE_VUT_84 - NEE_VUT_16) / 2)^2)] for each month
YY	gC m-2 y-1	[SQRT(NEE_VUT_USTAR50_RANDUNC^2 + ((NEE_VUT_84 - NEE_VUT_16) / 2)^2)] for each year
NEE_CUT_MEAN		Net Ecosystem Exchange, using Constant Ustar Threshold (CUT) across years, average from 40 NEE_CUT_XX versions
HH	μmolCO2 m-2 s-1	average from 40 half-hourly NEE_CUT_XX
DD	gC m-2 d-1	average from 40 daily NEE_CUT_XX
WW	gC m-2 d-1	average from 40 weekly NEE_CUT_XX
MM	gC m-2 d-1	average from 40 monthly NEE_CUT_XX
YY	gC m-2 y-1	average from 40 yearly NEE_CUT_XX
NEE_VUT_MEAN		Net Ecosystem Exchange, using Variable Ustar Threshold (VUT) for each year, average from 40 NEE_VUT_XX versions
HH	μmolCO2 m-2 s-1	average from 40 half-hourly NEE_CUT_XX
DD	gC m-2 d-1	average from 40 daily NEE_CUT_XX
WW	gC m-2 d-1	average from 40 weekly NEE_CUT_XX
MM	gC m-2 d-1	average from 40 monthly NEE_CUT_XX
YY	gC m-2 y-1	average from 40 yearly NEE_CUT_XX
NEE_CUT_MEAN_QC		Quality flag for NEE_CUT_MEAN, fraction between 0-1 indicating percentage of good quality data

HH	nondimensional	average of percentages of good data (NEE_CUT_XX_QC is 0 or 1) from 40 NEE_CUT_XX_QC
DD-YY	nondimensional	average of 40 NEE_CUT_XX_QC for the period
NEE_VUT_MEAN_QC		Quality flag for NEE_VUT_MEAN, fraction between 0-1 indicating percentage of good quality data
HH	nondimensional	average of percentages of good data (NEE_VUT_XX_QC is 0 or 1) from 40 NEE_VUT_XX_QC
DD-YY	nondimensional	average of 40 NEE_VUT_XX_QC for the period
NEE_CUT_SE		Standard Error for NEE_CUT, calculated as SD(NEE_CUT_XX) / SQRT(40)
HH	µmolCO2 m-2 s-1	SE from 40 half-hourly NEE_CUT_XX
DD	gC m-2 d-1	SE from 40 daily NEE_CUT_XX
WW	gC m-2 d-1	SE from 40 weekly NEE_CUT_XX
MM	gC m-2 d-1	SE from 40 monthly NEE_CUT_XX
YY	gC m-2 y-1	SE from 40 yearly NEE_CUT_XX
NEE_VUT_SE		Standard Error for NEE_VUT, calculated as SD(NEE_VUT_XX) / SQRT(40)
HH	µmolCO2 m-2 s-1	SE from 40 half-hourly NEE_CUT_XX
DD	gC m-2 d-1	SE from 40 daily NEE_CUT_XX
WW	gC m-2 d-1	SE from 40 weekly NEE_CUT_XX
MM	gC m-2 d-1	SE from 40 monthly NEE_CUT_XX
YY	gC m-2 y-1	SE from 40 yearly NEE_CUT_XX
NEE_CUT_XX		NEE CUT percentiles (approx. percentile indicated by XX, see doc.) calculated from the 40 estimates aggregated at the different time resolutions -- XX = 05, 16, 25, 50, 75, 84, 95
HH	µmolCO2 m-2 s-1	XXth percentile from 40 half-hourly NEE_CUT_XX
DD	gC m-2 d-1	XXth percentile from 40 daily NEE_CUT_XX
WW	gC m-2 d-1	XXth percentile from 40 weekly NEE_CUT_XX
MM	gC m-2 d-1	XXth percentile from 40 monthly NEE_CUT_XX

YY	gC m-2 y-1	XXth percentile from 40 yearly NEE_CUT_XX
NEE_VUT_XX		NEE VUT percentiles (approx. percentile indicated by XX, see doc.) calculated from the 40 estimates aggregated at the different time resolutions -- XX = 05, 16, 25, 50, 75, 84, 95
HH	µmolCO2 m-2 s-1	XXth percentile from 40 half-hourly NEE_VUT_XX
DD	gC m-2 d-1	XXth percentile from 40 daily NEE_VUT_XX
WW	gC m-2 d-1	XXth percentile from 40 weekly NEE_VUT_XX
MM	gC m-2 d-1	XXth percentile from 40 monthly NEE_VUT_XX
YY	gC m-2 y-1	XXth percentile from 40 yearly NEE_VUT_XX
NEE_CUT_XX_QC		Quality flag for NEE_CUT_XX -- XX = 05, 16, 25, 50, 75, 84, 95
HH	nondimensional	0 = measured; 1 = good quality gapfill; 2 = medium; 3 = poor
DD	nondimensional	fraction between 0-1, indicating percentage of measured and good quality gapfill data
WW-YY	nondimensional	fraction between 0-1, indicating percentage of measured and good quality gapfill data (average from daily data)
NEE_VUT_XX_QC		Quality flag for NEE_VUT_XX -- XX = 05, 16, 25, 50, 75, 84, 95
HH	nondimensional	0 = measured; 1 = good quality gapfill; 2 = medium; 3 = poor
DD	nondimensional	fraction between 0-1, indicating percentage of measured and good quality gapfill data
WW-YY	nondimensional	fraction between 0-1, indicating percentage of measured and good quality gapfill data (average from daily data)
NEE_CUT_REF_NIGHT		Average nighttime NEE, from NEE_CUT_REF
HH		not available
DD	µmolCO2 m-2 s-1	average from half-hourly data (where NIGHT is 1)
WW-YY	µmolCO2 m-2 s-1	average from daily data

NEE_VUT_REF_NIGHT		Average nighttime NEE, from NEE_VUT_REF
HH		not available
DD	µmolCO2 m-2 s-1	average from half-hourly data (where NIGHT is 1)
WW-YY	µmolCO2 m-2 s-1	average from daily data
NEE_CUT_REF_NIGHT_SD		Standard Deviation of the nighttime NEE, from the NEE_CUT_REF
HH		not available
DD	µmolCO2 m-2 s-1	from half-hourly data (where NIGHT is 1)
WW-YY	µmolCO2 m-2 s-1	from daily data
NEE_VUT_REF_NIGHT_SD		Standard Deviation of the nighttime NEE, from the NEE_VUT_REF
HH		not available
DD	µmolCO2 m-2 s-1	from half-hourly data (where NIGHT is 1)
WW-YY	µmolCO2 m-2 s-1	from daily data
NEE_CUT_REF_NIGHT_QC		Quality flag for NEE_CUT_REF_NIGHT
HH		not available
DD	nondimensional	fraction between 0-1, indicating percentage of measured and good quality gapfill data
WW-YY	nondimensional	fraction between 0-1, indicating percentage of measured and good quality gapfill data (average from daily data)
NEE_VUT_REF_NIGHT_QC		Quality flag for NEE_VUT_REF_NIGHT
HH		not available
DD	nondimensional	fraction between 0-1, indicating percentage of measured and good quality gapfill data
WW-YY	nondimensional	fraction between 0-1, indicating percentage of measured and good quality gapfill data (average from daily data)
NEE_CUT_REF_NIGHT_RANDUNC		Random uncertainty of NEE_CUT_REF_NIGHT, from the random uncertainty of the single nighttime half-hours
HH		not available
DD-YY	µmolCO2 m-2 s-1	from random uncertainty of individual half-hours where NIGHT is 1 (rand(i)) = [SQRT(SUM(rand(i)^2)) / n], where n is the

		number of half-hours used to calculate the nighttime aggregation in the day.
NEE_VUT_REF_NIGHT_RANDUNC		Random uncertainty of NEE_VUT_REF_NIGHT, from the random uncertainty of the single nighttime half-hours
HH		not available
DD-YY	µmolCO2 m-2 s-1	from random uncertainty of individual half-hours where NIGHT is 1 (rand(i)) = [SQRT(SUM(rand(i)^2)) / n], where n is the number of half-hours used to calculate the nighttime aggregation in the day.
NEE_CUT_REF_NIGHT_JOINTUNC		Joint uncertainty estimation for NEE_CUT_REF_NIGHT, including random uncertainty and USTAR filtering uncertainty
HH		not available
DD	µmolCO2 m-2 s-1	[SQRT(NEE_CUT_REF_NIGHT_RANDUNC ^2 + ((NEE_CUT_84_NIGHT - NEE_CUT_16_NIGHT) / 2)^2)] for each day
WW	µmolCO2 m-2 s-1	[SQRT(NEE_CUT_REF_NIGHT_RANDUNC ^2 + ((NEE_CUT_84_NIGHT - NEE_CUT_16_NIGHT) / 2)^2)] for each week
MM	µmolCO2 m-2 s-1	[SQRT(NEE_CUT_REF_NIGHT_RANDUNC ^2 + ((NEE_CUT_84_NIGHT - NEE_CUT_16_NIGHT) / 2)^2)] for each month
YY	µmolCO2 m-2 s-1	[SQRT(NEE_CUT_REF_NIGHT_RANDUNC ^2 + ((NEE_CUT_84_NIGHT - NEE_CUT_16_NIGHT) / 2)^2)] for each year
NEE_VUT_REF_NIGHT_JOINTUNC		Joint uncertainty estimation for NEE_VUT_REF_NIGHT, including random uncertainty and USTAR filtering uncertainty
HH		not available
DD	µmolCO2 m-2 s-1	[SQRT(NEE_VUT_REF_NIGHT_RANDUNC ^2 + ((NEE_VUT_84_NIGHT - NEE_VUT_16_NIGHT) / 2)^2)] for each day
WW	µmolCO2 m-2 s-1	[SQRT(NEE_VUT_REF_NIGHT_RANDUNC ^2 + ((NEE_VUT_84_NIGHT - NEE_VUT_16_NIGHT) / 2)^2)] for each week

MM	μmolCO2 m-2 s-1	[SQRT(NEE_VUT_REF_NIGHT_RANDUNC ^2 + ((NEE_VUT_84_NIGHT - NEE_VUT_16_NIGHT) / 2)^2)] for each month
YY	μmolCO2 m-2 s-1	[SQRT(NEE_VUT_REF_NIGHT_RANDUNC ^2 + ((NEE_VUT_84_NIGHT - NEE_VUT_16_NIGHT) / 2)^2)] for each year
NEE_CUT_REF_DAY		Average daytime NEE, from NEE_CUT_REF
HH		not available
DD	μmolCO2 m-2 s-1	average from half-hourly data (where NIGHT is 0)
WW-YY	μmolCO2 m-2 s-1	average from daily data
NEE_VUT_REF_DAY		Average daytime NEE, from NEE_VUT_REF
HH		not available
DD	μmolCO2 m-2 s-1	average from half-hourly data (where NIGHT is 0)
WW-YY	μmolCO2 m-2 s-1	average from daily data
NEE_CUT_REF_DAY_SD		Standard Deviation of the daytime NEE, from the NEE_CUT_REF
HH		not available
DD	μmolCO2 m-2 s-1	from half-hourly data (where NIGHT is 0)
WW-YY	μmolCO2 m-2 s-1	from daily data
NEE_VUT_REF_DAY_SD		Standard Deviation of the daytime NEE, from the NEE_VUT_REF
HH		not available
DD	μmolCO2 m-2 s-1	from half-hourly data (where NIGHT is 0)
WW-YY	μmolCO2 m-2 s-1	from daily data
NEE_CUT_REF_DAY_QC		Quality flag for NEE_CUT_REF_DAY
HH		not available
DD	nondimensional	fraction between 0-1, indicating percentage of measured and good quality gapfill data
WW-YY	nondimensional	fraction between 0-1, indicating percentage of measured and good quality gapfill data (average from daily data)
NEE_VUT_REF_DAY_QC		Quality flag for NEE_VUT_REF_DAY
HH		not available

DD	nondimensional	fraction between 0-1, indicating percentage of measured and good quality gapfill data
WW-YY	nondimensional	fraction between 0-1, indicating percentage of measured and good quality gapfill data (average from daily data)
NEE_CUT_REF_DAY_RANDUNC		Random uncertainty of NEE_CUT_REF_DAY, from the random uncertainty of the single daytime half-hours
HH		not available
DD-YY	μmolCO2 m-2 s-1	from random uncertainty of individual half-hours where NIGHT is 0 (rand(i)) = [SQRT(SUM(rand(i)^2)) / n], where n is the number of half-hours used to calculate the daytime aggregation in the day.
NEE_VUT_REF_DAY_RANDUNC		Random uncertainty of NEE_VUT_REF_DAY, from the random uncertainty of the single daytime half-hours
HH		not available
DD-YY	μmolCO2 m-2 s-1	from random uncertainty of individual half-hours where NIGHT is 0 (rand(i)) = [SQRT(SUM(rand(i)^2)) / n], where n is the number of half-hours used to calculate the daytime aggregation in the day.
NEE_CUT_REF_DAY_JOINTUNC		Joint uncertainty estimation for NEE_CUT_REF_DAY, including random uncertainty and USTAR filtering uncertainty
HH		not available
DD	μmolCO2 m-2 s-1	[SQRT(NEE_CUT_REF_DAY_RANDUNC^2 + ((NEE_CUT_84_DAY - NEE_CUT_16_DAY) / 2)^2)] for each day
WW	μmolCO2 m-2 s-1	[SQRT(NEE_CUT_REF_DAY_RANDUNC^2 + ((NEE_CUT_84_DAY - NEE_CUT_16_DAY) / 2)^2)] for each week
MM	μmolCO2 m-2 s-1	[SQRT(NEE_CUT_REF_DAY_RANDUNC^2 + ((NEE_CUT_84_DAY - NEE_CUT_16_DAY) / 2)^2)] for each month
YY	μmolCO2 m-2 s-1	[SQRT(NEE_CUT_REF_DAY_RANDUNC^2 + ((NEE_CUT_84_DAY - NEE_CUT_16_DAY) / 2)^2)] for each year

NEE_VUT_REF_DAY_JOINTUNC		Joint uncertainty estimation for NEE_VUT_REF_DAY, including random uncertainty and USTAR filtering uncertainty
HH		not available
DD	µmolCO2 m-2 s-1	[SQRT(NEE_VUT_REF_DAY_RANDUNC^2 + ((NEE_VUT_84_DAY - NEE_VUT_16_DAY) / 2)^2)] for each day
WW	µmolCO2 m-2 s-1	[SQRT(NEE_VUT_REF_DAY_RANDUNC^2 + ((NEE_VUT_84_DAY - NEE_VUT_16_DAY) / 2)^2)] for each week
MM	µmolCO2 m-2 s-1	[SQRT(NEE_VUT_REF_DAY_RANDUNC^2 + ((NEE_VUT_84_DAY - NEE_VUT_16_DAY) / 2)^2)] for each month
YY	µmolCO2 m-2 s-1	[SQRT(NEE_VUT_REF_DAY_RANDUNC^2 + ((NEE_VUT_84_DAY - NEE_VUT_16_DAY) / 2)^2)] for each year
NEE_CUT_USTAR50_NIGHT		Average nighttime NEE, from NEE_CUT_USTAR50
HH		not available
DD	µmolCO2 m-2 s-1	average from half-hourly data (where NIGHT is 1)
WW-YY	µmolCO2 m-2 s-1	average from daily data
NEE_VUT_USTAR50_NIGHT		Average nighttime NEE, from NEE_VUT_USTAR50
HH		not available
DD	µmolCO2 m-2 s-1	average from half-hourly data (where NIGHT is 1)
WW-YY	µmolCO2 m-2 s-1	average from daily data
NEE_CUT_USTAR50_NIGHT_SD		Standard Deviation of the nighttime NEE, from the NEE_CUT_USTAR50
HH		not available
DD	µmolCO2 m-2 s-1	from half-hourly data (where NIGHT is 1)
WW-YY	µmolCO2 m-2 s-1	from daily data
NEE_VUT_USTAR50_NIGHT_SD		Standard Deviation of the nighttime NEE, from the NEE_VUT_USTAR50
HH		not available
DD	µmolCO2 m-2 s-1	from half-hourly data (where NIGHT is 1)
WW-YY	µmolCO2 m-2 s-1	from daily data

NEE_CUT_USTAR50_NIGHT_QC		Quality flag for NEE_CUT_USTAR50_NIGHT
HH		not available
DD	nondimensional	fraction between 0-1, indicating percentage of measured and good quality gapfill data
WW-YY	nondimensional	fraction between 0-1, indicating percentage of measured and good quality gapfill data (average from daily data)
NEE_VUT_USTAR50_NIGHT_QC		Quality flag for NEE_VUT_USTAR50_NIGHT
HH		not available
DD	nondimensional	fraction between 0-1, indicating percentage of measured and good quality gapfill data
WW-YY	nondimensional	fraction between 0-1, indicating percentage of measured and good quality gapfill data (average from daily data)
NEE_CUT_USTAR50_NIGHT_RANDUNC		Random uncertainty of NEE_CUT_USTAR50_NIGHT, from the random uncertainty of the single nighttime half-hours
HH		not available
DD-YY	μmolCO2 m-2 s-1	from random uncertainty of individual half-hours where NIGHT is 1 (rand(i)) = [SQRT(SUM(rand(i)^2)) / n], where n is the number of half-hours used to calculate the nighttime aggregation in the day.
NEE_VUT_USTAR50_NIGHT_RANDUNC		Random uncertainty of NEE_VUT_USTAR50_NIGHT, from the random uncertainty of the single nighttime half-hours
HH		not available
DD-YY	μmolCO2 m-2 s-1	from random uncertainty of individual half-hours where NIGHT is 1 (rand(i)) = [SQRT(SUM(rand(i)^2)) / n], where n is the number of half-hours used to calculate the nighttime aggregation in the day.
NEE_CUT_USTAR50_NIGHT_JOINTUNC		Joint uncertainty estimation for NEE_CUT_USTAR50_NIGHT, including random uncertainty and USTAR filtering uncertainty
HH		not available

DD	µmolCO2 m-2 s-1	[SQRT(NEE_CUT_USTAR50_NIGHT_RANDUNC^2 + ((NEE_CUT_84_NIGHT - NEE_CUT_16_NIGHT) / 2)^2)] for each day
WW	µmolCO2 m-2 s-1	[SQRT(NEE_CUT_USTAR50_NIGHT_RANDUNC^2 + ((NEE_CUT_84_NIGHT - NEE_CUT_16_NIGHT) / 2)^2)] for each week
MM	µmolCO2 m-2 s-1	[SQRT(NEE_CUT_USTAR50_NIGHT_RANDUNC^2 + ((NEE_CUT_84_NIGHT - NEE_CUT_16_NIGHT) / 2)^2)] for each month
YY	µmolCO2 m-2 s-1	[SQRT(NEE_CUT_USTAR50_NIGHT_RANDUNC^2 + ((NEE_CUT_84_NIGHT - NEE_CUT_16_NIGHT) / 2)^2)] for each year
NEE_VUT_USTAR50_NIGHT_JOINTUNC		Joint uncertainty estimation for NEE_VUT_USTAR50_NIGHT, including random uncertainty and USTAR filtering uncertainty
HH		not available
DD	µmolCO2 m-2 s-1	[SQRT(NEE_VUT_USTAR50_NIGHT_RANDUNC^2 + ((NEE_VUT_84_NIGHT - NEE_VUT_16_NIGHT) / 2)^2)] for each day
WW	µmolCO2 m-2 s-1	[SQRT(NEE_VUT_USTAR50_NIGHT_RANDUNC^2 + ((NEE_VUT_84_NIGHT - NEE_VUT_16_NIGHT) / 2)^2)] for each week
MM	µmolCO2 m-2 s-1	[SQRT(NEE_VUT_USTAR50_NIGHT_RANDUNC^2 + ((NEE_VUT_84_NIGHT - NEE_VUT_16_NIGHT) / 2)^2)] for each month
YY	µmolCO2 m-2 s-1	[SQRT(NEE_VUT_USTAR50_NIGHT_RANDUNC^2 + ((NEE_VUT_84_NIGHT - NEE_VUT_16_NIGHT) / 2)^2)] for each year
NEE_CUT_USTAR50_DAY		Average daytime NEE, from NEE_CUT_USTAR50
HH		not available
DD	µmolCO2 m-2 s-1	average from half-hourly data (where NIGHT is 0)
WW-YY	µmolCO2 m-2 s-1	average from daily data
NEE_VUT_USTAR50_DAY		Average daytime NEE, from NEE_VUT_USTAR50

HH		not available
DD	µmolCO2 m-2 s-1	average from half-hourly data (where NIGHT is 0)
WW-YY	µmolCO2 m-2 s-1	average from daily data
NEE_CUT_USTAR50_DAY_SD		Standard Deviation of the daytime NEE, from the NEE_CUT_USTAR50
HH		not available
DD	µmolCO2 m-2 s-1	from half-hourly data (where NIGHT is 0)
WW-YY	µmolCO2 m-2 s-1	from daily data
NEE_VUT_USTAR50_DAY_SD		Standard Deviation of the daytime NEE, from the NEE_VUT_USTAR50
HH		not available
DD	µmolCO2 m-2 s-1	from half-hourly data (where NIGHT is 0)
WW-YY	µmolCO2 m-2 s-1	from daily data
NEE_CUT_USTAR50_DAY_QC		Quality flag for NEE_CUT_USTAR50_DAY
HH		not available
DD	nondimensional	fraction between 0-1, indicating percentage of measured and good quality gapfill data
WW-YY	nondimensional	fraction between 0-1, indicating percentage of measured and good quality gapfill data (average from daily data)
NEE_VUT_USTAR50_DAY_QC		Quality flag for NEE_VUT_USTAR50_DAY
HH		not available
DD	nondimensional	fraction between 0-1, indicating percentage of measured and good quality gapfill data
WW-YY	nondimensional	fraction between 0-1, indicating percentage of measured and good quality gapfill data (average from daily data)
NEE_CUT_USTAR50_DAY_RANDUNC		Random uncertainty of NEE_CUT_USTAR50_DAY, from the random uncertainty of the single daytime half-hours
HH		not available
DD-YY	µmolCO2 m-2 s-1	from random uncertainty of individual half-hours where NIGHT is 0 (rand(i)) = [SQRT(SUM(rand(i)^2)) / n], where n is the number of half-hours used to calculate the daytime aggregation in the day.

NEE_VUT_USTAR50_DAY_RANDUNC		Random uncertainty of NEE_VUT_USTAR50_DAY, from the random uncertainty of the single daytime half-hours
HH		not available
DD-YY	µmolCO2 m-2 s-1	from random uncertainty of individual half-hours where NIGHT is 0 (rand(i)) = [SQRT(SUM(rand(i)^2)) / n], where n is the number of half-hours used to calculate the daytime aggregation in the day.
NEE_CUT_USTAR50_DAY_JOINTUNC		Joint uncertainty estimation for NEE_CUT_USTAR50_DAY, including random uncertainty and USTAR filtering uncertainty
HH		not available
DD	µmolCO2 m-2 s-1	[SQRT(NEE_CUT_USTAR50_DAY_RANDUNC^2 + ((NEE_CUT_84_DAY - NEE_CUT_16_DAY) / 2)^2)] for each day
WW	µmolCO2 m-2 s-1	[SQRT(NEE_CUT_USTAR50_DAY_RANDUNC^2 + ((NEE_CUT_84_DAY - NEE_CUT_16_DAY) / 2)^2)] for each week
MM	µmolCO2 m-2 s-1	[SQRT(NEE_CUT_USTAR50_DAY_RANDUNC^2 + ((NEE_CUT_84_DAY - NEE_CUT_16_DAY) / 2)^2)] for each month
YY	µmolCO2 m-2 s-1	[SQRT(NEE_CUT_USTAR50_DAY_RANDUNC^2 + ((NEE_CUT_84_DAY - NEE_CUT_16_DAY) / 2)^2)] for each year
NEE_VUT_USTAR50_DAY_JOINTUNC		Joint uncertainty estimation for NEE_VUT_USTAR50_DAY, including random uncertainty and USTAR filtering uncertainty
HH		not available
DD	µmolCO2 m-2 s-1	SQRT(NEE_VUT_USTAR50_DAY_RANDUNC^2 + ((NEE_VUT_84_DAY - NEE_VUT_16_DAY) / 2)^2) for each day
WW	µmolCO2 m-2 s-1	SQRT(NEE_VUT_USTAR50_DAY_RANDUNC^2 + ((NEE_VUT_84_DAY - NEE_VUT_16_DAY) / 2)^2) for each week
MM	µmolCO2 m-2 s-1	SQRT(NEE_VUT_USTAR50_DAY_RANDUNC^2 + ((NEE_VUT_84_DAY - NEE_VUT_16_DAY) / 2)^2) for each month

YY	μmolCO2 m-2 s-1	SQRT(NEE_VUT_USTAR50_DAY_RANDUNC^2 + ((NEE_VUT_84_DAY - NEE_VUT_16_DAY) / 2)^2) for each year
NEE_CUT_XX_NIGHT		NEE CUT nighttime percentiles (approx. percentile indicated by XX, see doc.) calculated from the 40 estimates aggregated at the different time resolutions -- XX = 05, 16, 25, 50, 75, 84, 95
HH		not available
DD	μmolCO2 m-2 s-1	XXth nighttime percentile from 40 daily NEE_CUT_XX_NIGHT
WW	μmolCO2 m-2 s-1	XXth nighttime percentile from 40 weekly NEE_CUT_XX_NIGHT
MM	μmolCO2 m-2 s-1	XXth nighttime percentile from 40 monthly NEE_CUT_XX_NIGHT
YY	μmolCO2 m-2 s-1	XXth nighttime percentile from 40 yearly NEE_CUT_XX_NIGHT
NEE_VUT_XX_NIGHT		NEE VUT nighttime percentiles (approx. percentile indicated by XX, see doc.) calculated from the 40 estimates aggregated at the different time resolutions -- XX = 05, 16, 25, 50, 75, 84, 95
HH		not available
DD	μmolCO2 m-2 s-1	XXth nighttime percentile from 40 daily NEE_VUT_XX_NIGHT
WW	μmolCO2 m-2 s-1	XXth nighttime percentile from 40 weekly NEE_VUT_XX_NIGHT
MM	μmolCO2 m-2 s-1	XXth nighttime percentile from 40 monthly NEE_VUT_XX_NIGHT
YY	μmolCO2 m-2 s-1	XXth nighttime percentile from 40 yearly NEE_VUT_XX_NIGHT
NEE_CUT_XX_NIGHT_QC		Quality flag for NEE_CUT_XX_NIGHT -- XX = 05, 16, 25, 50, 75, 84, 95
HH		not available
DD	nondimensional	fraction between 0-1, indicating percentage of measured and good quality gapfill data
WW-YY	nondimensional	fraction between 0-1, indicating percentage of measured and good quality gapfill data (average from daily data)

NEE_VUT_XX_NIGHT_QC		Quality flag for NEE_VUT_XX_NIGHT -- XX = 05, 16, 25, 50, 75, 84, 95
HH		not available
DD	nondimensional	fraction between 0-1, indicating percentage of measured and good quality gapfill data
WW-YY	nondimensional	fraction between 0-1, indicating percentage of measured and good quality gapfill data (average from daily data)
NEE_CUT_XX_DAY		NEE CUT daytime percentiles (approx. percentile indicated by XX, see doc.) calculated from the 40 estimates aggregated at the different time resolutions -- XX = 05, 16, 25, 50, 75, 84, 95
HH		not available
DD	µmolCO2 m-2 s-1	XXth daytime percentile from 40 daily NEE_CUT_XX_DAY
WW	µmolCO2 m-2 s-1	XXth daytime percentile from 40 weekly NEE_CUT_XX_DAY
MM	µmolCO2 m-2 s-1	XXth daytime percentile from 40 monthly NEE_CUT_XX_DAY
YY	µmolCO2 m-2 s-1	XXth daytime percentile from 40 yearly NEE_CUT_XX_DAY
NEE_VUT_XX_DAY		NEE VUT daytime percentiles (approx. percentile indicated by XX, see doc.) calculated from the 40 estimates aggregated at the different time resolutions -- XX = 05, 16, 25, 50, 75, 84, 95
HH		not available
DD	µmolCO2 m-2 s-1	XXth daytime percentile from 40 daily NEE_VUT_XX_DAY
WW	µmolCO2 m-2 s-1	XXth daytime percentile from 40 weekly NEE_VUT_XX_DAY
MM	µmolCO2 m-2 s-1	XXth daytime percentile from 40 monthly NEE_VUT_XX_DAY
YY	µmolCO2 m-2 s-1	XXth daytime percentile from 40 yearly NEE_VUT_XX_DAY
NEE_CUT_XX_DAY_QC		Quality flag for NEE_CUT_XX_DAY -- XX = 05, 16, 25, 50, 75, 84, 95
HH		not available

DD	nondimensional	fraction between 0-1, indicating percentage of measured and good quality gapfill data
WW-YY	nondimensional	fraction between 0-1, indicating percentage of measured and good quality gapfill data (average from daily data)
NEE_VUT_XX_DAY_QC		Quality flag for NEE_VUT_XX_DAY -- XX = 05, 16, 25, 50, 75, 84, 95
HH		not available
DD	nondimensional	fraction between 0-1, indicating percentage of measured and good quality gapfill data
WW-YY	nondimensional	fraction between 0-1, indicating percentage of measured and good quality gapfill data (average from daily data)
PARTITIONING - NIGHTTIME		
RECO_NT_VUT_REF		Ecosystem Respiration, from Nighttime partitioning method, reference selected from RECO versions using model efficiency (MEF). The MEF analysis is repeated for each time aggregation
HH	µmolCO2 m-2 s-1	
DD	gC m-2 d-1	calculated from half-hourly data
WW-MM	gC m-2 d-1	average from daily data
YY	gC m-2 y-1	sum from daily data
RECO_NT_VUT_USTAR50		Ecosystem Respiration, from Nighttime partitioning method, based on NEE_VUT_USTAR50
HH	µmolCO2 m-2 s-1	
DD	gC m-2 d-1	calculated from half-hourly data
WW-MM	gC m-2 d-1	average from daily data
YY	gC m-2 y-1	sum from daily data
RECO_NT_VUT_MEAN		Ecosystem Respiration, from Nighttime partitioning method, average from RECO versions, each from corresponding NEE_VUT_XX version
HH	µmolCO2 m-2 s-1	average from 40 half-hourly RECO_NT_VUT_XX
DD	gC m-2 d-1	average from 40 daily RECO_NT_VUT_XX

WW	gC m-2 d-1	average from 40 weekly RECO_NT_VUT_XX
MM	gC m-2 d-1	average from 40 monthly RECO_NT_VUT_XX
YY	gC m-2 y-1	average from 40 yearly RECO_NT_VUT_XX
RECO_NT_VUT_SE		Standard Error for Ecosystem Respiration, calculated as (SD(RECO_NT_VUT_XX) / SQRT(40))
HH	µmolCO2 m-2 s-1	SE from 40 half-hourly RECO_NT_CUT_XX
DD	gC m-2 d-1	SE from 40 daily RECO_NT_VUT_XX
WW	gC m-2 d-1	SE from 40 weekly RECO_NT_VUT_XX
MM	gC m-2 d-1	SE from 40 monthly RECO_NT_VUT_XX
YY	gC m-2 y-1	SE from 40 yearly RECO_NT_VUT_XX
RECO_NT_VUT_XX		Ecosystem Respiration, from Nighttime partitioning method (with XX = 05, 16, 25, 50, 75, 84, 95)
HH	µmolCO2 m-2 s-1	
DD	gC m-2 d-1	calculated from half-hourly data
WW-MM	gC m-2 d-1	average from daily data
YY	gC m-2 y-1	sum from daily data
RECO_NT_CUT_REF		Ecosystem Respiration, from Nighttime partitioning method, reference selected from RECO versions using model efficiency (MEF). The MEF analysis is repeated for each time aggregation
HH	µmolCO2 m-2 s-1	
DD	gC m-2 d-1	calculated from half-hourly data
WW-MM	gC m-2 d-1	average from daily data
YY	gC m-2 y-1	sum from daily data
RECO_NT_CUT_USTAR50		Ecosystem Respiration, from Nighttime partitioning method, based on NEE_CUT_USTAR50
HH	µmolCO2 m-2 s-1	
DD	gC m-2 d-1	calculated from half-hourly data
WW-MM	gC m-2 d-1	average from daily data
YY	gC m-2 y-1	sum from daily data

RECO_NT_CUT_MEAN		Ecosystem Respiration, from Nighttime partitioning method, average from RECO versions, each from corresponding NEE_CUT_XX version
HH	µmolCO2 m-2 s-1	average from 40 half-hourly RECO_NT_CUT_XX
DD	gC m-2 d-1	average from 40 daily RECO_NT_CUT_XX
WW	gC m-2 d-1	average from 40 weekly RECO_NT_CUT_XX
MM	gC m-2 d-1	average from 40 monthly RECO_NT_CUT_XX
YY	gC m-2 y-1	average from 40 yearly RECO_NT_CUT_XX
RECO_NT_CUT_SE		Standard Error for Ecosystem Respiration, calculated as (SD(RECO_NT_CUT_XX) / SQRT(40))
HH	µmolCO2 m-2 s-1	SE from 40 half-hourly RECO_NT_CUT_XX
DD	gC m-2 d-1	SE from 40 daily RECO_NT_CUT_XX
WW	gC m-2 d-1	SE from 40 weekly RECO_NT_CUT_XX
MM	gC m-2 d-1	SE from 40 monthly RECO_NT_CUT_XX
YY	gC m-2 y-1	SE from 40 yearly RECO_NT_CUT_XX
RECO_NT_CUT_XX		Ecosystem Respiration, from Nighttime partitioning method (with XX = 05, 16, 25, 50, 75, 84, 95)
HH	µmolCO2 m-2 s-1	
DD	gC m-2 d-1	calculated from half-hourly data
WW-MM	gC m-2 d-1	average from daily data
YY	gC m-2 y-1	sum from daily data
GPP_NT_VUT_REF		Gross Primary Production, from Nighttime partitioning method, reference selected from GPP versions using model efficiency (MEF). The MEF analysis is repeated for each time aggregation
HH	µmolCO2 m-2 s-1	
DD	gC m-2 d-1	calculated from half-hourly data
WW-MM	gC m-2 d-1	average from daily data
YY	gC m-2 y-1	sum from daily data

GPP_NT_VUT_USTAR50		Gross Primary Production, from Nighttime partitioning method, based on NEE_VUT_USTAR50
HH	µmolCO2 m-2 s-1	
DD	gC m-2 d-1	calculated from half-hourly data
WW-MM	gC m-2 d-1	average from daily data
YY	gC m-2 y-1	sum from daily data
GPP_NT_VUT_MEAN		Gross Primary Production, from Nighttime partitioning method, average from GPP versions, each from corresponding NEE_VUT_XX version
HH	µmolCO2 m-2 s-1	average from 40 half-hourly GPP_NT_VUT_XX
DD	gC m-2 d-1	average from 40 daily GPP_NT_VUT_XX
WW	gC m-2 d-1	average from 40 weekly GPP_NT_VUT_XX
MM	gC m-2 d-1	average from 40 monthly GPP_NT_VUT_XX
YY	gC m-2 y-1	average from 40 yearly GPP_NT_VUT_XX
GPP_NT_VUT_SE		Standard Error for Gross Primary Production, calculated as (SD(GPP_NT_VUT_XX) / SQRT(40))
HH	µmolCO2 m-2 s-1	SE from 40 half-hourly GPP_NT_VUT_XX
DD	gC m-2 d-1	SE from 40 daily GPP_NT_VUT_XX
WW	gC m-2 d-1	SE from 40 weekly GPP_NT_VUT_XX
MM	gC m-2 d-1	SE from 40 monthly GPP_NT_VUT_XX
YY	gC m-2 y-1	SE from 40 yearly GPP_NT_VUT_XX
GPP_NT_VUT_XX		Gross Primary Production, from Nighttime partitioning method (with XX = 05, 16, 25, 50, 75, 84, 95)
HH	µmolCO2 m-2 s-1	
DD	gC m-2 d-1	calculated from half-hourly data
WW-MM	gC m-2 d-1	average from daily data
YY	gC m-2 y-1	sum from daily data
GPP_NT_CUT_REF		Gross Primary Production, from Nighttime partitioning method, reference selected from GPP versions using model efficiency (MEF). The MEF analysis is repeated for each time aggregation
HH	µmolCO2 m-2 s-1	

DD	gC m-2 d-1	calculated from half-hourly data
WW-MM	gC m-2 d-1	average from daily data
YY	gC m-2 y-1	sum from daily data
GPP_NT_CUT_USTAR50		Gross Primary Production, from Nighttime partitioning method, based on NEE_CUT_USTAR50
HH	µmolCO2 m-2 s-1	
DD	gC m-2 d-1	calculated from half-hourly data
WW-MM	gC m-2 d-1	average from daily data
YY	gC m-2 y-1	sum from daily data
GPP_NT_CUT_MEAN		Gross Primary Production, from Nighttime partitioning method, average from GPP versions, each from corresponding NEE_CUT_XX version
HH	µmolCO2 m-2 s-1	average from 40 half-hourly GPP_NT_CUT_XX
DD	gC m-2 d-1	average from 40 daily GPP_NT_CUT_XX
WW	gC m-2 d-1	average from 40 weekly GPP_NT_CUT_XX
MM	gC m-2 d-1	average from 40 monthly GPP_NT_CUT_XX
YY	gC m-2 y-1	average from 40 yearly GPP_NT_CUT_XX
GPP_NT_CUT_SE		Standard Error for Gross Primary Production, calculated as (SD(GPP_NT_CUT_XX) / SQRT(40))
HH	µmolCO2 m-2 s-1	SE from 40 half-hourly GPP_NT_CUT_XX
DD	gC m-2 d-1	SE from 40 daily GPP_NT_CUT_XX
WW	gC m-2 d-1	SE from 40 weekly GPP_NT_CUT_XX
MM	gC m-2 d-1	SE from 40 monthly GPP_NT_CUT_XX
YY	gC m-2 y-1	SE from 40 yearly GPP_NT_CUT_XX
GPP_NT_CUT_XX		Gross Primary Production, from Nighttime partitioning method (with XX = 05, 16, 25, 50, 75, 84, 95)
HH	µmolCO2 m-2 s-1	
DD	gC m-2 d-1	calculated from half-hourly data
WW-MM	gC m-2 d-1	average from daily data
YY	gC m-2 y-1	sum from daily data
PARTITIONING - DAYTIME		

RECO_DT_VUT_REF		Ecosystem Respiration, from Daytime partitioning method, reference selected from RECO versions using model efficiency (MEF). The MEF analysis is repeated for each time aggregation
HH	µmolCO2 m-2 s-1	
DD	gC m-2 d-1	calculated from half-hourly data
WW-MM	gC m-2 d-1	average from daily data
YY	gC m-2 y-1	sum from daily data
RECO_DT_VUT_USTAR50		Ecosystem Respiration, from Daytime partitioning method, based on NEE_VUT_USTAR50
HH	µmolCO2 m-2 s-1	
DD	gC m-2 d-1	calculated from half-hourly data
WW-MM	gC m-2 d-1	average from daily data
YY	gC m-2 y-1	sum from daily data
RECO_DT_VUT_MEAN		Ecosystem Respiration, from Daytime partitioning method, average from RECO versions, each from corresponding NEE_VUT_XX version
HH	µmolCO2 m-2 s-1	average from 40 half-hourly RECO_DT_VUT_XX
DD	gC m-2 d-1	average from 40 daily RECO_DT_VUT_XX
WW	gC m-2 d-1	average from 40 weekly RECO_DT_VUT_XX
MM	gC m-2 d-1	average from 40 monthly RECO_DT_VUT_XX
YY	gC m-2 y-1	average from 40 yearly RECO_DT_VUT_XX
RECO_DT_VUT_SE		Standard Error for Ecosystem Respiration, calculated as (SD(RECO_DT_VUT_XX) / SQRT(40))
HH	µmolCO2 m-2 s-1	SE from 40 half-hourly RECO_DT_VUT_XX
DD	gC m-2 d-1	SE from 40 daily RECO_DT_VUT_XX
WW	gC m-2 d-1	SE from 40 weekly RECO_DT_VUT_XX
MM	gC m-2 d-1	SE from 40 monthly RECO_DT_VUT_XX
YY	gC m-2 y-1	SE from 40 yearly RECO_DT_VUT_XX

RECO_DT_VUT_XX		Ecosystem Respiration, from Daytime partitioning method (with XX = 05, 16, 25, 50, 75, 84, 95)
HH	µmolCO2 m-2 s-1	
DD	gC m-2 d-1	calculated from half-hourly data
WW-MM	gC m-2 d-1	average from daily data
YY	gC m-2 y-1	sum from daily data
RECO_DT_CUT_REF		Ecosystem Respiration, from Daytime partitioning method, reference selected from RECO versions using model efficiency (MEF). The MEF analysis is repeated for each time aggregation
HH	µmolCO2 m-2 s-1	
DD	gC m-2 d-1	calculated from half-hourly data
WW-MM	gC m-2 d-1	average from daily data
YY	gC m-2 y-1	sum from daily data
RECO_DT_CUT_USTAR50		Ecosystem Respiration, from Daytime partitioning method, based on NEE_CUT_USTAR50
HH	µmolCO2 m-2 s-1	
DD	gC m-2 d-1	calculated from half-hourly data
WW-MM	gC m-2 d-1	average from daily data
YY	gC m-2 y-1	sum from daily data
RECO_DT_CUT_MEAN		Ecosystem Respiration, from Daytime partitioning method, average from RECO versions, each from corresponding NEE_CUT_XX version
HH	µmolCO2 m-2 s-1	average from 40 half-hourly RECO_DT_CUT_XX
DD	gC m-2 d-1	average from 40 daily RECO_DT_CUT_XX
WW	gC m-2 d-1	average from 40 weekly RECO_DT_CUT_XX
MM	gC m-2 d-1	average from 40 monthly RECO_DT_CUT_XX
YY	gC m-2 y-1	average from 40 yearly RECO_DT_CUT_XX
RECO_DT_CUT_SE		Standard Error for Ecosystem Respiration, calculated as (SD(RECO_DT_CUT_XX) / SQRT(40))

HH	μmolCO2 m-2 s-1	SE from 40 half-hourly RECO_DT_CUT_XX
DD	gC m-2 d-1	SE from 40 daily RECO_DT_CUT_XX
WW	gC m-2 d-1	SE from 40 weekly RECO_DT_CUT_XX
MM	gC m-2 d-1	SE from 40 monthly RECO_DT_CUT_XX
YY	gC m-2 y-1	SE from 40 yearly RECO_DT_CUT_XX
RECO_DT_CUT_XX		Ecosystem Respiration, from Daytime partitioning method (with XX = 05, 16, 25, 50, 75, 84, 95)
HH	μmolCO2 m-2 s-1	
DD	gC m-2 d-1	calculated from half-hourly data
WW-MM	gC m-2 d-1	average from daily data
YY	gC m-2 y-1	sum from daily data
GPP_DT_VUT_REF		Gross Primary Production, from Daytime partitioning method, reference selected from GPP versions using model efficiency (MEF). The MEF analysis is repeated for each time aggregation
HH	μmolCO2 m-2 s-1	
DD	gC m-2 d-1	calculated from half-hourly data
WW-MM	gC m-2 d-1	average from daily data
YY	gC m-2 y-1	sum from daily data
GPP_DT_VUT_USTAR50		Gross Primary Production, from Daytime partitioning method, based on NEE_VUT_USTAR50
HH	μmolCO2 m-2 s-1	
DD	gC m-2 d-1	calculated from half-hourly data
WW-MM	gC m-2 d-1	average from daily data
YY	gC m-2 y-1	sum from daily data
GPP_DT_VUT_MEAN		Gross Primary Production, from Daytime partitioning method, average from GPP versions, each from corresponding NEE_VUT_XX version
HH	μmolCO2 m-2 s-1	average from 40 half-hourly GPP_DT_VUT_XX
DD	gC m-2 d-1	average from 40 daily GPP_DT_VUT_XX
WW	gC m-2 d-1	average from 40 weekly GPP_DT_VUT_XX
MM	gC m-2 d-1	average from 40 monthly GPP_DT_VUT_XX

YY	gC m-2 y-1	average from 40 yearly GPP_DT_VUT_XX
GPP_DT_VUT_SE		Standard Error for Gross Primary Production, calculated as (SD(GPP_DT_VUT_XX) / SQRT(40))
HH	µmolCO2 m-2 s-1	SE from 40 half-hourly GPP_DT_VUT_XX
DD	gC m-2 d-1	SE from 40 daily GPP_DT_VUT_XX
WW	gC m-2 d-1	SE from 40 weekly GPP_DT_VUT_XX
MM	gC m-2 d-1	SE from 40 monthly GPP_DT_VUT_XX
YY	gC m-2 y-1	SE from 40 yearly GPP_DT_VUT_XX
GPP_DT_VUT_XX		Gross Primary Production, from Daytime partitioning method (with XX = 05, 16, 25, 50, 75, 84, 95)
HH	µmolCO2 m-2 s-1	
DD	gC m-2 d-1	calculated from half-hourly data
WW-MM	gC m-2 d-1	average from daily data
YY	gC m-2 y-1	sum from daily data
GPP_DT_CUT_REF		Gross Primary Production, from Daytime partitioning method, reference selected from GPP versions using model efficiency (MEF). The MEF analysis is repeated for each time aggregation
HH	µmolCO2 m-2 s-1	
DD	gC m-2 d-1	calculated from half-hourly data
WW-MM	gC m-2 d-1	average from daily data
YY	gC m-2 y-1	sum from daily data
GPP_DT_CUT_USTAR50		Gross Primary Production, from Daytime partitioning method, based on NEE_CUT_USTAR50
HH	µmolCO2 m-2 s-1	
DD	gC m-2 d-1	calculated from half-hourly data
WW-MM	gC m-2 d-1	average from daily data
YY	gC m-2 y-1	sum from daily data
GPP_DT_CUT_MEAN		Gross Primary Production, from Daytime partitioning method, average from GPP versions, each from corresponding NEE_CUT_XX version
HH	µmolCO2 m-2 s-1	average from 40 half-hourly GPP_DT_CUT_XX

DD	gC m-2 d-1	average from 40 daily GPP_DT_CUT_XX
WW	gC m-2 d-1	average from 40 weekly GPP_DT_CUT_XX
MM	gC m-2 d-1	average from 40 monthly GPP_DT_CUT_XX
YY	gC m-2 y-1	average from 40 yearly GPP_DT_CUT_XX
GPP_DT_CUT_SE		Standard Error for Gross Primary Production, calculated as (SD(GPP_DT_CUT_XX) / SQRT(40))
HH	µmolCO2 m-2 s-1	SE from 40 half-hourly GPP_DT_CUT_XX
DD	gC m-2 d-1	SE from 40 daily GPP_DT_CUT_XX
WW	gC m-2 d-1	SE from 40 weekly GPP_DT_CUT_XX
MM	gC m-2 d-1	SE from 40 monthly GPP_DT_CUT_XX
YY	gC m-2 y-1	SE from 40 yearly GPP_DT_CUT_XX
GPP_DT_CUT_XX		Gross Primary Production, from Daytime partitioning method (with XX = 05, 16, 25, 50, 75, 84, 95)
HH	µmolCO2 m-2 s-1	
DD	gC m-2 d-1	calculated from half-hourly data
WW-MM	gC m-2 d-1	average from daily data
YY	gC m-2 y-1	sum from daily data
PARTITIONING - SUNDOWN		
RECO_SR		Ecosystem Respiration, from Sundown Respiration partitioning method
HH	µmolCO2 m-2 s-1	
DD	gC m-2 d-1	calculated from half-hourly data
WW-MM	gC m-2 d-1	average from daily data
YY	gC m-2 y-1	sum from daily data
RECO_SR_N		Fraction between 0-1, indicating the percentage of data avaiable in the averaging period to parametrize the respiration model
HH		not available
DD-YY	nondimensional	percentage of data available

Table SM7. Predefined unit options. Except for COUNTRY, all options are listed for each variable to give users an understanding of what options were available (i.e., not all options exist in the dataset). BADM Vocabulary is the standard name used to describe pre-defined unit options in the BADM standards.

Variable (BADM Vocabulary)	Option	Description
ASPECT (ASPECT)	E	East
	ENE	East-northeast
	ESE	East-southeast
	FLAT	The site is flat and the footprint/exposure is not in any specific direction
	N	North
	NE	Northeast
	NNE	North-northeast
	NNW	North-northwest
	NW	Northwest
	S	South
	SE	Southeast
	SSE	South-southeast
	SSW	South-southwest
	SW	Southwest
	W	West
	WNW	West-northwest
	WSW	West-southwest
COUNTRY (COUNTRY)	Argentina	
	Austria	
	Australia	
	Belgium	
	Brazil	

	Canada	
	Republic of Congo	
	Switzerland	
	China	
	Czech Republic	
	Germany	
	Denmark	
	Spain	
	Finland	
	France	
	French Guyana	
	Ghana	
	Greenland	
	Italy	
	Japan	
	Malaysia	
	Netherlands	
	Panama	
	Russia	
	Sudan	
	Sweden	
	Svalbard and Jan Mayen	
	Senegal	
	USA	
	South Africa	
	Zambia	
WIND_DIRECTION	E	East

(DIR)	ENE	East-northeast
	ESE	East-southeast
	N	North
	NE	Northeast
	NNE	North-northeast
	NNW	North-northwest
	NW	Northwest
	S	South
	SE	Southeast
	SSE	South-southeast
	SSW	South-southwest
	SW	Southwest
	W	West
	WNW	West-northwest
	WSW	West-southwest
DOM_DIST_MGMT (DIST_MGMT)	Agriculture	Agricultural management of any kind, such as cultivation (including tillage, plowing, or discing), harvest, irrigation, pesticides, planting, or liming.
	Drought	Prolonged water deficiency; hydrologic or climatic drought.
	Fire	Wildfire or managed burns
	Forestry	Forest management such as logging of any kind, plantation planting, or herbicide application.
	Grazing	Herbivory or browsing by mammals, managed or wild.
	Hydrologic event	Drainage, persistent flooding, or chronic flooding. Does not include storm events or irrigation
	Land cover change	Land use and land cover change; invasion; woody or urban encroachment
	Pests and disease	Plant or soil damage from pests, insects, pathogens, blight, and other disease.

	Storm or wind	Major storms including unusually high-precipitation and unusually high-wind events e.g. hurricane, tornado, blizzard, hail, flooding from storm, etc.
	Temperature extreme	Heat wave or freeze
	Undisturbed	No disturbance or management has occurred on the site.
DOI_ORGANIZATION_ROLE (DOI_ORG_ROLE)	Originator	Organization that performed the research or owns the dataset. Normally the institution(s) of the tower team PI(s).
	Sponsor	Any company, institution, or organization which sponsored / funded the research.
DOI_CONTRIBUTOR_ROLE (DOI_ROLE)	Author	A person who should be listed in the DOI citation as an author.
	Other	A person whose role is not described by options in this predefined list.
FLUX_MEASUREMENTS_METHOD (FLUX_METHOD)	Chambers	
	Eddy Covariance	
	Gradients	
	Other	
	Scintillometer	
FLUX_MEASUREMENTS_OPERATIONS (FLUX_OPERATIONS)	Continuous operation	Variable collected continuously with the reported method.
	Growing season operation only	Variable collected only during the growing season with the reported method
	Intermittent	Variable collected intermittently with the reported method
	Periodic operation	Variable collected part of the year with the reported method
	Planned	The site or equipment to collect the specific variable is not yet operational
FLUX_MEASUREMENTS_VARIABLE (FLUX_VARIABLE)	Aerosols	
	BVOCs	
	CH4	

	CO2	
	H	
	H2O	
	Isotopes	
	N2O	
	O3	
	Other	
IGBP (IGBP)	BSV	Barren Sparse Vegetation: Lands exposed soil, sand, or rocks and has less than 10% vegetative cover during any time of the year.
	CRO	Croplands: Lands covered with temporary crops followed by harvest and a bare soil period (e.g., single and multiple cropping systems). Note that perennial woody crops will be classified as the appropriate forest or shrub land cover type.
	CSH	Closed Shrublands: Lands with woody vegetation less than 2 meters tall and with shrub canopy cover >60%. The shrub foliage can be either evergreen or deciduous.
	CVM	Cropland/Natural Vegetation Mosaics: Lands with a mosaic of croplands, forest, shrublands, and grasslands in which no one component comprises more than 60% of the landscape
	DBF	Deciduous Broadleaf Forests: Lands dominated by woody vegetation with a percent cover >60% and height exceeding 2 meters. Consists of broadleaf tree communities with an annual cycle of leaf-on and leaf-off periods.
	DNF	Deciduous Needleleaf Forests: Lands dominated by woody vegetation with a percent cover >60% and height exceeding 2 meters. Consists of seasonal needleleaf tree communities with an annual cycle of leaf-on and leaf-off periods.
	EBF	Evergreen Broadleaf Forests: Lands dominated by woody vegetation with a percent cover >60% and height exceeding 2 meters. Almost all trees and shrubs remain green year round. Canopy is never without green foliage.
	ENF	Evergreen Needleleaf Forests: Lands dominated by

		woody vegetation with a percent cover >60% and height exceeding 2 meters. Almost all trees remain green all year. Canopy is never without green foliage.
	GRA	Grasslands: Lands with herbaceous types of cover. Tree and shrub cover is less than 10%. Permanent wetlands lands with a permanent mixture of water and herbaceous or woody vegetation. The vegetation can be present in either salt, brackish, or fresh water.
	MF	Mixed Forests: Lands dominated by trees with a percent cover >60% and height exceeding 2 meters. Consists of tree communities with interspersed mixtures or mosaics of the other four forest types. None of the forest types exceeds 60% of landscape.
	OSH	Open Shrublands: Lands with woody vegetation less than 2 meters tall and with shrub canopy cover between 10-60%. The shrub foliage can be either evergreen or deciduous.
	SAV	Savannas: Lands with herbaceous and other understory systems, and with forest canopy cover between 10-30%. The forest cover height exceeds 2 meters.
	SNO	Snow and Ice: Lands under snow/ice cover most of the year.
	URB	Urban and Built-Up Lands: Land covered by buildings and other man-made structures.
	WAT	Water Bodies : Oceans, seas, lakes, reservoirs, and rivers. Can be either fresh or salt- water bodies.
	WET	Permanent Wetlands: Lands with a permanent mixture of water and herbaceous or woody vegetation that cover extensive areas. The vegetation can be present in either salt, brackish, or fresh water
	WSA	Woody Savannas: Lands with herbaceous and other understory systems, and with forest canopy cover between 30-60%. The forest cover height exceeds 2 meters.
VAR_INFO_MODEL<br><br>(INST_MODEL)	GA_CP_SA-Other	Gas Analyzer, Closed Path with Sonic Anemometer - Other
	GA_CP-Aerodyne	Gas Analyzer, Closed Path Fast Response, Aerodyne

	GA_CP-Campbell EC155	Gas Analyzer, Closed Path, Campbell EC155
	GA_CP-Campbell TGA100	Gas Analyzer, Closed Path, Campbell TGA100
	GA_CP-Campbell TGA200A	Gas Analyzer, Closed Path, Campbell TGA200A
	GA_CP-LGR 911-0001	Gas Analyzer, Closed Path, Los Gatos Research 911-0001 for CH4/H2O
	GA_CP-LGR 911-0010	Gas Analyzer, Closed Path, Los Gatos Research 911-0010 for CH4/CO2/H2O
	GA_CP-LGR 911-0020	Gas Analyzer, Closed Path, Los Gatos Research 911-0020 for CO2/H2O
	GA_CP-LGR 913-0014	Gas Analyzer, Closed Path, Los Gatos Research 913-0014 for N2O/CO/H2O
	GA_CP-LGR 913-0029	Gas Analyzer, Closed Path, Los Gatos Research 913-0029 and 907-0029 for CO/CO2/H2O
	GA_CP-LGR 913-1054	Gas Analyzer, Closed Path, Los Gatos Research 913-1054 for N2O/CH4/H2O
	GA_CP-LGR 914-0028	Gas Analyzer, Closed Path, Los Gatos Research 914-0028 and 907-0028 for OCS/CO2/CO/H2O
	GA_CP-LGR 914-1012	Gas Analyzer, Closed Path, Los Gatos Research 914-1012 and 914-0012 for NH3/H2O
	GA_CP-LGR Other	Gas Analyzer, Closed Path, Los Gatos Research Other Model
	GA_CP-LGR RMT-200	Gas Analyzer, Closed Path, Los Gatos Research RMT-200
	GA_CP-LI-COR LI-6252	Gas Analyzer, Closed Path, LI-COR LI-6252
	GA_CP-LI-COR LI-6262	Gas Analyzer, Closed Path, LI-COR LI-6262
	GA_CP-LI-COR LI-7000	Gas Analyzer, Closed Path, LI-COR LI-7000
	GA_CP-LI-COR LI-7200	Gas Analyzer, Closed Path, LI-COR LI-7200
	GA_CP-LI-COR LI-7200RS	Gas Analyzer, Closed Path, LI-COR LI-7200RS

	GA_CP-Other	Gas Analyzer, Closed Path Fast Response - Other
	GA_CP-Picarro G1301-f	Gas Analyzer, Closed Path, Picarro G1301-f
	GA_CP-Picarro G2301-f	Gas Analyzer, Closed Path, Picarro G2301-f
	GA_CP-Picarro G2311-f	Gas Analyzer, Closed Path, Picarro G2311-f
	GA_CP-Picarro Other	Gas Analyzer, Closed Path, Picarro Other Model
	GA_OP_SA-Campbell IRGASON	Gas Analyzer, Open Path with Sonic Anemometer, Campbell IRGASON
	GA_OP_SA-Other	Gas Analyzer, Open Path with Sonic Anemometer - Other
	GA_OP-Campbell EC150	Gas Analyzer, Open Path, Campbell EC150
	GA_OP-Krypton Hygrometer	Gas Analyzer, Open Path, Krypton Hygrometer
	GA_OP-LI-COR LI-7500	Gas Analyzer, Open Path, LI-COR LI-7500
	GA_OP-LI-COR LI-7500A	Gas Analyzer, Open Path, LI-COR LI-7500A
	GA_OP-LI-COR LI-7500DS	Gas Analyzer, Open Path, LI-COR LI-7500DS
	GA_OP-LI-COR LI-7500RS	Gas Analyzer, Open Path, LI-COR LI-7500RS
	GA_OP-LI-COR LI-7700	Gas Analyzer, Open Path, LI-COR LI-7700
	GA_OP-Lyman-alpha Hygrometer	Gas Analyzer, Open Path, Lyman-alpha Hygrometer
	GA_OP-Other	Gas Analyzer, Open Path Fast Response - Other
	GA_SR-LI-COR LI-800	Gas Analyzer, Slow Response, LI-COR LI-800
	GA_SR-LI-COR LI-8100	Gas Analyzer, Slow Response, LI-COR LI-8100
	GA_SR-LI-COR LI-8100A	Gas Analyzer, Slow Response, LI-COR LI-8100A

	GA_SR-LI-COR LI-820	Gas Analyzer, Slow Response, LI-COR LI-820
	GA_SR-LI-COR LI-840	Gas Analyzer, Slow Response, LI-COR LI-840
	GA_SR-LI-COR LI-840A	Gas Analyzer, Slow Response, LI-COR LI-840A
	GA_SR-Other	Gas Analyzer, Slow Response - Other
	GA_SR-Picarro G2401	Gas Analyzer, Slow Response, Picarro G2401
	GA-Other	Gas Analyzer - Other
	SA-ATI	Sonic Anemometer - ATI
	SA-ATI CATI/2	Sonic Anemometer - ATI CATI/2
	SA-ATI SATI A Style	Sonic Anemometer - ATI SATI A Style
	SA-ATI SATI K Style	Sonic Anemometer - ATI SATI K Style including SWS-211/3K
	SA-ATI SPAS	Sonic Anemometer - ATI SPAS
	SA-ATI Sx Style	Sonic Anemometer - ATI Sx Style
	SA-ATI V Style	Sonic Anemometer - ATI V Style
	SA-ATI Vx Style	Sonic Anemometer - ATI Vx Style
	SA-Campbell CSAT-3	Sonic Anemometer - Campbell CSAT-3
	SA-Campbell CSAT-3A	Sonic Anemometer - Campbell CSAT-3A
	SA-Campbell CSAT-3B	Sonic Anemometer - Campbell CSAT-3B
	SA-Gill HS-100	Sonic Anemometer - Gill HS-100
	SA-Gill HS-50	Sonic Anemometer - Gill HS-50
	SA-Gill R2	Sonic Anemometer - Gill R2
	SA-Gill R3-100	Sonic Anemometer - Gill R3-100
	SA-Gill R3-50	Sonic Anemometer - Gill R3-50
	SA-Gill R3A-100	Sonic Anemometer - Gill R3A-100
	SA-Gill Windmaster	Sonic Anemometer - Gill Windmaster

	SA-Gill Windmaster HS	Sonic Anemometer - Gill Windmaster HS
	SA-Gill Windmaster Pro	Sonic Anemometer - Gill Windmaster Pro2
	SA-Metek USA-1 Fast	Sonic Anemometer - Metek USA-1 Fast
	SA-Metek uSonic-3 Class A	Sonic Anemometer - Metek uSonic-3 Class A
	SA-Metek uSonic-3 Omni	Sonic Anemometer - Metek uSonic-3 Omni
	SA-Metek uSonic-3 Scientific	Sonic Anemometer - Metek uSonic-3 Scientific (formerly USA-1)
	SA-Other	Sonic Anemometer - Other
	SA-Young 81000	Sonic Anemometer - Young 81000
	SA-Young 81000RE	Sonic Anemometer - Young 81000RE
	SA-Young 81000V	Sonic Anemometer - Young 81000V
	SA-Young 81000VRE	Sonic Anemometer - Young 81000VRE
LAND_OWNERSHIP (LAND_OWNERSHIP)	private	Land ownership: private
	public	Land ownership: public
NETWORK (NETWORK)	AmeriFlux	http://ameriflux.lbl.gov
	AsiaFlux	http://www.asiaflux.net/
	CarboAfrica	http://www.carboafrica.net/
	CarboEuroFlux	
	CarboEuropeIP	
	CarboExtreme	http://www.carboextreme.eu
	CarboItaly	
	Carbomont	http://www.uibk.ac.at/carbomont/
	ChinaFLUX	http://www.chinaflux.org/en/index/index.asp
	EuroFlux	http://www.unitus.it/dipartimenti/disafri/progetti/eflux/euro.html
	Fluxnet-Canada	http://fluxnet.ccrp.ec.gc.ca

	GHG-Europe	http://www.ghg-europe.eu/
	GreenGrass	
	ICOS	http://www.icos-ri.eu/
	IMECC	http://imecc.ipsl.jussieu.fr/
	InGOS	http://www.ingos-infrastructure.eu/
	JapanFlux	http://www.japanflux.org/
	KoFlux	
	LBA	http://www.lbaeco.org/lbaeco/index.html
	LTAR	https://ltar.ars.usda.gov/
	LTER	http://www.lternet.edu/
	Medeflu	
	MexFlux	
	NEON	http://www.neoninc.org/
	OzFlux	http://www.ozflux.org.au/
	PAGE21	http://www.page21.eu/
	Phenocam	https://phenocam.sr.unh.edu/webcam/
	Swiss FluxNet	http://www.gl.ethz.ch/research/bage/fluxnet-ch.html
	TaiwanFlux	http://140.112.63.212/tflux.html
	TCOS-Siberia	http://www.bgc.mpg.de/public/carboeur/web_TCOS/
	TERENO	http://teodoor.icg.kfa-juelich.de/overview-en
	ThaiFlux	http://compete.center.ku.ac.th/HomeFlux.htm
	TROPI-DRY	http://tropi-dry.eas.ualberta.ca/
	Unaffiliated	
	Urban Flux Network	http://www.geog.ubc.ca/urbanflux/
	USCCC	http://research.eeescience.utoledo.edu/lees/researc h/usccc/
REFERENCE_USAGE<br>(REFERENCE_USAG	Alt_Citation	Additional papers that could be used as citations for the site.

E)	Analysis_Result	Paper describing results of an analysis performed at the site or using the data from the site.
	Background	Useful paper for understanding the history and background of the site.
	Primary_Citation	Paper recommended to be used as a citation for the site.
	Reference	Useful paper for understanding the site and/or measurements at the site.
TEAM_MEMBER_ROLE (TEAM_ROLE)	Affiliate	Affiliated site member: additional staff members.
	AncContact	Ancillary data contact: person in addition to the PI to be contacted regarding site data
	BADMContact	BADM data contact: person to be contacted regarding biological, soil, disturbance and management and other site information
	DataManager	Data Manager: person to be contacted regarding data preparation and submission.
	FluxContact	Flux-met data contact: person to be contacted regarding micrometeorological measurements.
	PI	Principal Investigator: person primarily responsible for the site that should be contacted for data policy and other important issues and also CC'ed in all the communications with the team members. At least one PI must be designated.
	Technician	Technician responsible for maintaining site operations
TERRAIN (TERRAIN)	Flat	Site is flat with no significant land surface changes
	Gentle slope (<2 %)	
	Hilltop	Site is on the top of a hill
	Medium Slope (>2 %, <5%)	
	Significant Slope (>5%, <10%)	
	Strong Slope (>10%)	
	Undulated/Variable	
	Valley	Site is in a valley, with slopes all around

TOWER_POWER (TOWER_POWER)	Direct power	A/C power connection
	Gasoline generator	
	Methanol generator	
	Other	Other type of power source
	Solar + generator	
	Solar panels	Solar panels and batteries
	Wind generator	
TOWER_TYPE (TOWER_TYPE)	other	Other type of tower
	pole	
	triangle	
	tripod	
	walk-up	
